# Tuning potency for precision: the role of the G4-ligand in G4-ligand conjugated oligonucleotides targeting individual G-quadruplex DNA structures

**DOI:** 10.1039/d5cb00302d

**Published:** 2026-02-10

**Authors:** Alva Abrahamsson, Sakina Khwaja, Andreas Berner, Rabindra Nath Das, Koit Aasumets, Namrata Chaudhari, Sjoerd Wanrooij, Erik Chorell

**Affiliations:** a Department of Chemistry, Umeå University SE-901 87 Umeå Sweden erik.chorellumu.se; b Department of Medicinal Biochemistry and Biophysics, Umeå University SE-907 36 Umeå Sweden sjoerd.wanrooijumu.se

## Abstract

The non-B DNA secondary structure known as G-quadruplex (G4) DNA can form in gene-regulatory regions of the human genome. Organic small molecules, so called G4-ligands, which bind and stabilize G4 DNA structures, have emerged as potential therapeutic agents and as chemical probes to study the cellular functions of these secondary DNA conformations. A major challenge, however, is their lack of selectively recognising different G4 structures, which hampers both their further development as therapeutics and their utility as research tools. This limitation can be addressed by linking the G4-ligand to a guide oligonucleotide, complementary to the flanking sequence of the target G4, a strategy called G4-ligand-conjugated oligonucleotides (GL-Os). Here, the G4-ligand used in the GL-O strategy is investigated by altering its physiochemical properties, including size and charge, and evaluating its potency using biochemical and biological assays. The results reveal a strong influence of the G4-ligand on the conjugates’ ability to bind and stabilize the target G4 DNA. In particular, a larger and charged G4-ligand enhances both binding and stabilization of the target. However, increasing the G4-ligand potency may also increase off-target G4 binding, highlighting the need for a balanced G4-ligand potency to ensure high GL-O specificity for individual G4 structures.

## Abstract 2

G4-ligands with varying chemical profiles were incorporated into G4-ligand conjugated oligonucleotides (GL-Os) and evaluated for G-quadruplex binding and stabilization, revealing how ligand chemistry shapes potency and specificity.

## Graphical abstract

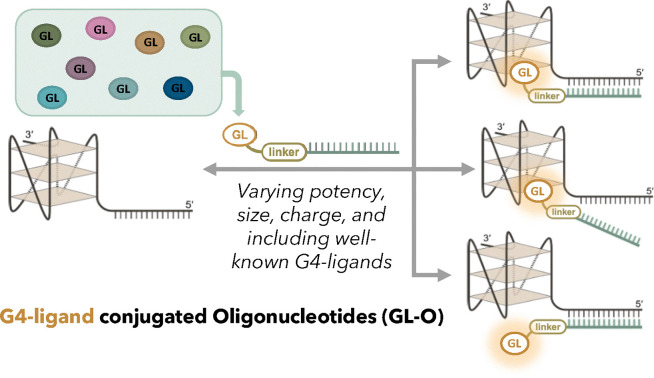

## Introduction

G-quadruplex DNA structures (G4s) are canonical or non-canonical secondary conformations that can form within guanine-rich sequences.^1^ These structures are formed by the planar arrangement of four guanine residues into a G-tetrad, which can self-stack into stable higher-order structures. The formation and stability of G4s depend on Hoogsteen hydrogen bonding, arene-arene stacking interactions, and coordination with monovalent or divalent cations.^2^ Intra-G4 formation can occur within sequences containing four consecutive guanines represented as G_≥3_N_*x*_G_≥3_N_*x*_G_≥3_N_*x*_G_≥3_, where N denotes other nucleobases forming the loops. Structural variation among G4s is influenced by factors such as the strand orientation, loop length and sequence composition, resulting in diverse G4 topologies.^2,3^

G4 formation requires disruption of the double helical DNA, which typically occurs during transcription, replication or DNA damage repair.^1^ Computational analysis and sequencing methods have identified over 700 000 potential G4 DNA structures within the human genome,^4^ concentrated in regions such as telomeric ends, replication origins, and promotor regions of regulatory genes, including oncogenes. These G4s are thought to play critical roles in cellular function.^1,2,5^ For instance, the oncogene *c-MYC*, dysregulated in approximately 70% of human cancers,^6^ contains a G-rich region able to form a G4 called Pu27, in the region upstream of the P1 promotor.^7^ Small molecules, known as G4-ligands, can bind and stabilize Pu27, suppressing *c-MYC* expression and thereby inhibiting cancer growth.^7,8^ Targeting oncogenic G4 motifs, such as those in *c-MYC*, *c-KIT*,^9^*K-Ras*^10^ among others, has emerged as a promising therapeutic strategy for cancer treatment.

Currently, over a thousand G4-ligands are registered,^11^ developed as chemical probes to investigate the cellular functions of G4s or as starting points for drug discovery.^12^ These ligands typically feature aromatic or heteroaromatic cores able to form arene–arene interactions with the end G-tetrads. Furthermore, cationic groups are frequently present in G4 ligands and can enhance binding and stabilization.^13–15^ While many G4-ligands effectively distinguish between double-stranded DNA (dsDNA) and G4 DNA, achieving inter-G4 selectivity *i.e.* the ability to differentiate between various G4 motifs, remains a persistent challenge in the field.^16,17^

**Fig. 1:**
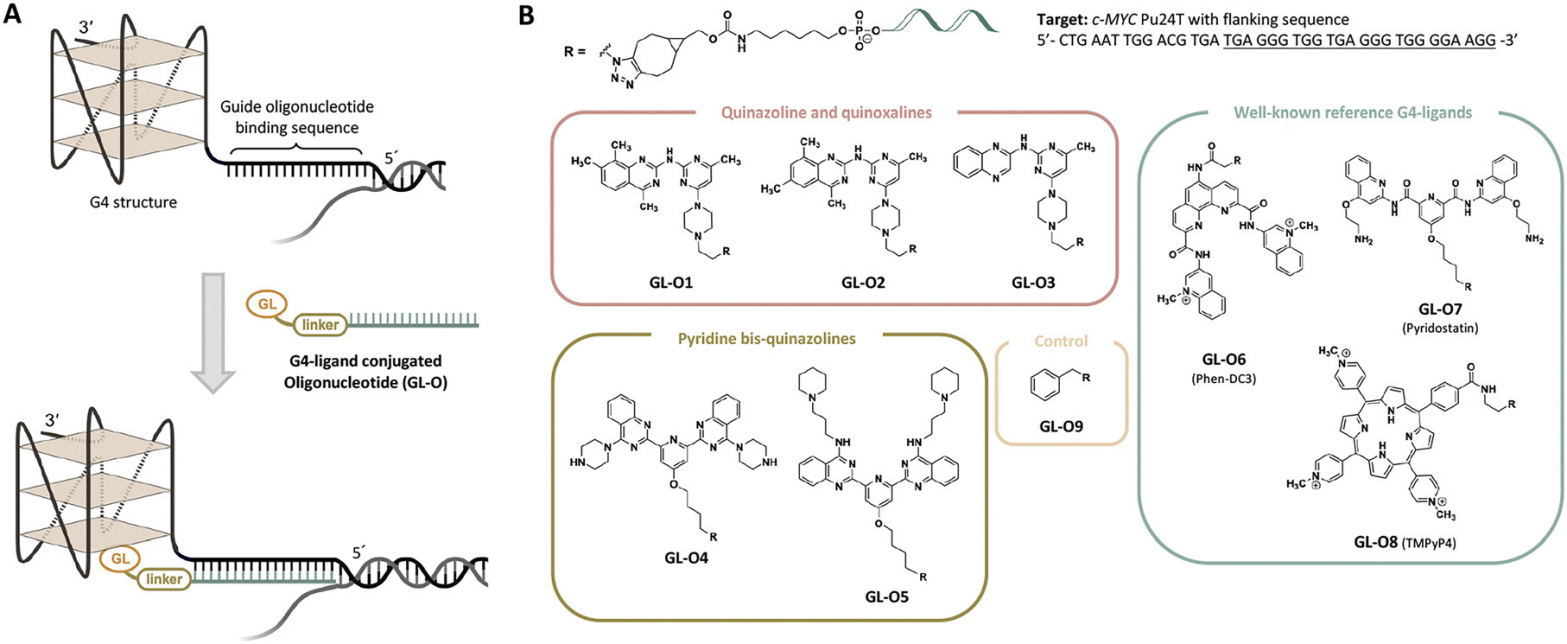
Overview of the G4-ligand conjugated Oligonucleotide strategy and the G4-ligand modified GL-O conjugates part of this study. (A) Outline of the GL-O strategy targeting individual G4 structures using the guide oligonucleotide as recognition motif. (B) GL-Os 1–9 developed for this study with modified G4-ligands consisting of three categories and a control (benzyl). The figure includes the target G4 DNA sequence.

A potential solution to the selectivity issue involves linking the G4-specific ligand to a short DNA fragment, an oligonucleotide, that is complementary to the sequence flanking the target G4 structure.^18^ This oligonucleotide serves as a guide, directing the G4-ligand to bind and stabilize only the specific G4 target with the matching flanking sequence, while minimizing interactions with non-matching G4 targets. The approach, termed the G4-ligand conjugated oligonucleotide (GL-O) strategy, was recently reported by us (Fig. 1A).^18^ The GL-O approach offers flexibility, as the oligonucleotide can be customized to complement the flanking sequence of any desired G4 target.

Furthermore, the linker connecting the oligonucleotide and G4-ligand can be adjusted to accommodate varying distances between the guide oligonucleotide binding sequence and the G4 structure (Fig. 1A).^19^ However, central to the GL-O approach is the G4-ligand that induces the G4 stabilization. In this study, the choice of G4-ligand is investigated in terms of binding and stabilization properties with the hypothesis that this plays a crucial role in determining the overall effectiveness of the GL-O strategy.

To test this hypothesis, we designed new GL-O conjugates with chemically different G4-ligands, both in terms of binding affinity, stabilization capacity, size, and charge to evaluate their impact on G4 binding and stabilization when conjugated to the guide oligonucleotide (Fig. 1). For comparison, three well-known G4-ligands; Phen-DC3,^20^ Pyridostatin^21^ and TMPyP4,^22^ were included (Fig. 1B). This study focuses on the biophysical and biochemical evaluation of the G4-ligand's role in the overall ability of GL-O conjugates to bind and stabilize target G4 structures.

To achieve this, G4-ligands were functionalized with terminal azides to enable oligonucleotide conjugation. In accordance with the conjugation method and linker composition of the most recent GL-O,^19^ the G4-ligands were conjugated to a 5′-hexylamine oligonucleotide *via* strain-promoted azide–alkyne cycloaddition (SPAAC). The resulting GL-Os 1–9 were assessed for binding affinity and interaction properties with the G4 target using microscale thermophoresis (MST) and proton nuclear magnetic resonance spectroscopy (^1^H NMR). In addition, thermal stabilization of the G4 structure in complex with the GL-Os was evaluated by ^1^H NMR and further validated using DNA polymerase stop assay that measures polymerase stalling at stabilized G4 sites. Together, these analyses revealed that the physicochemical properties of the G4-ligand critically determine the conjugate's ability to recognize and stabilize its G4 target. Larger and positively charged ligands enhanced both binding and stabilization, yet excessive ligand potency increased the risk of off-target G4 interactions. A less potent ligand benefit from the guide oligonucleotide hybridization, positioning the ligand in proximity to the G4 binding site. These findings highlight the need to balance ligand strength to achieve optimal GL-O specificity toward individual G4 structures. Although cellular selectivity is a central long-term objective, this study focuses on elucidating the previously unexplored role of the G4-ligand in modulating GL-O binding selectivity and potency *in vitro*.

## Result and discussion

### Design and synthesis of azide-functionalized G4-ligands and oligonucleotide conjugation

To investigate how the choice of G4-ligand influences the efficiency of the resulting GL-O constructs, a set of G4-ligands were selected based on both drug-like properties such as size, charge, and symmetry, but also their reported G4 binding and stabilization capabilities. Understanding how G4-ligand properties modulate GL-O behavior is important, as this represents a valuable possibility to tune the overall GL-O strategy to improve pharmacokinetic properties like cell permeability. Furthermore, investigating the binding affinity and G4 stabilization efficiency of these GL-Os is critical since highly potent G4-ligands can increase the risk of nonspecific binding of unintended G4 structures. However, previous work has demonstrated that GL-Os exhibit synergistic activity, where a pronounced effect is only observed when both the G4-ligand and the guide oligonucleotide simultaneously engage their respective targets.^18^ This synergism inherently reduces the likelihood of nonspecific G4-ligand activity, as the large oligonucleotide component must correctly hybridize for full activity to occur.

Moreover, it is of particular interest to assess whether a weak G4-ligand can still exert a significant stabilizing effect when placed in the right location by the guide oligonucleotide. This is analogous to increasing the local concentration of the ligand at the target site, potentially enhancing efficacy while minimizing systemic off-target effects.

**Scheme 1:**
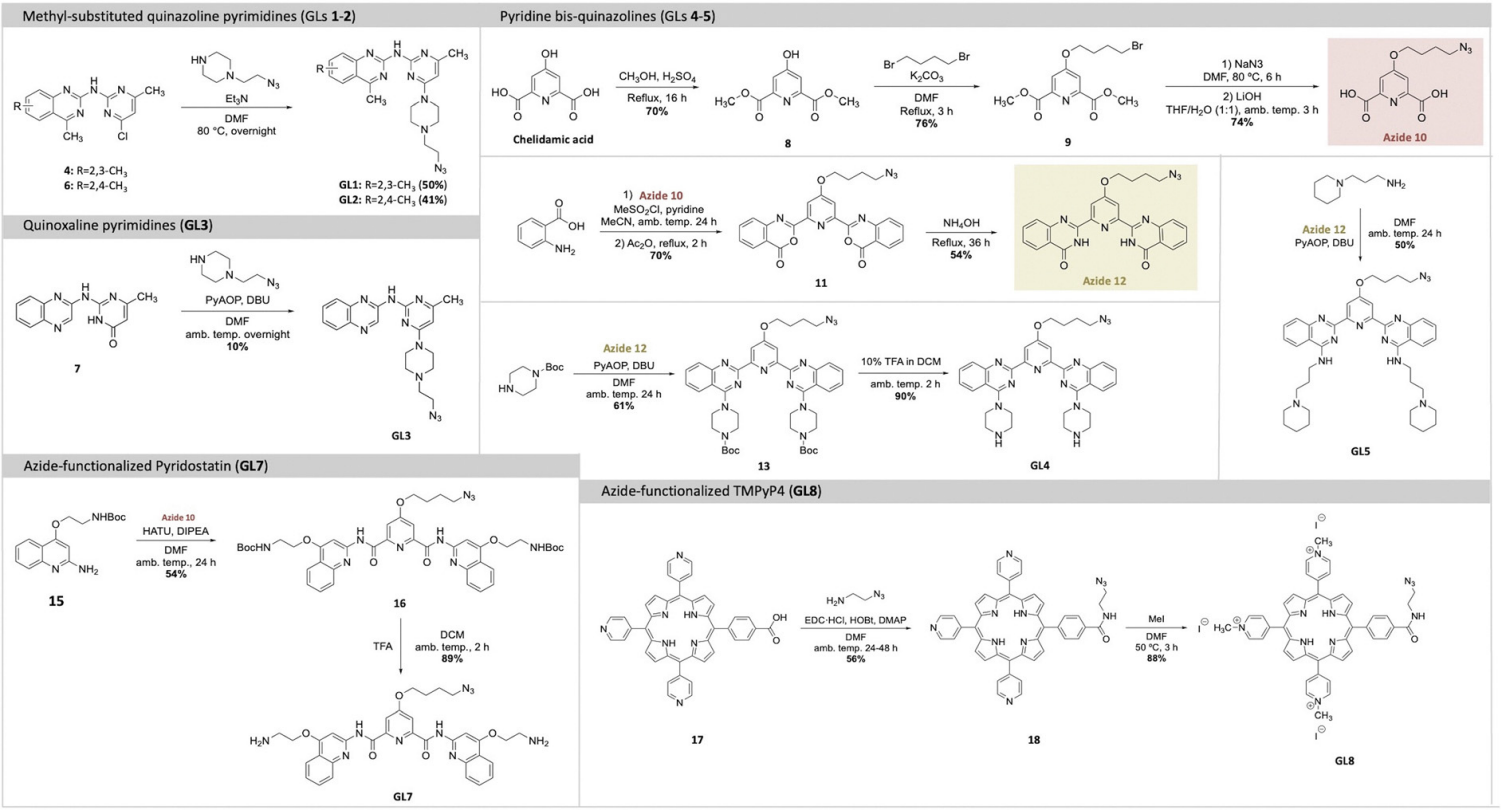
Total synthetic scheme of the azide-functionalized G4-ligands (GLs 1–5, 7–8).

**Scheme 2:**
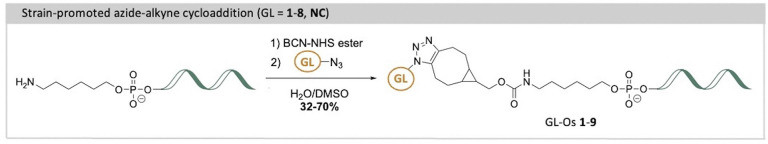
Conjugation of G4-ligands with the oligonucleotide through strain-promoted azide–alkyne cycloaddition.

The introduction of new G4-ligands to the GL-O approach require modified G4-ligand cores with a functional group that can be used for conjugation with the guide oligonucleotide. To achieve this, methods to synthesize the selected G4-ligands (GLs 1–5, 7–8) with terminal azides were developed to later allow for oligonucleotide conjugations *via* SPAAC to generate the target GL-Os 1–9 (Schemes 1 and 2).

The G4-ligands based on quinazoline- and quinoxaline-pyrimidine scaffolds (GLs 1–3) were selected for their favorable drug-like characteristics, including small molecular size, absence of *C*2 symmetry, and lack of permanent charge. GLs 1–2 are known to be highly potent G4 stabilizers, whereas GL3, despite its close structural similarity, exhibits minimal G4-stabilizing activity. This contrast provides a valuable opportunity to investigate how subtle structural differences that tune G4-ligand potency influence the performance of the corresponding GL-O constructs. For all these derivatives, the piperazine-azide side chain, used in the first-generation GL-O conjugates,^18,19^ was retained to maintain the potential for a positively charged moiety at physiological pH. GLs 1–2 were synthesized using SNAr reactions starting from chlorinated scaffolds, yielding GL1 and GL2 in 50% and 41% yield, respectively, while GL3 was obtained through a PyAOP-mediated SNAr with a 10% yield (Scheme 1).

The pyridine bis-quinazoline G4-ligands (GLs 4–5) represent a newly developed class of highly potent G4-stabilizing agents.^23^ These ligands are based on a *C*2-symmetric architecture, in which two quinazoline units are connected through a central pyridine core, allowing for potential multivalent G4 interactions. Importantly, GLs 4–5 retain key drug-like properties, such as the absence of permanent charges, which may support favorable pharmacokinetic characteristics. Their strong G4-stabilizing activity across different G4 structures makes them attractive candidates for use in GL-O constructs. Compared to the smaller, non-symmetric ligands GLs 1–3, these bis-quinazoline compounds offer a different structural framework, enabling evaluation of how molecular symmetry and scaffold architecture influence G4 recognition, GL-O efficacy, and selectivity.

GLs 4–5 were synthesized starting from azide containing precursor 12 (Scheme 1). Chelidamic acid was methylated and functionalized with a brominated side chain, which was subsequently converted to azide-contaning compound 10. Reacting compound 10 with anthranilic acid yielded bis-benzoxazine 11, which was aminated to produce bis-quinazolinone 12. GL4 was synthesized by reacting 12 with 1-boc-piperazine through a PyAOP-mediated SNAr, followed by Boc deprotection using 10% v/v TFA in DCM, in 90% yield. Similarly, GL5 was prepared by reacting 12 with 1-(3-aminopropyl)piperidine in a PyAOP-mediated SNAr, which afforded GL5 in 50% yield (Scheme 1).

To place these ligands in context and benchmark their performance, we also included well-characterized G4-binding compounds: Phen-DC3, Pyridostatin and TMPyP4. These ligands are widely used reference compounds in the G4 field due to their strong G4 affinity and stabilizing capabilities, though they are all *C*2-symmetric and exhibit limited drug-like properties, with Phen-DC3 and TMPyP4 carrying permanent charges. Including these ligands enables direct comparison with the other scaffolds (GLs 1–5) in terms of G4-stabilization, selectivity, and potential for off-target interactions.

The Phen-DC3 azide analogue (GL6) was synthesized by previously described procedures.^24^ To synthesize the Pyridostatin azide-analogue (GL7), azide 10 was coupled to compound 15 through HATU-activated amide coupling, yielding compound 16 in 54% yield (Scheme 1). Subsequent Boc deprotection using TFA produced GL7 in 89% yield. The porphyrin-based TMPyP4 azide-analogue (GL8), was synthesized starting from a carboxylate analogue of TMPyP4, previously described by Spagnul *et al.*^25^ The azide side-chain was first linked using EDC and HOBt-mediated amide coupling to yield 18 in 56% yield, followed by pyridine methylation with MeI to achieve GL8 in 88% yield (Scheme 1). In addition to these G4-ligands, a benzyl control compound (NC) lacking G4-binding functionality was used to assess background effects and to ensure that any observed activity in GL-O constructs is indeed G4-specific and not driven by non-specific interactions or linker-related contributions.

The azide-functionalized G4-ligands were then conjugated with the oligonucleotide using the previously described SPAAC method^19^ (Scheme 2), affording GL-Os 1–9, in yields reported in Table S4. The slight variations in the connection between the G4-ligands and the linker to the guide oligonucleotide are unlikely to significantly influence comparisons among the different G4-ligands in the GL-Os. Because the G4-ligands are connected to the guide oligonucleotide through an already long and conformationally flexible linker and are separated from the G4 core by a three-nucleotide gap, modest variations in the connections on the G4-ligand are not expected to substantially affect binding affinity or selectivity, consistent with our previous systematic linker analysis.^19^ Together, this diverse set of ligands enables a comprehensive evaluation of how structural features such as symmetry, charge, and known G4-binding profiles impact GL-O performance, target specificity, and potential off-target liabilities.

### Binding properties of the G4-ligand modified GL-Os

The G4 target investigated in this study is the mutated *c-MYC* G4 known as Pu24T. This sequence has a 15-nucleotide flanking sequence at the 5′-end of the G4-forming sequence (Fig. 1B and Table S1). Throughout the study, the term G4 DNA is referred to *c-MYC* Pu24T along with the flanking sequence.

**Fig. 2:**
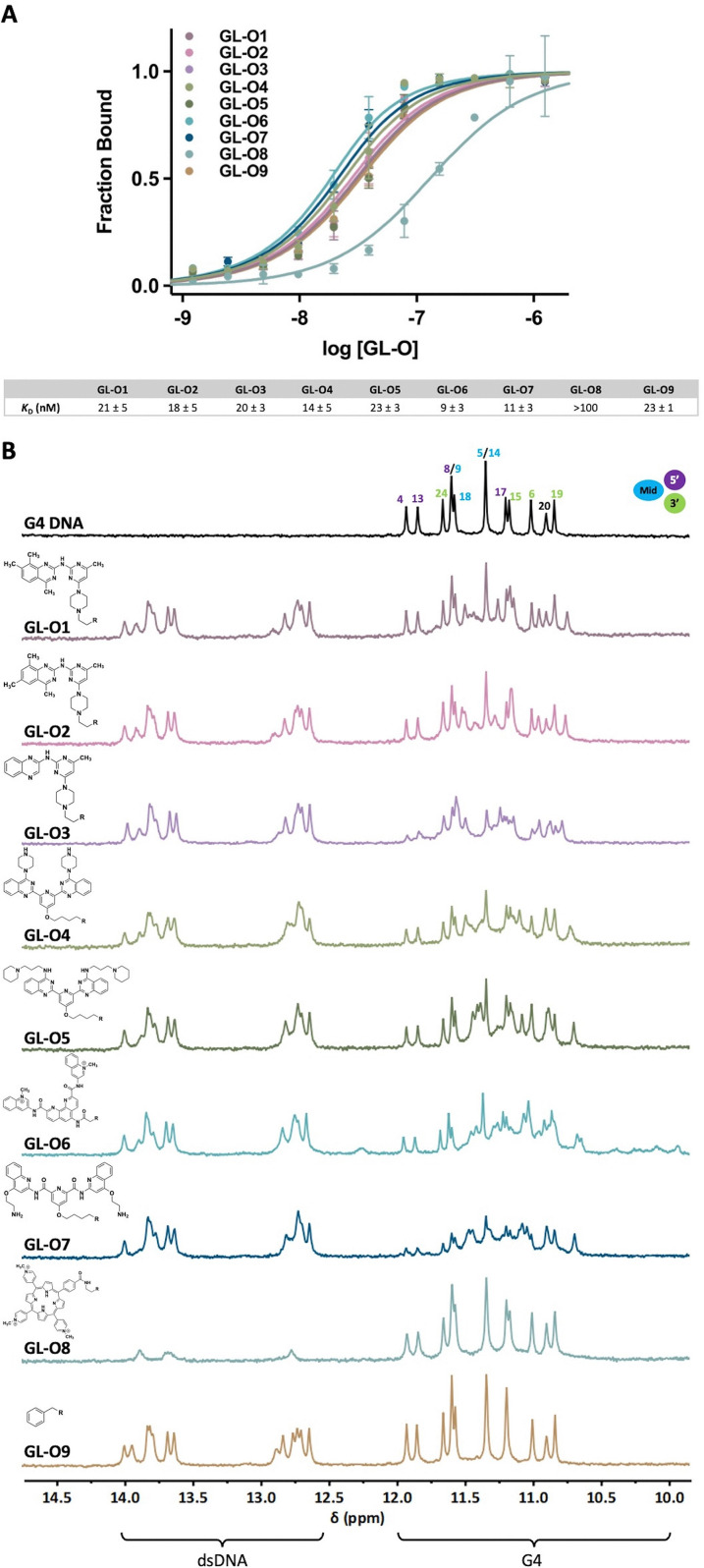
Effect of the G4-ligand modified GL-Os on their binding to G4 DNA, R is the linker and oligonucleotide. (A) Dose–response curves obtained using MST where the GL-Os 1–9 were serial diluted to the G4 DNA 5′-fluorescently labelled *c-MYC* Pu24T G4 DNA. Dissociation constants (*K*_D_) values and error bars correspond to two independent measurements. (B) ^1^H NMR profiles of the G4 DNA (black) and GL-Os 1–9 in 1 : 1 molar ratios with *c-MYC* Pu24T with complementary flanking sequence (90 µM). G4 imino proton signals appear between 10–12 ppm and double-stranded DNA imino proton signals between 12–14 ppm.

Binding affinities of GL-O conjugates (GL-Os 1–9) to the target G4 DNA were first evaluated using MST. Dissociation constants (*K*_D_) were determined from dose–response curves by measuring the interaction between each GL-O and a fluorescently labeled G4 DNA (Table S1). Most GL-Os (1–7 and 9) bound efficiently to the G4 DNA, with *K*_D_ values ranging from 9 to 23 nM (Fig. 2A). In contrast, GL-O8 with the TMPyP4 analogue, exhibited a substantially weaker interaction with a *K*_D_ value exceeding 100 nM.

The binding affinity of the azide-functionalized G4-ligands alone (GLs 1–8 and NC, without oligonucleotide conjugation) were significantly poorer, reflecting higher *K*_D_ values (Fig. S1A). Among these, the effective small drug-like G4-ligands GLs 1–2 showed strong affinities, with *K*_D_ values of 57 and 150 nM, respectively. The structurally similar but less active G4-ligand GL3, displayed a markedly reduced affinity (*K*_D_ value around 1400 nM). For GL6, the Phen-DC3 analogue, a two-site binding event was observed (Fig. S1B), while its GL-O counterpart, GL-O6, exhibited a single-site binding profile (Fig. 2A). This observation aligns with the intended design of GL-Os, where the guide oligonucleotide efficiently directs the G4-ligand to a specific target site *via* hybridization, effectively restricting the binding to a defined position which enhance target specificity.

This two-site tendency was true for the larger ligands; however, the lower nanomolar-range *K*_D_ binding site was more difficult to determine, most likely due to physicochemical limitations such as poor solubility and aggregation (Fig. S1A).

Overall, G4-ligand conjugation to the oligonucleotide to form GL-Os, led to an improved binding. This enhancement is likely attributed both to the significantly improved solubility of these derivatives and to the additional Watson–Crick base-pairing provided by the guide oligonucleotide. This effect, initially observed with the first developed GL-O,^18^ appears to be generalizable across chemically diverse G4-ligands, underscoring a key advantage of the GL-O platform in rescuing the performance of otherwise suboptimal G4-ligands.

To obtain more detailed information on the binding event and assess if the G4-ligand modified GL-Os interact differently with the G4 structure, ^1^H NMR was employed (Fig. 2B). In ^1^H NMR, the imino proton signals can be used to distinguish between the G4 and dsDNA (10–12 ppm for G4 DNA and 12–14 ppm for dsDNA). This allows simultaneous evaluation of both G4-ligand interaction with the G4 structure and oligonucleotide hybridization. GL-Os 1–7 and 9 displayed successful hybridization with the flanking sequence, as evidenced by the appearance of duplex-characteristic imino signals. In contrast, these signals (12–14) ppm were nearly absent for GL-O8. Additionally, GL-O8 displayed an imino proton pattern in the G4 DNA region (10–12 ppm) that closely resembled the negative control (GL-O9), indicating a lack of G4 interaction. A plausible explanation to this inactivity can be linked to the G4-ligand used in GL-O8, TMPyP4, which is known to also bind single-stranded DNA,^26^ along with other problematic concerns.^12^ This may lead to intra- or intermolecular self-interactions between the ligand and its conjugated oligonucleotide, thereby inhibiting hybridization with the target flanking sequence, evident by the lack of dsDNA proton peaks. As a result, this G4-ligand is unavailable to interact with the target G4 structure.

GL-Os 1–2 exhibited comparable interaction patterns, consistent with their identical scaffolds, showing broadening of the original G4 peaks (10–12 ppm) along with the appearance of new G4-ligand bound signals. This indicate that small modifications of the G4-ligand, like shifting position of one methyl group in GL1*vs*. GL2 does not significantly influence their binding modes as GL-Os (Fig. 2B). GL-O3, derived from a weaker G4-ligand, has a slightly different binding pattern compared to GL-Os 1 and 2 but retains the strong broadening of the original G4 peaks displayed by GL3 alone (Fig. S2 *vs*. Fig. 2). GL-Os 4–5, carrying pyridine bis-quinazoline G4-ligands with different amine side chains, show similar interaction profiles. Binding of GL-O5 results in the formation of a clean new set of G4 imino peaks, indicating a distinct binding mode (Fig. 2B). Binding of GL-Os 6–7, corresponding to Phen-DC3 and Pyridostatin analogues, result in strong effects on the G4 imino signals. GL-O7, based on Pyridostatin, exhibits extensive peak broadening and formation of a set of new G4 imino proton peaks, likely from the G4-ligand bound state (Fig. 2B). Interestingly, the G4-ligand GL6 alone (Phen-DC3 analogue) showed extensive shifts in the G4 imino proton signals (Fig. S2). Upon conjugation into GL-O6, these shifts were significantly decreased, which is in line with the GL-O conjugation that prevent unspecific binding and force the G4-ligand to bind in a specific area (compare Fig. S2 and Fig. 2B).

Overall, the oligonucleotide conjugation of the G4-ligands enhances the resolution of the NMR spectra which likely is linked to an increased solubility of the GL-Os compared to the G4-ligands. Furthermore, the oligonucleotide conjugation also selectively positions the G4-ligands at the 5′-tetrad of the G4 structure and thereby prevent potential off-target interactions, *e.g.* intercalating or with the 3′-tetrad of the G4 structure, which further increase the resolution.

### Stabilization of the G4-ligand modified GL-Os

**Fig. 3:**
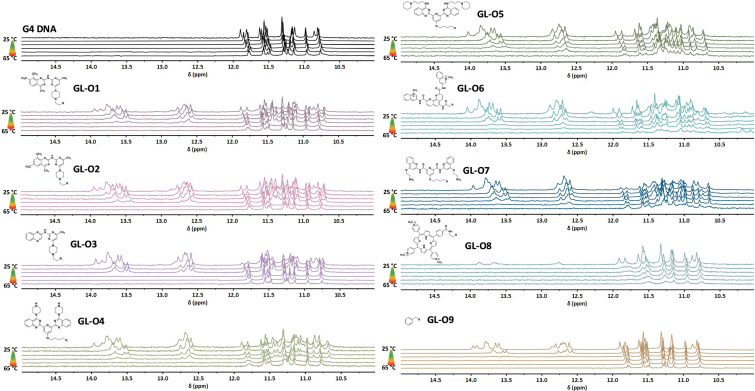
Thermal stabilization of the c-MYC Pu24T G4 DNA structure by G4-ligand modified GL-Os 1–9 to c-MYC Pu24T, R is the linker and oligonucleotide. Thermal melting analysis using ^1^H NMR. Spectra were recorded of c-MYC Pu24T with complementary 5′-flanking sequence with a 5 °C temperature ramp starting from 45 °C and compared with the G4 DNA alone (black). G4 imino signals appear between 10–12 ppm and double-stranded DNA signals appear between 12–14 ppm.

The thermal stability of the G4 DNA in presence of the GL-Os 1–9 was assessed by using ^1^H NMR, with spectra recorded using a stepwise temperature increase of 5 °C (Fig. 3). Both dsDNA and G4 imino proton signals were monitored to assess stabilization through both oligonucleotide hybridization and G4-ligand interactions, as previously reported.^18^

The G4 imino proton signals remained relatively stable throughout the tested temperature range, whereas the duplex DNA imino signals (used as indicators of hybridization between the guide oligonucleotide and the 5′-flanking sequence) showed greater sensitivity to thermal disruption. For example, the duplex signals associated with the benzyl-substituted control GL-O9 began to disappear at 45 °C and were fully absent by 50 °C, indicating complete melting of the duplex structure (Fig. 3).

Addition of an efficient G4-ligand to the guide oligonucleotide simultaneously increases the thermal stability both the G4 DNA and the guide oligonucleotide hybridization with the 5′-flanking sequence. The GL-O containing a weak ligand (GL-O3, based on a quinoxaline scaffold) retained duplex DNA signals up to 55 °C, a 5 °C increase over the benzyl-substituted control. The use of more potent G4-ligand in the GL-O, such as the methyl-substituted quinazoline-based ligands in GL-Os 1–2, also translates into a more efficient stabilization of the dsDNA with a complete melting at 60 °C. These results demonstrates that although the GL-O positions a weaker G4-ligand, such as in GL-O3, in the correct position for binding, it does not necessarily translate into a stronger G4 stabilization unless intrinsic ligand potency is sufficient.

The trend also holds with the larger, *C2*-symmetric ligands: GL-Os 4–5 (bis-quinazoline), GL-O6 (Phen-DC3), and GL-O7 (Pyridostatin), all of which conferred complete duplex stabilization up to 60–65 °C (Fig. 3). Among these, a notable distinction was observed between GL-Os 4–5, which differ only in side-chain composition of the G4-ligand. While both displayed similar binding affinities, GL-O5 (with extended side-chain linker) exhibited improved thermal stabilization. This discrepancy between binding and stabilization has been reported for many G4-ligands and appear to extend to the GL-O platform. Furthermore, these findings highlight how even minor structural modifications to the G4-ligand can significantly influence the overall GL-O performance, an important observation given that the G4-ligand constitutes only a small fraction of the total GL-O construct.

The stabilization effect of the GL-Os on the G4 DNA is complex to interpret with ^1^H NMR due to the intrinsic thermal stability of the G4 structure and the lack of complete melting below 65 °C. However, evidence of ligand interaction was apparent through peak broadening and the emergence of new G4 imino signals. Overall, the G4 stabilization induced by the GL-Os mirror the trend observed for the individual G4-ligands (Fig. S3), as well as the corresponding scaffolds.^15^ Notably, weaker ligands lost their G4 interactions at elevated temperatures, coinciding with the dissociation of the guide oligonucleotide. In contrast, the more potent ligands in *e.g.*GL-O5 and GL-O7, retained G4-binding features even after duplex melting, indicating that these ligands can maintain their G4 interaction independently of oligonucleotide hybridization. An open question remains whether persistent G4 binding after duplex dissociation is beneficial. While sustained G4 interaction may enhance on-target efficacy, a well-tuned GL-O that dissociates from both the G4 core and the flanking region under the same conditions could offer advantages in minimizing off-target G4 stabilization, which is an important consideration that merits further investigation.

The GL-O incorporating the TMPyP4-derived G4-ligand, GL-O8, exhibited no thermal stabilization, consistent with its poor binding properties at a 1 : 1 molar ratio with G4 DNA (Fig. 3). Interestingly, the G4 seems to melt almost completely, which could be a unfolding tendency due to TMPyP4 (Fig. 3 and Fig. S3), which has been previously reported.^27^

To further investigate the effect of the G4-ligand in the GL-O constructs on G4 DNA stabilization under biologically relevant conditions, a DNA polymerase stop assay was applied. In this assay, a Taq polymerase is allowed to synthesize DNA using a DNA template containing the G4-forming Pu24T sequence along with its flanking region (Table S1). During primer extension, the polymerase encounters the G4 structure, which serves as a structural barrier and induced polymerase stalling due to its intrinsic stability. However, some of the DNA polymerase can bypass the G4 and continue elongation to generate a full-length DNA product, quantified as percentage full-length product (FL). GL-Os that effectively bind and stabilize the G4 structure are expected to increase polymerase stalling, thereby reducing the amount of full-length DNA product.

**Fig. 4:**
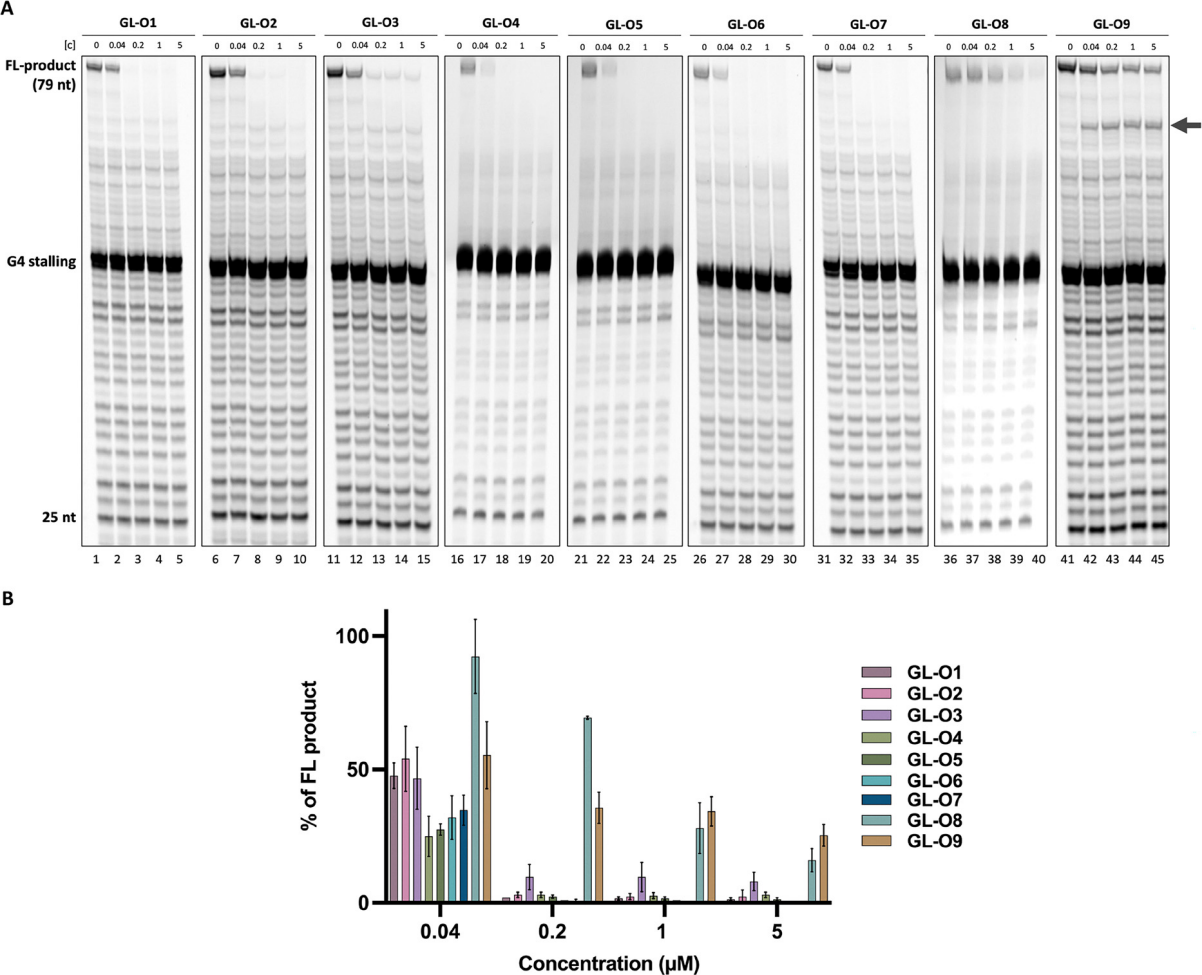
Polymerase stabilization of the G4 DNA in presence of the GL-Os 1–9. (A) A representative gel from a Taq polymerase stop assay with increasing concentration of (0.04 to 5 µM) of GL-O1 (lanes 1–5), GL-O2 (lanes 6–10), GL-O3 (lanes 11–15), GL-O4 (lanes 16–20), GL-O5 (lanes 21–25), GL-O6 (lanes 26–30), GL-O7 (31–35), GL-O8 (lanes 36–40) and GL-O9 (lanes 41–45). (B) Quantification of the Taq polymerase stop assay shown in a. The full-length (FL, 79 nt) DNA product is expressed as a% of the full-length band intensity that was observed in the control reaction containing only the G4 template. Data represent the mean ± standard deviation from three independent experiments. The arrow indicates the 64 nt band corresponding to oligonucleotide stalling.

The experimental design permitted the DNA polymerase to initiate synthesis from the 25-nt TET-labelled primer, encountering the G4 structure at the 20^th^ base. All tested GL-Os 1–8 demonstrated strong dose-dependent G4 stabilization, leading to a pronounced decrease in full-length DNA product (Fig. 4). At the lowest concentration (0.04 µM, approximately 1 : 1 molar ratio to DNA), all of the GL-Os, except GL-O8, showed comparable stabilization with a trend toward stronger effects or GL-Os 4–7 (Fig. 4). The GL-O containing the weakest G4-ligand, GL-O3, appeared similarly active at this concentration, however, it never achieved complete polymerase stalling, even at the highest concentration tested. Already at 0.2 µM (approximately fivefold excess), GL-O3 shows a markedly lower stabilization compared to the other GL-Os, consistent with the weaker G4-ligand properties of GL3 (Fig. S4B). Nonetheless, the oligonucleotide positions it correctly for G4 interaction, making it a reasonably good stabilizing GL-O overall. The control GL-O, GL-O9, which does not bind the G4, showed a moderate reduction of the full-length product, however this effect arises from oligonucleotide hybridization rather than G4 binding, as evidenced by the prominent 70-nt band that is absent for GL-Os 1–8 (Fig. 4A). Finally, GL-O8, consistently with binding affinity data does not stabilize the G4.

The Taq polymerase stop assay indicates that the *c-MYC* Pu24T sequence has a high intrinsic G4-forming propensity even in the absence of GL-Os, as shown by the prominent stalling band (Fig. 4A). To examine whether ligand-modified GL-Os could promote G4 formation under less favorable folding conditions, the assay was repeated in potassium-free buffer for GL-O3, GL-O7, and the control GL-O9 (Fig. S5). Under these conditions, G4-dependent polymerase stalling is clearly reduced compared with standard potassium conditions, consistent with reduced G4 stability. Upon addition of the GL-Os, increased polymerase stalling at the G4 site is again observed. However, since a measurable level of stalling remains detectable even in the absence of both potassium and GL-Os, these data cannot be interpreted as direct evidence that GL-Os actively induce *de novo* G4 formation. Rather, the results are consistent with stabilization of transiently or weakly populated G4 conformations, shifting the equilibrium toward the folded G4 state. Notably, the relative potency trend between GL-O3 and GL-O7 is maintained under potassium-free conditions (Fig. S5).

### Probing potential unspecific binding from the G4-ligand in the GL-Os

Up until now, data suggest that GL-Os incorporating the most potent G4-ligands (bis-quinazoline in GL-O5, Phen-DC3 analogue in GL-O6, and the Pyridostatin analogue in GL-O7), can maintain G4 interaction independently of oligonucleotide hybridization. This ligand-driven behavior suggests a potential for off-target binding and stabilization of non-complementary G4 DNA structures.

**Fig. 5:**
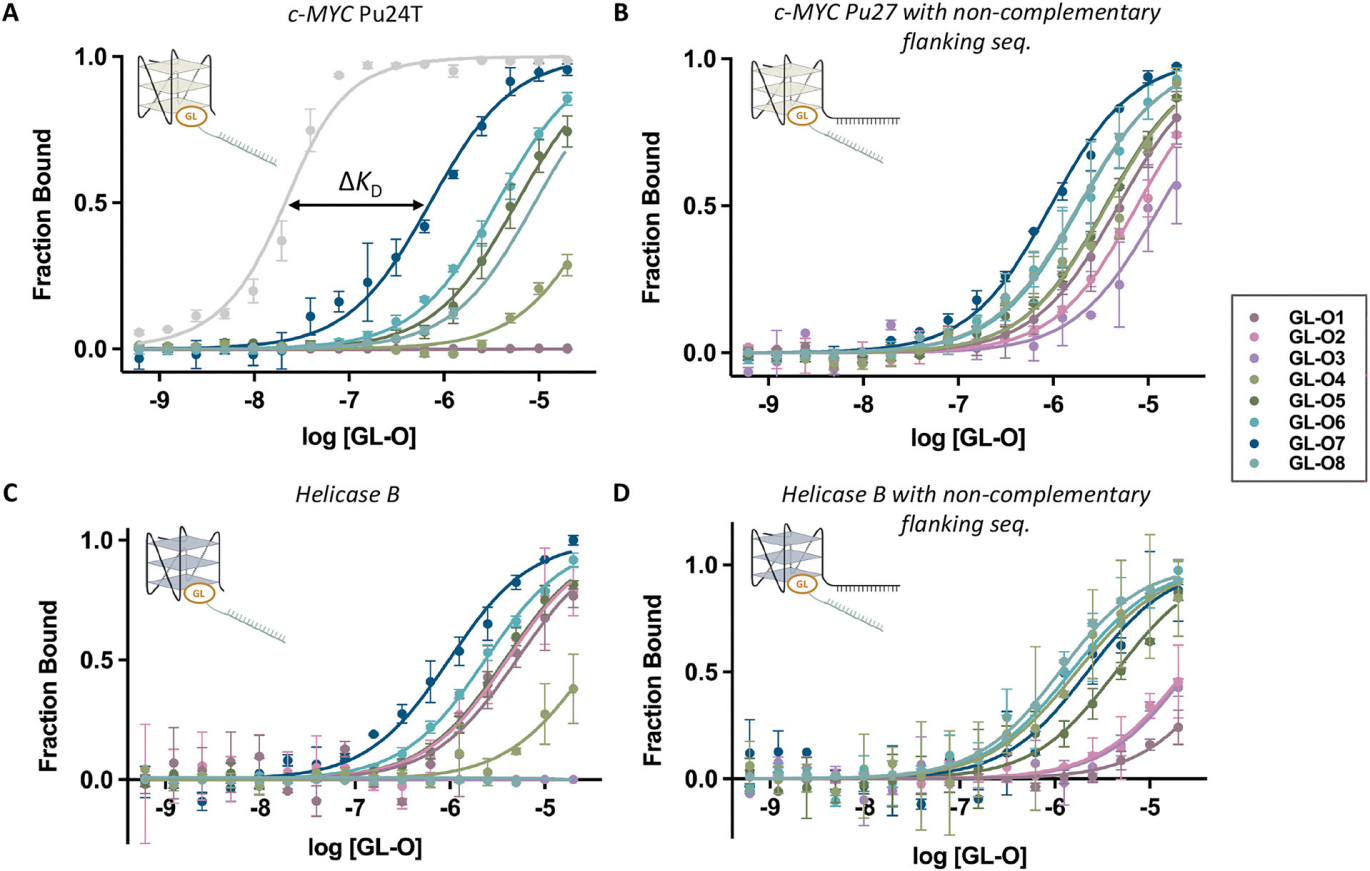
Effect of the G4-ligand modified GL-Os on their binding to a non G4 DNA targets. Dose–response curves obtained using MST where the GL-Os 1–8 were serial diluted to 5′-fluorescently labelled: (A) *c-MYC* Pu24T (without flanking sequence), with the light grey curve corresponding to GL-O7 with the correct complementary flanking sequence from Fig. 2A, (B) *c-MYC* Pu27 with non-complementary flanking sequence to the oligo in the GL-Os, (C) *Helicase B* G4–1 (without flanking sequence), and (D) *Helicase B* G4–1 with non-complementary flanking sequence to the oligo in the GL-Os. Dissociation constants (*K*_D_) values and error bars correspond to two independent measurements.

To further evaluate this possibility, we first removed the complementary flanking sequence from the *c-MYC* Pu24T G4 DNA structure (Table S1), and binding affinities of the GL-Os were measured (Fig. 5A, *K*_D_ values in Table S3). For some GL-Os, the binding curve lacked an upper plateau and *K*_D_ values are not accurately determined. GL-Os 1–3, as well as the control GL-O9, showed no detectable binding to this truncated G4, consistent with earlier observations from the first-generation GL-O containing a similar ligand core.^18^ In contrast, GL-Os 4–8 exhibit varying degrees of binding affinity, which correlated with their respective G4-ligand potencies. Neither the control GL-O, GL-O9, nor the oligonucleotide alone demonstrated any detectable binding (Fig. S6). Importantly, the binding affinities observed for GL-Os 4–8 were significantly lower (around 1000-folds), than the nanomolar affinities detected when hybridization was possible.

To confirm these observations, we evaluated interactions with the endogenous sequence of the c-MYC promotor G4, Pu27, with its flanking sequence (not complementary to the oligonucleotide of the GL-O) (Fig. 5B). For this target, all of the GL-Os 1–8 showed binding, although the potent GL-Os 6–7 exhibited higher *K*_D_ values than the less potent GL-O 1–3 (Table S3). For this target, the control GL-O, GL-O9, showed some binding affinity, likely due to the linker since the unconjugated oligonucleotide showed no binding affinity (Fig. S7).

Lastly, another parallel topology G4 structure: *Helicase B* G4–1 was investigated with and without flanking sequence (Fig. 5C and D, Table S1). For the construct lacking the flanking region, GL-Os 1–2 exhibited comparable binding affinities, whereas the less potent GL-O3 showed no detectable binding (Fig. 5C). A similar trend was observed for the pyridine bis-quinazolines G4-ligand GL-Os, where GL-O4 showed tendency of reduced binding affinity compared to the larger GL-O5. For this G4 target, the unconjugated oligonucleotide showed no detectable binding, whereas the control GL-O9 displayed a weak, nonspecific interaction (Fig. S8). For *Helicase B* with flanking sequence, the affinities were in general lower but with similar trends of the less potent GL-Os 1–3 not reaching a saturated plateau at the selected concentration span (Fig. 5D, *K*_D_ values in Table S3), with no binding of the control and unconjugated oligonucleotides (Fig. S9).

Together, these data reveal a general trend across both tested G4 targets: in the absence of hybridization, GL-Os contaning more potent and promiscuous G4-ligands are capable of inducing G4 binding. However, such non-specific interactions occur only at significantly higher concentrations compared to the nanomolar affinities observed when both the guide oligonucleotide and the G4-ligand engage the G4 structure cooperatively. Notably, this behavior was not universal; for example, GL-O7 showed minimal or no binding to the *c-Kit* 2 G4 structure (Fig. S10).

Overall, these findings indicate that off-target binding, where the G4-ligand within a GL-O interacts with G4 structures independently of guide hybridization, is possible but not inevitable, even for highly potent ligands. They highlight the importance of careful ligand selection in the GL-O design to minimize unintended G4 interactions while preserving high target specificity. Moreover, the herein presented data underscore the high selectivity of the GL-O strategy for its intended G4 target, which can be further refined and optimized through rational ligand selections.

## Conclusions

The G4-ligand is a central component of the GL-O strategy, as it directly governs G4 structure stabilization, while the conjugated oligonucleotide ensures precise delivery of the ligand to its intended G4 target. To examine the key factors governing the G4:G4-ligand interactions and stabilization, we have designed a novel set of GL-O conjugates incorporating various chemically diverse G4-ligands. Their properties were evaluated by assessing binding affinity, G4 interactions and stabilization capacity, using both thermal and enzymatic assays.

Conjugation of a G4-ligand into a GL-O drastically change physicochemical properties such as molecular weight and solubility which becomes highly dependent on the dominating oligonucleotide. Furthermore, this also changes the circumstances for G4-binding and stabilization as the oligonucleotide will place the G4-ligand in the correct position for binding the target G4 structure. To explore how to navigate these prerequisites in the selection of the optimal G4-ligand for the GL-O strategy, a diverse selection of different types of G4-ligands were explored: (1) compounds with drug-like characteristics, like low molecular weight and no permanent charges, ranging from highly efficient G4-ligands to weak G4-ligands through minimal chemical modifications of the core structure, (2) larger and highly potent *C2*-symmetric ligands with different side chains, and (3) a set well-known reference G4-ligands. These ligands were first synthesized with terminal azides to facilitate the click-conjugation with the oligonucleotide. Biophysical and biochemical evaluation of the newly synthesized GL-Os revealed that the G4-ligand is the primary mediator of G4 targeting, with modifications to its chemical composition significantly influencing binding interactions and stabilization outcomes. Larger, and in some cases, permanently charged ligands such as those in GL-Os 5–7, benefitted from oligonucleotide conjugation through improved solubility properties and simultaneously exhibited strong binding interactions alongside remarkable stabilization capabilities. Conversely, the weakly active but chemically similar G4-ligand in GL-O3 demonstrated G4 interactions similarly to the potent G4-ligand modified GL-Os. Although poor G4 stabilization, showing that the correct positioning of the G4-ligand close to its target binding site through the oligonucleotide annealing did not substantially enhance its weak G4 stabilization properties. Notably, not all previously reported G4-ligands did transfer into efficient GL-Os, as the TMPyP4 analogue GL-O8 failed to hybridize and interact with the G4 at a 1 : 1 molar ratio to G4 DNA.

The NMR studies of G4 stabilization at higher temperatures revealed that the G4-ligands in the most potent GL-Os were able to retain G4 stabilization even at temperatures where the guide oligonucleotide had dissociated. This indicate that these GL-Os might be able to bind G4 structures independent of guide oligonucleotide annealing and thereby lead to unwanted off-target binding. By using binding affinity measurements with different G4s, we could conclude that this is in fact possible although the selectivity window to these off-target interactions is large (100–1000-folds higher concentrations) even for the most potent G4-ligands used. Furthermore, the use of different G4-ligands showed that this effect can be tuned by the selection of G4-ligand and with the smaller and more drug-like G4-ligands, no off-target binding was observed.

In conclusion, this study provides further insight into the interactions between G4-ligands and the G4 structure within the GL-O framework, highlighting their central role in exploring diverse G4 DNA targets. By fine-tuning the GL-O strategy through modifications of the G4-ligand, this approach not only opens opportunities to investigate the biological functions of individual G4s, a capability that is currently largely lacking, but also lays the foundation for its development as a future therapeutic modality.

## Experimental procedure

### Organic synthesis

All reagents and solvents were purchased from commercial suppliers unless stated otherwise. TLC was performed on aluminium backed silica gel plates (median pore size 60 Å, fluorescent indicator 254 nm) and detected with UV light. Flash column chromatography was performed using silica gel with an average particle diameter of 50 µm (range 40–65 µm, pore diameter 53 Å), or aluminium oxide 150 basic (63–200 µm), eluents are given in brackets. ^1^H and ^13^C NMR spectra for characterization were recorded on a Bruker 400 MHz spectrometer at 298 K or on a Bruker 600 MHz spectrometer at 298 K and calibrated by using the residual peak of the solvents as the internal standard (CDCl_3_: *δ H* = 7.26 ppm; *δ C* = 77.16 ppm. DMSO-*d*_6_: *δ H* = 2.50 ppm; *δ C* = 39.50 ppm). LC-MS was conducted on an Agilent 6150 Series Quadrupole LC/MS system. HR-MS was performed by using an Agilent 1290 binary LC System connected to an Agilent 6230 Accurate-Mass TOF LC/MS (ESI^+^); calibrated with Agilent G1969-85001 ESTOF Reference Mix containing ammonium trifluoroacetate, purine and hexakis (1*H*, 1*H*, 3*H* tetrafluoropropoxy) phosphazine in 90 : 10 ACN/H_2_O.

### 2,2,4,7,8-pentamethyl-1,2-dihydroquinoline (1)

A microwave vial was charged with anhydrous MgSO_4_ (3.03 g, 25.2 mmol), 4-*tert*-butylcatechol (24.9 mg, 0.15 mmol), and I_2_ (63.45 mg, 0.25 mmol) subsequently. Then, 2,3-dimethylaniline (0.62 mL, 5.00 mmol) and acetone (13 mL) were added, and the mixture was heated to 110 °C in a sealed tube and let stir for 24 h. After cooling down, the mixture was filtered over a plug of Celite with EtOAc and the resulting organic solution was concentrated under reduced pressure. Purification by flash SiO_2_ column chromatography (eluent: 2.5% EtOAc in *n*-heptane) afforded compound 1 as a red-brown oil (0.87 g, 86%). ^1^H NMR (400 MHz, Chloroform-*d*) *δ* 6.89 (d, *J* = 7.7 Hz, 1H), 6.50 (d, *J* = 7.8 Hz, 1H), 5.28 (d, *J* = 1.5 Hz, 1H), 3.63 (s, 1H), 2.25 (s, 3H), 2.02 (s, 3H), 1.99 (d, *J* = 1.4 Hz, 3H), 1.29 (s, 6H). ^13^C NMR (100 MHz, Chloroform-*d*) δ 141.18, 136.39, 129.05, 127.08, 121.03, 119.27, 118.53, 118.04, 51.93, 31.47, 20.86, 18.92, 12.37.

### 1-(4,7,8-trimethylquinazolin-2-yl)guanidine (2)

Quinoline 1 (1.81 g, 8.99 mmol) was mixed with 2-cyanoguanidine (1.52 g, 18.1 mmol) in a RBF (25 mL). Then, 2 M HCl (5 mL) was added, and the mixture was stirred at 110 °C overnight. The mixture was then allowed to cool to ambient temperature and basified with NaOH (15%, 2.9 mL) and further diluted with water. The mixture was then sonicated, and the formed precipitate was then collected with suction filtration and washed with more water and Et_2_O to afford compound 2 as a white solid (1.67 g, 82%). ^1^H NMR (400 MHz, DMSO-*d*_6_) *δ* 8.39 (s, 4H), 7.91 (d, *J* = 8.5 Hz, 1H), 7.36 (d, *J* = 8.4 Hz, 1H), 2.46 (s, 6H). ^13^C NMR (100 MHz, DMSO-*d*_6_) *δ* 170.71, 156.71, 147.80, 142.70, 130.98, 127.67, 122.83, 118.70, 21.52, 20.58, 12.94.

### 6-methyl-2-((4,7,8-trimethylquinazolin-2-yl)amino)pyrimidin-4(3*H*)-one (3)

A RBF (50 mL) was charged with compound 2 (1.53 g, 6.66 mmol), followed by DMF (12.2 mL), DIPEA (2.32 mL, 13.3 mmol) and ethyl acetoacetate (8.50 mL, 67.2 mmol) sequentially. The solution was stirred at 120 °C for 2.5 h. The mixture was then diluted with Et_2_O and sonicated. The precipitate was collected with suction filtration and washed with more Et_2_O to afford desired compound 3 as a pale beige solid (1.13 g, 57%). ^1^H NMR (400 MHz, DMSO-*d*_6_) *δ* 13.64 (s, 1H), 11.28 (s, 1H), 7.95 (d, *J* = 8.3 Hz, 1H), 7.39 (d, *J* = 8.4 Hz, 1H), 5.82 (s, 1H), 2.84 (s, 3H), 2.53 (s, 3H), 2.47 (s, 3H), 2.17 (s, 3H).

### *N*-(4-chloro-6-methylpyrimidin-2-yl)-4,7,8-trimethylquinazolin-2-amine (4)

POCl_3_ (2.5 mL) was added to quinazoline 3 (0.50 g, 1.69 mmol) and refluxed in a sealed microwave vial at 100 °C for 24 h. Progress of the reaction was monitored using TLC. On completion, the reaction mixture was allowed to cool and the excess of POCl_3_ was evaporated under reduced pressure. The crude was treated with ice cold water and neutralized with 1N NaHCO_3_ solution. After neutralization the reaction mixture was subjected to extraction with ethyl acetate (3 × 50 mL). The combined organic extracts were dried over anhydrous sodium sulphate and concentrated under reduced pressure to give a yellow solid powder which on washing with Et_2_O yielded compound 4 (0.23 g, 44%). ^1^H NMR (400 MHz, Chloroform-*d*) *δ* 8.15 (s, 1H), 7.74 (d, *J* = 8.4 Hz, 1H), 7.25 (d, *J* = 8.2 Hz, 1H), 6.83 (s, 1H), 2.85 (s, 3H), 2.72 (s, 3H), 2.51 (s, 3H), 2.48 (s, 3H).

### *N*-(4-(4-(2-azidoethyl)piperazin-1-yl)-6-methylpyrimidin-2-yl)-4,7,8-trimethylquinazolin-2-amine (GL1)

Quinazoline 4 (100 mg, 0.32 mmol) was charged with piperazine-azide (133 mg, 0.49 mmol) in dry DMF (2.3 mL) followed by TEA (0.11 mL, 0.81 mmol). The reaction was heated at 80 °C overnight and monitored through LC-MS. On completion, reaction mixture was allowed to cool, subjected to evaporation of DMF and purified through flash column chromatography in basic alumina (eluent: 0.1% to 0.3% MeOH in DCM). Light yellow product GL1 was obtained (68 mg, 50% yield). ^1^H NMR (400 MHz, Chloroform-*d*) *δ* 7.96 (s, 1H), 7.70 (dd, *J* = 8.4, 2.5 Hz, 1H), 7.18 (dd, *J* = 8.5 Hz, 1H), 6.04 (s, 1H), 3.76 (t, 4H), 3.39 (t, *J* = 5.9 Hz, 2H), 2.81 (s, 3H), 2.67 (s, 3H), 2.64 (t, *J* = 6.0 Hz, 2H), 2.58 (t, *J* = 5.2 Hz, 4H), 2.45 (s, 3H), 2.35 (s, 3H). ^13^C NMR (151 MHz, Chloroform-*d*) *δ* 169.41, 163.22, 158.23, 154.30, 150.19, 141.88, 132.98, 126.84, 122.00, 119.27, 95.34, 57.35, 53.00, 48.29, 43.97, 24.49, 21.86, 21.05, 13.05. HRMS: [M+H]^+^ calc. 433.2571 (C_22_H_28_N_10_); found 433.2543 (*m*/*z*).

### *N*-(4-chloro-6-methylpyrimidin-2-yl)-4,6,8-trimethylquinazolin-2-amine (6)

POCl_3_ (0.5 mL) was added to quinazoline 5 (100 mg, 0.33 mmol) and refluxed in a sealed microwave vial at 100 °C overnight. Progress of the reaction was monitored using TLC. On completion, the reaction mixture was allowed to cool and the excess of POCl_3_ was evaporated under reduced pressure. The crude was treated with ice cold water and neutralized with 1N NaHCO_3_ solution. After neutralization the reaction mixture was subjected to extraction with ethyl acetate (3 × 50 mL). The combined organic extracts were dried over anhydrous sodium sulphate and concentrated under reduced pressure to give a yellow solid powder which on washing with Et_2_O yielded compound 6 in (107 mg, quantitative). ^1^H NMR (400 MHz, Chloroform-*d*) *δ* 8.33 (s, 1H), 7.58 (s, 1H), 7.48 (s, 1H), 6.81 (s, 1H), 2.85 (s, 3H), 2.72 (s, 3H), 2.49 (s, 3H), 2.46 (s, 3H).

### *N*-(4-(4-(2-azidoethyl)piperazin-1-yl)-6-methylpyrimidin-2-yl)-4,6,8-trimethylquinazolin-2-amine (GL2)

Quinazoline 6 (100 mg, 0.32 mmol) was charged with piperazine-azide (146 mg, 0.54 mmol) in dry DMF (2 mL) followed by TEA (0.11 mL, 0.81 mmol). The reaction was heated at 80 °C for 24 h and monitored through LC-MS. On completion, reaction mixture was allowed to cool, subjected to evaporation of DMF and purified through flash column chromatography in basic alumina (eluent: 0.1% to 0.3% MeOH in DCM). Light yellow product GL2 was obtained (56 mg), 41% yield. ^1^H NMR (400 MHz, Chloroform-*d*) *δ* 7.86 (s, 1H), 7.57 (s, 1H), 7.44 (s, 1H), 6.03 (s, 1H), 3.79 (t, 4H), 3.40 (t, *J* = 6.0 Hz, 2H), 2.82 (s, 3H), 2.69 (s, 3H), 2.65 (t, 2H), 2.59 (t, 4H), 2.46 (s, 3H), 2.34 (s, 3H). ^13^C NMR (151 MHz, Chloroform-*d*) *δ* 168.99, 163.21, 158.18, 153.80, 148.80, 135.93, 135.22, 133.43, 121.87, 120.82, 95.22, 57.35, 53.00, 48.31, 43.97, 29.82, 24.43, 22.03, 21.68, 17.59. HRMS: [M+H]^+^ calc. 433.2571 (C_22_H_28_N_10_); found 433.2564 (*m*/*z*).

### *N*-(4-(4-(2-azidoethyl)piperazin-1-yl)-6-methylpyrimidin-2-yl)quinoxalin-2-amine (GL3)

An oven-dried microwave vial (2–5 mL) was charged with quinoxaline 7 (50 mg, 0.20 mmol) and PyAOP (134 mg, 0.26 mmol). Then, anhydrous DMF (1.6 mL) and DBU (44.2 µL, 0.30 mmol) were added and the resulting solution was stirred for 15 min. Piperazine-azide (79.7 mg, 0.30 mmol) was then added and the solution was stirred at ambient temperature overnight. Upon completion, DMF was evaporated, and purification was done using flash SiO_2_ column chromatography (eluent: 0.1% to 2% MeOH in DCM) afforded GL3 as a yellow solid (8 mg, 10%). ^1^H NMR (400 MHz, Chloroform-*d*) *δ* 10.04 (s, 1H), 8.01 (dd, *J* = 8.4, 1.5 Hz, 1H), 7.80 (dd, *J* = 8.5, 1.1 Hz, 1H), 7.66 (ddd, *J* = 8.4, 6.9, 1.5 Hz, 1H), 7.56 (ddd, *J* = 8.3, 6.9, 1.4 Hz, 1H), 7.31 (s, 1H), 6.06 (s, 1H), 3.69 (t, *J* = 5.1 Hz, 4H), 3.39 (t, 2H), 2.66 (t, 2H), 2.60 (t, 4H), 2.33 (s, 3H). ^13^C NMR (151 MHz, DMSO-*d*_6_) *δ* 162.53, 148.90, 140.84, 140.69, 138.01, 130.10, 128.52, 126.76, 126.49, 95.72, 56.56, 52.11, 47.04, 43.61, 40.43, 40.06, 23.76. HRMS: [M+H]^+^ calc. 391.2102 (C_19_H_22_N_10_); found 391.2068 (*m*/*z*).

### 4-hydroxypyridine-2,6-dicarboxylate (8)

To a suspension of chelidamic acid (1.5 g, 7.5 mmol) in 20 mL MeOH, 3 drops of concentrated H_2_SO_4_ was added and the reaction mixture was refluxed for 16 h. The reaction mixture was allowed to cool down, and ammonia was added to make the solution neutral. The solvent was removed and the appeared solid was washed with water to obtain white solid compound 8 (1.1 g, 70%). ^1^H NMR (400 MHz, DMSO-*d*_6_) *δ* 11.60 (s, 1H), 7.58 (s, 2H), 3.88 (s, 6H). ^13^C NMR (100 MHz, DMSO-*d*_6_) *δ* 165.88, 164.80, 149.30, 115.24, 52.59.

### Dimethyl 4-(4-bromobutoxy)pyridine-2,6-dicarboxylate (9)

To a solution of compound 8 (218 mg, 1 mmol) and K_2_CO_3_ (214 mg, 1.5 mmol) in acetone, 1,4 dibromo butane (890 mg, 4 mmol) was added and refluxed for 3 h. After the reaction is completed, it is allowed to cool down and solvent was evaporated. The reaction mixture was extracted with EtOAc, and the organic layer was dried over Na_2_SO_4_ and concentrated. The light-yellow oil compound 9 (270 mg, 76%) was used for the next reaction without further purification. ^1^H NMR (400 MHz, Chloroform-*d*) *δ* 7.80 (s, 2H), 4.19 (t, *J* = 5.7 Hz, 2H), 4.01 (s, 6H), 3.49 (t, *J* = 6.1 Hz, 2H), 2.06 (ddt, 4H). ^13^C NMR (151 MHz, Chloroform-*d*) *δ* 167.03, 165.29, 149.96, 114.68, 68.24, 53.47, 33.07, 29.25, 27.54.

### 4-(4-azidobutoxy)pyridine-2,6-dicarboxylic acid (10)

To a solution of compound 9 (225 mg, 0.65 mmol) in 5 mL DMF, NaN_3_ (55 mg, 1.3 mmol) was added and warmed at 80 °C for 6 h. The progress of the reaction is monitored through LCMS, after the reaction was completed, the solvent was removed and cold water added and the precipitate was filtered and used for next reaction. The azide was then dissolved in 5 mL of THF/H_2_O (1 : 1). 2 M LiOH (0.5 mL) was added to the reaction mixture and stirred for 3 h at room temperature. Upon completion, solvent was removed and cold water added. The mixture was acidified using 1 M HCl, the obtained product was filtered and washed with diethyl ether to obtain compound 10 as a white powder (135 mg, 74%). ^1^H NMR (400 MHz, DMSO-*d*_6_) *δ* 7.71 (s, 2H), 4.25 (t, *J* = 6.3 Hz, 2H), 3.42 (t, *J* = 13.5 Hz, 1H), 1.82 (dt, *J* = 8.3, 6.3 Hz, 2H), 1.74–1.65 (m, 2H). ^13^C NMR (151 MHz, DMSO-*d*_6_) *δ* 166.67, 165.33, 149.74, 113.61, 68.20, 50.25, 25.47, 24.85.

### 2,2′-(4-(4-azidobutoxy)pyridine-2,6-diyl)bis(4*H*-benzo[*d*][1,3]oxazin-4-one) (11)

To a RBF (25 mL) containing compound 10 (0.28 g, 0.6 mmol) in anhydrous acetonitrile (10 mL) at 0 °C was added methane sulphonyl chloride (92 µL, 1.2 mmol) followed by pyridine (96 µL, 1.2 mmol) and the reaction mixture was stirred for 15 min. Then anthranilic acid 1 (0.16 g, 1.2 mmol) was added to the reaction mixture and the stirring was continued for another 15 min at 0 °C. After that again methane sulphonyl chloride (92 µL, 1.2 mmol) followed by pyridine (96 µL, 1.2 mmol) were added and the stirring was continued for 24 hours at ambient temperature. After the completion of the reaction, the mixture was poured into cold water and stirred for 15 min. The white precipitate was filtered and refluxed acetic anhydride for 2 h. After completion of the reaction Ac_2_O was removed and the residue was recrystallized from ethanol to give compound 11 as a white powder (200 mg, 70%). ^1^H NMR (400 MHz, DMSO-*d*_6_) *δ* 8.23 (dd, *J* = 7.9, 1.5 Hz, 2H), 8.03 (s, 3H), 8.01 (td, 2H), 7.84 (dd, *J* = 8.1, 1.1 Hz, 2H), 7.72 (td, *J* = 7.6, 1.2 Hz, 2H), 4.36 (t, *J* = 6.2 Hz, 2H), 3.47 (t, *J* = 6.7 Hz, 2H), 1.89 (dd, *J* = 7.2, 4.1 Hz, 2H), 1.82–1.73 (m, 2H). ^13^C NMR (151 MHz, DMSO-*d*_6_) *δ* 166.15, 158.88, 154.77, 150.11, 145.79, 137.00, 129.52, 128.20, 127.36, 117.55, 112.75, 50.30, 25.61, 24.89.

### 2,2′-(4-(4-azidobutoxy)pyridine-2,6-diyl)bis(quinazolin-4(3*H*)-one) (12)

To a MW tube (2–5 mL) containing compound 11 (200 mg, 0.4 mmol) was added 5 mL ammonium hydroxide and the reaction mixture was refluxed for 36 hours. The progression of the reaction was monitored through LCMS. After the completion of the reaction, ammonia was removed and 5 mL water was added to the reaction mixture and was acidified to pH 5–6 by adding 1 N HCl. The white precipitate was filtered and recrystallized from methanol to give compound 12 as a white powder (108 mg, 54%). ^1^H NMR (400 MHz, DMSO-*d*_6_) *δ* 13.29 (s, 2H), 8.25 (dd, *J* = 7.9, 1.5 Hz, 2H), 8.21 (s, 2H), 7.91 (ddd, *J* = 8.4, 6.9, 1.6 Hz, 2H), 7.88–7.83 (m, 2H), 7.62 (ddt, 2H), 4.39 (t, *J* = 6.2 Hz, 2H), 3.48 (t, *J* = 6.8 Hz, 2H), 1.90 (dd, 2H), 1.79 (dd, 2H). ^13^C NMR (151 MHz, DMSO-*d*_6_) *δ* 166.74, 161.79, 150.62, 149.45, 148.22, 134.78, 127.82, 127.51, 126.13, 122.17, 110.73, 68.22, 50.31, 25.64, 24.90.

### Di-*tert*-butyl 4,4′-((4-(4-azidobutoxy)pyridine-2,6-diyl)bis(quinazoline-2,4-diyl))bis(piperazine-1-carboxylate) (13)

To a RBF (10 mL) containing compound 12 (50 mg, 0.10 mmol) and PyAOP (163 mg, 0.3 mmol) 5 mL of anhydrous DMF was added and stirred. To the stirring reaction mixture DBU (62 µL, 0.4 mmol) and 1-Boc-piperazine (40 mg, 0.2 mmol) were added and the reaction mixture was stirred for 24 h. The reaction was monitored through LCMS. After the completion of the reaction, the solvent was removed through vacuum and water was added. The precipitate was filtered was crystalized from ethanol to give compound 13 (52 mg, 61%). ^1^H NMR (400 MHz, DMSO-*d*_6_) *δ* 8.20 (t, *J* = 4.3 Hz, 4H), 8.10 (d, *J* = 8.4 Hz, 2H), 8.00 (t, *J* = 7.7 Hz, 2H), 7.69 (t, *J* = 7.7 Hz, 2H), 4.44 (t, *J* = 6.2 Hz, 2H), 4.08 (d, 8H), 3.49 (t, *J* = 6.7 Hz, 3H), 1.93 (dd, *J* = 8.9, 5.6 Hz, 2H), 1.80 (dd, *J* = 8.6, 6.0 Hz, 2H), 1.46 (s, 18H). ^13^C NMR (151 MHz, DMSO-*d*_6_) *δ* 163.37, 153.93, 134.48, 126.88, 126.31, 125.79, 114.22, 111.77, 79.30, 68.52, 50.38, 48.84, 45.87, 28.07, 25.62, 25.04.

### 4,4′-((4-(4-azidobutoxy)pyridine-2,6-diyl)bis(quinazoline-2,4-diyl))bis(piperazin-1-ium) (GL4)

To a RBF (10 mL) containing compound 13 (30 mg, 0.04 mmol), 5 mL of 10% of CF_3_CO_2_H in DCM was added and the reaction mixture was stirred at ambient temperature for 2 hours. The reaction was monitored through LCMS. After the completion of the reaction, the solvent was removed through vacuum and diethyl ether was added, sonicated and then filtered to afford GL4 (26 mg, 90%) as white powder. ^1^H NMR (400 MHz, DMSO-*d*_6_) *δ* 9.26 (s, 4H), 8.31 (d, *J* = 1.9 Hz, 2H), 8.27 (d, *J* = 8.3 Hz, 2H), 8.19 (d, *J* = 8.4 Hz, 2H), 8.07 (t, *J* = 7.7 Hz, 2H), 7.76 (t, *J* = 7.7 Hz, 2H), 4.50 (t, *J* = 6.2 Hz, 2H), 4.26 (s, 8H), 3.50 (t, *J* = 6.7 Hz, 2H), 1.94 (dd, *J* = 11.2, 6.2 Hz, 2H), 1.84–1.77 (m, 2H). ^13^C NMR (151 MHz, DMSO-*d*_6_) *δ* 169.04, 164.28, 158.69, 154.06, 135.33, 128.03, 126.79, 117.93, 116.25, 114.62, 112.61, 69.42, 50.80, 46.73, 43.06, 26.06, 25.48. HRMS: *m*/*z*: [M+H]^+^ calc for C_33_H_36_N_12_O 617.3208; found 617.3209.

### 2,2′-(4-(4-azidobutoxy)pyridine-2,6-diyl)bis(*N*-(3-(piperidin-1-yl)propyl)-3,4-dihydroquinazolin-4-amine) (GL5)

To a RBF (10 mL) containing compound 12 (50 mg, 0.10 mmol) and PyAOP (163 mg, 0.3 mmol) 5 mL of anhydrous DMF was added and stirred. To the stirring reaction mixture DBU (62 µL, 0.4 mmol) and 1-(3-Aminopropyl)piperidine (33 µL, 0.2 mmol) were added and the reaction mixture was stirred for 24 h. The reaction was monitored through LCMS. After the completion of the reaction, the solvent was removed through vacuum and water was added. The precipitate was filtered was crystalized from ethanol to give the pure product GL5 (38 mg, 50%). ^1^H NMR (400 MHz, DMSO-*d*_6_) *δ* 8.52 (s, 2H), 8.28 (d, *J* = 8.2 Hz, 2H), 8.03 (s, 2H), 7.87–7.81 (m, 4H), 7.58 (t, *J* = 7.5 Hz, 2H), 4.31 (t, *J* = 6.2 Hz, 2H), 3.72 (q, *J* = 6.3 Hz, 4H), 3.47 (t, *J* = 6.7 Hz, 4H), 3.01 (q, *J* = 6.7, 3.4 Hz, 4H), 2.02–1.88 (m, 8H), 1.77–1.71 (m, 6H), 1.52–1.45 (m, 8H), 1.34 (d, *J* = 7.4 Hz, 4H). ^13^C NMR (151 MHz, DMSO-*d*_6_) *δ* 165.57, 159.96, 159.49, 157.99, 149.70, 132.87, 128.11, 125.98, 122.67, 114.10, 110.52, 67.32, 50.39, 45.86, 45.84, 25.93, 25.88, 25.80, 25.10. HRMS: *m*/*z*: [M+H]^+^ calcd for C_41_H_52_N_12_O; 729.4460; found 729.4406.

### 2-aminoquinolin-4-ol (14)

A solution of Isatoic anhydride (2 g, 12.3 mmol) in dry DMF (10 mL) is added dropwise for 30 minutes to a warm solution (50–60 °C) of malononitrile (891 mg, 13.5 mmol) and TEA (1.9 mL, 13.5 mmol) in dry DMF (10 mL). Release of CO_2_ is observed during addition of anhydride and after addition heating is continued for another 30 minutes. The clear dark solution is poured in ice-cold 0.2 N HCL solution to get a precipitate. The product is filtered and washed with water and dried. Then the precipitate is suspended in 48% HBr and refluxed for 24 h. The progression of the reaction is monitored through LCMS. After the completion of the reaction, the reaction mixture is cooled with ice water, the precipitate is filtered and wash with ice cold water. The precipitate is dissolved in warmed water, and the solution is made alkaline with NH_4_OH. The resulting precipitate is filtered and washed with water and diethyl ether to obtain compound 14 (1.4 g, 71%). ^1^H NMR (400 MHz, DMSO-*d*_6_) *δ* 10.67 (d, *J* = 16.2 Hz, 1H), 7.90 (dt, *J* = 8.0, 2.1 Hz, 1H), 7.47–7.40 (m, 1H), 7.26 (d, *J* = 8.1 Hz, 1H), 7.11 (t, *J* = 7.5 Hz, 1H), 6.16 (d, *J* = 10.4 Hz, 2H), 5.25 (d, *J* = 9.7 Hz, 1H). ^13^C NMR (151 MHz, DMSO-*d*_6_) *δ* 174.93, 154.34, 138.68, 130.26, 124.49, 123.35, 121.37, 116.40, 89.49.

### *Tert*-butyl (2-((2-aminoquinolin-4-yl)oxy)ethyl)carbamate (15)

Quinolinone 14 (425 mg, 2.7 mmol), *N*-Boc-ethanolamine (642 mg, 4 mmol) and triphenylphosphine (1.5 g, 5.6 mmol) were dissolved in 10 mL freshly distilled THF and cooled to 0 °C. Diethyl azodicarboxylate (DEAD, 1.7 g, 4 mmol) was added dropwise under Ar atmosphere. The mixture was allowed to warm to ambient temperature and stirred for 3 days. The solvent was removed *in vacuo* and the product purified by SiO2 column chromatography (eluent: 10% MeOH in EtOAc) to obtain the title compound 15 as a white powder (360 mg, 45%). ^1^H NMR (400 MHz, DMSO-*d*_6_) *δ* 7.91 (dd, *J* = 8.1, 1.5 Hz, 1H), 7.43 (dd, *J* = 8.4, 6.7, 1.5 Hz, 1H), 7.37 (dd, *J* = 8.5, 1.3 Hz, 1H), 7.12 (t, 1H), 7.07 (dd, 1H), 6.24 (d, *J* = 4.4 Hz, 2H), 6.15 (s, 1H), 4.04 (t, *J* = 5.3 Hz, 2H), 3.44 (q, *J* = 5.5 Hz, 2H), 1.39 (s, 9H). ^13^C NMR (100 MHz, DMSO-*d*_6_) *δ* 160.91, 159.46, 155.81, 148.84, 129.41, 124.80, 121.71, 120.18, 116.75, 90.48, 77.81, 66.93, 28.23.

### Di-*tert*-butyl (((((4-(4-azidobutoxy)pyridine-2,6-dicarbonyl)bis(azanediyl))bis(quinoline-2,4-diyl))bis(oxy))bis(ethane-2,1-diyl))dicarbamate (16)

To a solution of compound 10 (32 mg, 0.1 mmol) and 15 (70 mg, 0.2 mmol) in 6 mL dry DMF, DIPEA (100 µL, 0.5 mmol) followed by HATU (130 mg, 0.3 mmol) were added. The reaction mixture was stirred under N_2_ atmosphere for 24 h. The progress of the reaction mixture was monitored by LCMS. After the completion of the reaction (slight amount 15 was unreacted) the solvent was evaporated and water was added. After sonication for few minutes, the solid was filtered and purified by SiO_2_ column chromatography (eluent: 3% MeOH in DCM), and compound 16 was obtained (52 mg, 54%). ^1^H NMR (400 MHz, DMSO-*d*_6_) *δ* 12.04 (s, 2H), 8.24 (d, *J* = 8.4 Hz, 2H), 8.05 (d, *J* = 2.3 Hz, 2H), 7.95 (s, 1H), 7.93 (s, 3H), 7.77 (ddd, *J* = 8.6, 7.1, 1.7 Hz, 2H), 7.51 (t, *J* = 7.5 Hz, 2H), 7.21 (t, *J* = 5.9 Hz, 2H), 4.35 (t, *J* = 6.3 Hz, 2H), 4.27 (t, *J* = 5.3 Hz, 4H), 3.54 (d, *J* = 5.4 Hz, 2H), 3.47 (d, *J* = 6.8 Hz, 4H), 1.92–1.85 (m, 2H), 1.78–1.71 (m, 2H), 1.40 (s, 18H). ^13^C NMR (151 MHz, DMSO-*d*_6_) *δ* 167.67, 163.73, 162.79, 156.32, 152.83, 151.48, 147.27, 131.18, 127.12, 125.00, 122.71, 119.67, 112.58, 95.39, 78.34, 68.77, 68.31, 50.76, 28.71, 25.96, 25.37.

### 2,2′-((((4-(4-azidobutoxy)pyridine-2,6-dicarbonyl)bis(azanediyl))bis(quinoline-2,4-diyl))bis(oxy))bis(ethan-1-aminium) (GL7)

To solution of compound 16 (23 mg, 0.027 mmol) in 3 mL DCM, 0.5 mL TFA was added, and the reaction mixture was stirred at room temperature for 2 h. LCMS was checked to confirm the completion of reaction. The solvent was evaporated, and diethyl ether was added to the reaction mixture and sonicated. The solid appeared was filtered and washed with diethyl ether to obtain GL7 as a light pink powder (21 mg, 89%). ^1^H NMR (400 MHz, DMSO-*d*_6_) *δ* 12.09 (s, 1H), 8.44 (d, *J* = 8.3 Hz, 1H), 8.11 (s, 2H), 7.99–7.88 (m, 2H), 7.80 (t, 1H), 7.56 (t, *J* = 7.6 Hz, 1H), 4.51 (t, *J* = 5.0 Hz, 2H), 4.35 (t, *J* = 6.3 Hz, 1H), 3.47 (t, *J* = 6.6 Hz, 3H), 1.95–1.83 (m, 1H), 1.76 (q, *J* = 7.0 Hz, 1H). ^13^C NMR (151 MHz, DMSO-*d*_6_) *δ* 167.25, 163.37, 161.71, 157.89, 152.34, 151.03, 147.04, 130.86, 126.77, 124.57, 122.72, 119.04, 112.15, 95.05, 68.34, 65.17, 50.32, 38.26, 25.53, 24.93. HRMS: [M+H]^+^ calc. 651.2786 (C_33_H_34_N_10_O_5_); found 651.2732 (*m*/*z*).

### 2-azidoethyl 4-(5,15,20-tri(pyridin-4-yl)porphyrin-10-yl)benzoate (18)

To a RBF was added compound 17 (100 mg, 0.151 mmol), EDC·HCl (57.9 mg, 0.302 mmol) and HOBt (30.6 mg, 0.227 mmol), along with DMF (4 mL). The mixture was allowed to stir at ambient temperature for 45 min. To the mixture was added a solution of 1-azido-2-bromoethane (26 mg, 0.3022 mmol) and DMAP (20,3 mg, 0.166 mmol) in DMF (3 mL). The RBF was shielded from light and was allowed to stir at room temperature for 48 h. The progress of the reaction was monitored *via* LC-MS. The solvent was removed under reduced pressure, and the crude was washed with brine and purified by flash chromatography in gradient eluent system (eluent: 3–7% MeOH in DCM). The compound 18 was afforded as a purple solid (61.8 mg, 56%). ^1^H NMR (400 MHz, Chloroform-*d*) *δ* 9.06 (d, 6H), 8.91–8.81 (m, 8H), 8.31 (d, *J* = 8.2 Hz, 2H), 8.20 (d, *J* = 8.2 Hz, 2H), 8.16 (dt, *J* = 4.3, 1.4 Hz, 6H), 3.85 (dd, *J* = 6.1, 4.5 Hz, 2H), 3.75 (dd, *J* = 6.2, 4.5 Hz, 2H), −2.89 (s, 2H).

### 4,4′-(10-(4-((2-azidoethoxy)carbonyl)phenyl)-15-(1-metheyliumyl-1λ4-pyridin-4-yl)porphyrin-5,20-diyl)bis(1-methylpyridin-1-ium) (GL8)

Compound 18 (61.8 mg, 0.085 mmol) was dissolved in DMF (4 mL). To the mixture was added MeI (257 µL, 4.13 mmol). The mixture was shielded from light and heated to 50 °C. The progress of the reaction was monitored *via* TLC. The reaction mixture was allowed to cool to room temperature and subjected to solvent removal under reduced pressure. The crude was suspended in ice-cold Et2O and sonicated. The Et_2_O was carefully decanted and GL8 was afforded as a black solid (84 mg, 88%). ^1^H NMR (600 MHz, DMSO-*d*_6_) *δ* 9.56–9.48 (m, 6H), 9.34 (t, *J* = 5.4 Hz, 1H), 9.15 (s, 4H), 9.00 (d, *J* = 6.3 Hz, 8H), 8.49 (s, 4H), 8.38 (d, *J* = 7.7 Hz, 2H), 8.34 (d, *J* = 7.7 Hz, 2H), 4.73 (d, *J* = 6.0 Hz, 9H), 3.67 (t, *J* = 5.3 Hz, 2H), 3.64 (t, *J* = 5.3 Hz, 2H), −3.03 (s, 2H). ^13^C NMR (151 MHz, DMSO-*d*_6_) *δ* 166.36, 164.90, 156.50, 156.44, 144.24, 143.14, 134.20, 134.15, 132.11, 126.13, 121.87, 115.41, 114.75, 49.97, 47.84. HRMS: [M+H]^+^ calc. 775.3490 (C_47_H_40_N_11_O_3_^+^); found 774.4245 (*m*/*z*).

### Conjugation of G4-ligands with oligonucleotide

5′-hexylamine oligonucleotide (Na^+^ salt, 100 nmol) was dissolved in 0.1 M NaHCO_3_ solution (100 µL, 1 mM). A solution of (1R,8S,9s)-bicyclo[6.1.0]non-4-yn-9-ylmethyl *N*-succinimidyl carbonate (20 equiv., 10 µL, 0.2 M) was freshly prepared and added to the oligo solution. The reaction mixture was vortexed and agitated for 24 h at ambient temperature. This was followed by a repeated precipitation of the oligo conjugate using 10% v/v of 3 M NaOAc and 4× volume EtOH and store in freezer for 30 min, followed by centrifugation for 15 min and discarding the supernatant. The precipitate pellet was resuspended in the same volume of NaOAc and EtOH and repeated the precipitation. After this, the pellet was washed with only EtOH, followed by drying the pellet by gently blowing N_2_ gas and the pellet was resuspended in 0.1 M NaHCO_3_ solution (100 µL, 1 mM). The G4-ligand (2.5 equiv., 25 µL, 10 mM in DMSO) was added to the dissolved oligo conjugate and vortexed and agitated for 24 h. The reaction mixture was dissolved in more water and DMSO and filtered before purified by RP-HPLC. Purification was done using a C18 semi-preparative column and 2 mL min^−1^ flowrate and a linear gradient of 5 to 60% solvent B (100% ACN). Solvent A was 50 mM triethylammonium acetate buffer. The conjugation products were confirmed with HRMS (ESI^−^).

### Assays

#### Microscale thermophoresis

G4 DNA consisting of the G4-forming sequence with or along with flanking sequence was purchased with 5′-labelled Cy5. The G4 DNA was first annealed in MST buffer (10 mM potassium phosphate, 100 mM KCl, 0.05% Tween 20, pH 7.4) by heating at 96 °C for 5 min and then slowly cooled to room temperature. The G4 DNA concentration was kept constant at 20 nM as the final concentration in the assay, to which GLs and GL-Os were serially diluted (1 : 1) with the highest concentration in the assay being between 20–40 µM (diluted in MST buffer). MST traces and binding constants (*K*_D_) were obtained using the Monolith analysis software. *K*_D_ values were calculated using a custom on time of 1.5 s, with the target concentration fixed at 20 nM. The binding curves were plotted and visualized in GraphPad Prism 10 and fit using a 1 : 1 binding model, except for GL6 in Fig. S1B.

#### Nuclear magnetic resonance spectroscopy titrations

G4 DNA consisting of the G4-forming sequence along with flanking sequence was annealed (see microscale thermophoresis), in NMR buffer (10 mM potassium phosphate, 3 mM KCl, pH 7.4). Deuterium oxide (20 µL) was added to a 3 mm NMR tube with G4 DNA solution (180 µL, 110 µM), to yield the final concentration 100 µM of G4 DNA. ^1^H NMR spectrum was recorded at 298 K with 512 scans on a 850 MHz AVANCE III HD spectrometer equipped with a 5 mm TCI cryoprobe. GL-O (20 µL, 1 mM in H2O) was added to the G4 DNA (1 : 1 ratio) and let to hybridize for 10 min before recording of the spectrum. The same was done with GL (2 µL,10 mM in DMSO). Data was processed in MestreNova 10.0.2.

#### Nuclear magnetic resonance spectroscopy thermal stabilization

After recording the ^1^H NMR spectra at 298 K, thermal melting experiment were done on the G4 DNA with or without GL/GL-O (1 : 1 ratio). The samples were equilibrated at temperatures ranging from 40–65 °C, every 5 °C, for 5 min before recording of the spectrum. Data was processed in MestreNova 10.0.2.

#### Taq polymerase STOP assay

The Taq polymerase stop assay was adapted from Jamroškovič *et al.*^28^ DNA templates were annealed to fluorescent labelled primers in 100 mM KCl by heating to 95 °C for 5 minutes, followed by slow cooling to room temperature. The indicated compound concentrations were added to 40 nM annealed template in 1× Taq Buffer (10 mM Tris-HCl pH 8.8, with and without 50 mM KCl, Thermo Fisher Scientific), 1.5 mM MgCl_2_, and 0.05 U µL^−1^ Taq polymerase (Thermo Fisher Scientific). Samples were preincubated on ice (10 minutes) and reactions initiated with the addition of dNTPS (100 µM) and transferring the samples to 37 °C. After 15 min at 37 °C, reactions were stopped by addition of equal volume of 2× stop solution (0.5% SDS, 25 mM EDTA, XC-Dye in Formamide) and separated on a 12% polyacrylamide Tris-Borate-EDTA (TBE) gel containing 25% formamide and 8 M urea. Fluorescent signal was detected with a Typhoon Scanner (Amersham Biosciences). The intensity of the full-length band was quantified using Image Quant TL 10.2 software (GE Healthcare Life Sciences) and compared to sample without compound.

## Author contributions

E. C. and S. W. initiated and directed the project. A. A., S. W., E. C., S. K., A. B., R. N. D, N. C., K. A. developed the project. A. A wrote the manuscript. Synthesis and oligonucleotide conjugations were done by A. A., S. K, R. N. D. Polymerase stop assay was done by A. B. and K. A. Analysis of the data was done by all authors. All authors read and edited the manuscript.

## Conflicts of interest

The authors declare no conflict of interest.

## Supplementary Material

CB-OLF-D5CB00302D-s001

## Data Availability

Detailed synthetic procedures and conjugation methods, experimental data for the assays are provided in the supplementary information (SI). supplementary information detailed experimental procedures, compound evaluations, and characterization of the compounds. See DOI: https://doi.org/10.1039/d5cb00302d.
