# Supplementary material for: Tuning potency for precision: the role of the G4-ligand in G4-ligand conjugated oligonucleotides targeting individual G-quadruplex DNA structures
Source: RSC Chem Biol. 2026 Feb 10;7(4):615–29. doi: 10.1039/d5cb00302d (PMC12915683; doi:10.1039/d5cb00302d)
Supplement: CB-007-D5CB00302D-s001 [file CB-007-D5CB00302D-s001.pdf]

Supplementary Information (SI) for RSC Chemical Biology.
This journal is © The Royal Society of Chemistry 2026

Tuning Potency for Precision: The Role of the G4-Ligand in G4-Ligand–Conjugated Oligonucleotides Targeting Individual G-Quadruplex DNA Structures

Supporting Information

Table of content

Table S1. G4 DNA templates 3
Table S2. 3
Figure S1. 4
Figure S2. 4
Figure S3. 5
Figure S4. 6
Figure S5. 7
Figure S6. 8
Figure S7. 8
Figure S8. 8
Figure S9. 9
Figure S10. 9
Table S3. 10
General Experimental 11
Synthesis 11
NMR spectra 22
Conjugation 42
Chromatograms of the GL-O conjugates 42
Table S4. 44
In vitro assays 45
References 45

Table S1. G4 DNA templates (G4-forming sequence in red).

Type of assay	Name	Sequence (5´-3´)
**NMR**	*c-MYC* Pu24T	CTG AAT TGG ACG TGA TGA GGG TGG TGA GGG TGG GGA AGG
**MST**	*c-MYC* Pu24T	[Cy5]-CTG AAT TGG ACG TGA TGA GGG TGG TGA GGG TGG GGA AGG
**Polymerase Stop assay**	*c-MYC* Pu24T	CTG AAT TGG ACG TGA TGA GGG TGG TGA GGG TGG GGA AGG CAC GTG AGT TGA GTG GAG TTG GAA GTA GGC ATA CCC CTA T
**Polymerase Stop assay**	15-nt TET primer	[TET]-GCC TAC TTC CAA CTC
**Polymerase Stop assay**	25-nt TET primer	[TET]-ATA GGG GTA TGC CTA CTT CCA ACT C
**MST**	*c-MYC* Pu24T without flanking sequence	[Cy5]-TGA GGG TGG TGA GGG TGG GGA AGG
**MST**	*c-MYC Pu27* with non-complementary flanking sequence	[Cy5]-GAG GGG CGC TTA TGG GGA GGG TGG GGA GGG TGG GGA AGG
**MST**	*Helicase B* G4-1	[Cy5]-CGA GGG GAG GGC ATG TGG AAG GGG CGG GGC AA
**MST**	*Helicase B* G4-1 with non-complementary flanking sequence	[Cy5]-GTT TCC AGG AAT TGC CGA GGG GAG GGC ATG TGG AAG GGG CGG GGC AA
**MST**	*c-Kit2*	[Cy5]-CGG GCG GGC GCT AGG GAG GGT

Table S2. GL-O conjugates synthesized in study.

GL-O	G4-ligand	Type of G4-ligand	Sequence (5´-3´)
**GL-O1**	**GL1**	Quinazoline-pyrimidine	Amine C6 - TCA CGT CCA ATT CAG
**GL-O2**	**GL2**	Quinazoline-pyrimidine	Amine C6 - TCA CGT CCA ATT CAG
**GL-O3**	**GL3**	Quinoxaline-pyrimidine	Amine C6 - TCA CGT CCA ATT CAG
**GL-O4**	**GL4**	Pyridine bis-quinazoline	Amine C6 - TCA CGT CCA ATT CAG
**GL-O5**	**GL5**	Pyridine bis-quinazoline	Amine C6 - TCA CGT CCA ATT CAG
**GL-O6**	**GL6**	PhenDC3	Amine C6 - TCA CGT CCA ATT CAG
**GL-O7**	**GL7**	Pyridostatin	Amine C6 - TCA CGT CCA ATT CAG
**GL-O8**	**GL8**	TMPyP4	Amine C6 - TCA CGT CCA ATT CAG
**GL-09**	**GL9**	Benzyl (negative control)	Amine C6 - TCA CGT CCA ATT CAG

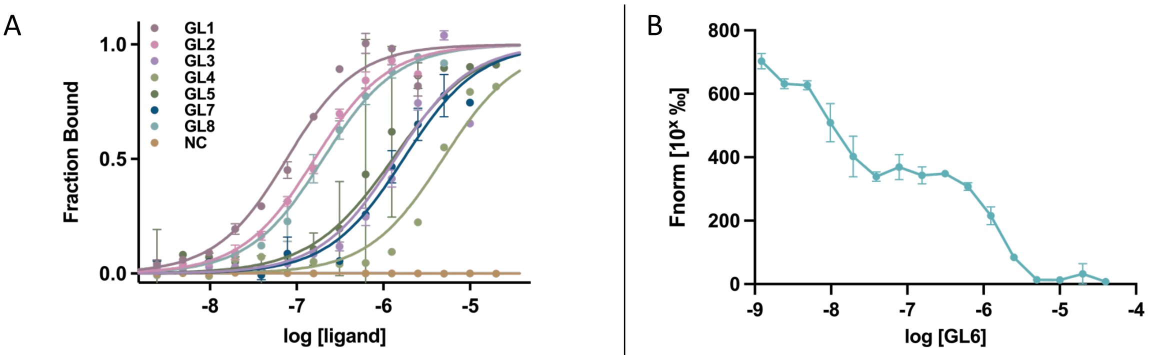

	GL1	GL2	GL3	GL4[a]	GL5[a]	GL7	GL8[a]	NC
K_D (nM)	67 ± 2	150 ± 17	1400 ± 50	4900 ± 390	1600 ± 66	1700 ± 50	190 ± 43	No binding

[a] K_D value measured using flourescence quencing and not microscale thermophoresis.

	Low K_D binding site	High K_D binding site[b]
K_D (nM)[a]	3 ± 1	1200 ± 200

[a] K_D value measured using flourescence quencing and not microscale thermophoresis. [b] Measured using Hill model giving an EC_{50} value.

Figure S1. Binding affinities of the azide-functionalized G4-ligands. A) Dose-response curves obtained using MST where the GLs **1-5,7-8** and NC (benzylazide) were serial diluted to the G4 DNA 5´-labelled with a fluorescent tag. Dissociation constants (K_D) values and error bars correspond to two independent measurements. B) Binding affinity curve of **GL6** (Phen-DC3 analogue) showing the two binding events.

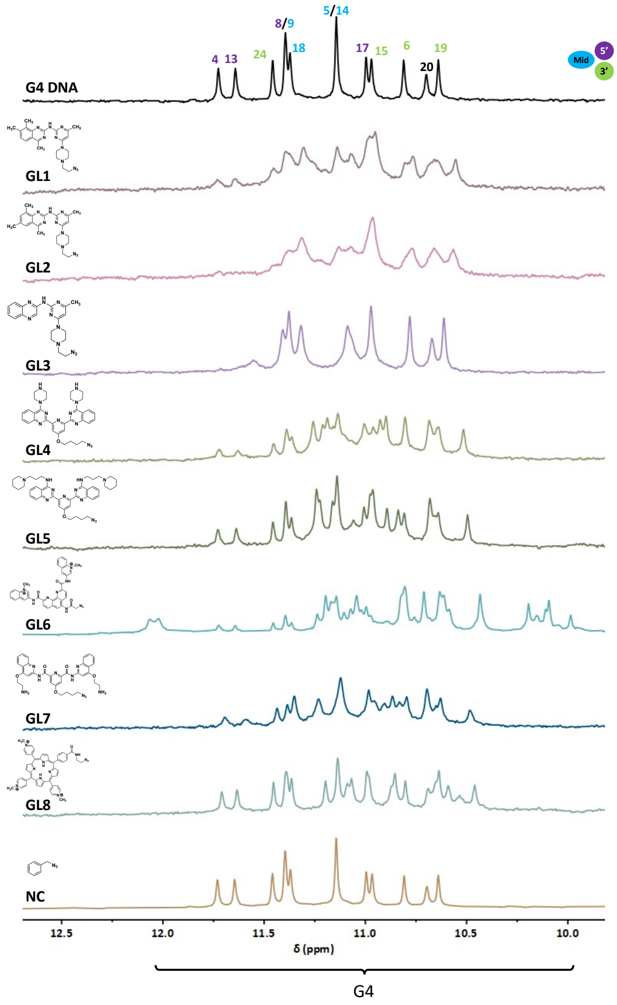

Figure S2. Binding properties of the azide-functionalized G4-ligands **1-8** and benzyl measure using ^{1}H NMR. Spectra were recorded of *c-MYC* Pu24T with complementary flanking sequence in a 1:1 molar ratio at 25 °C. G4 imino signals appear between 10-12 ppm and double-stranded DNA signals appear between 12-14 ppm.

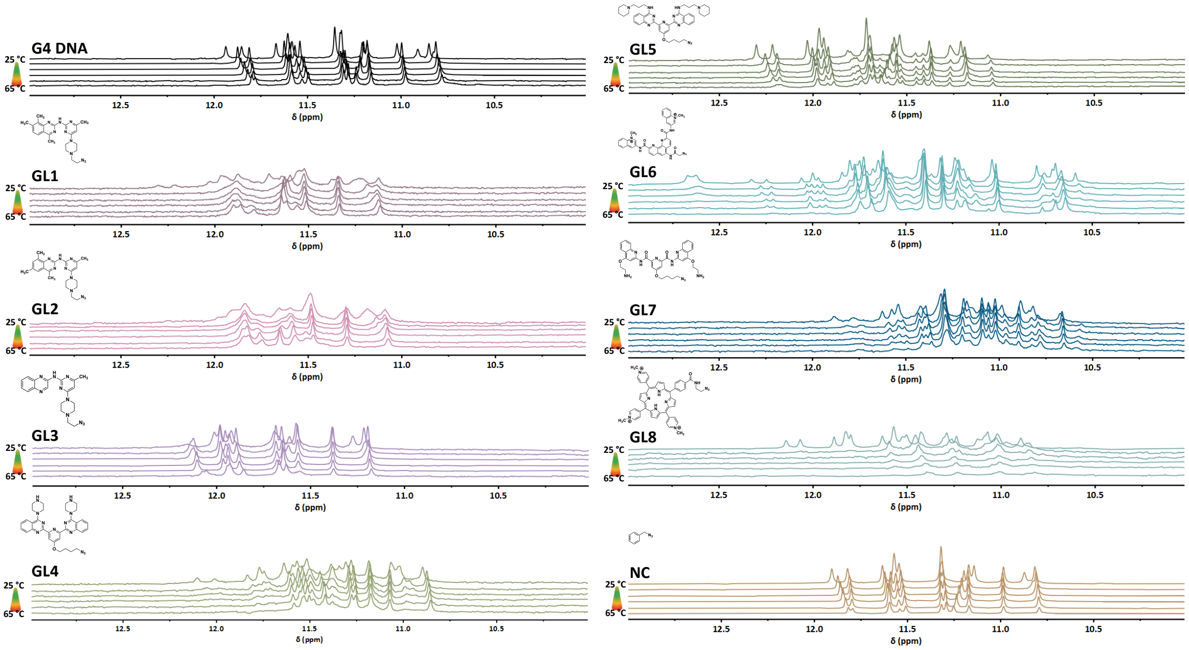

Figure S3. Thermal stabilization data of the azide-functionalized G4-ligands **1**-**8** and benzyl. Thermal melting analysis using ^{1}H NMR. Spectra were recorded of *c-MYC* Pu24T with complementary flanking with a 5 °C temperature ramp starting from 45 °C. G4 imino signals appear between 10-12 ppm and double-stranded DNA signals appear between 12-14 ppm.

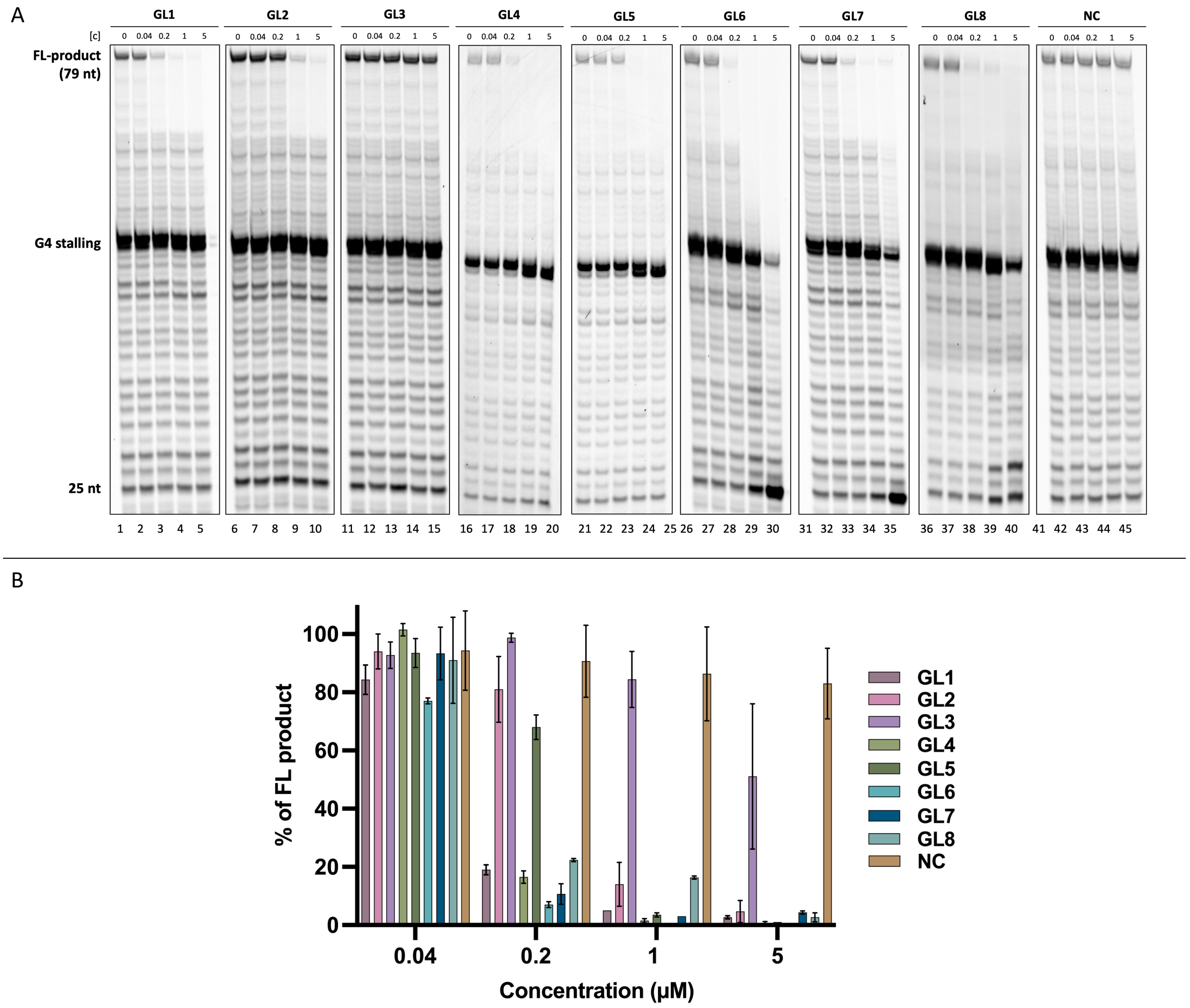

Figure S4. Polymerase stabilization of the G4 DNA in presence of the GL-Os **1**-**9**. A) A representative gel from a Taq polymerase stop assay with increasing concentration of (0.04 to 5 µM) of **GL1** (lanes 1-5), **GL2** (lanes 6–10), **GL3** (lanes 11–15), **GL4** (lanes 16–20), **GL5** (lanes 21–25), **GL6** (lanes 26–30), **GL7** (31-35), **GL8** (lanes 36-40) and **negative control** (**NC**) (lanes 41-45). B) Quantification of the Taq polymerase stop assay shown in a. The full-length (FL, 79 nt) DNA product is expressed as a % of the full-length band intensity that was observed in the control reaction containing only the G4 template. Data represent the mean ± standard deviation from three independent experiments.

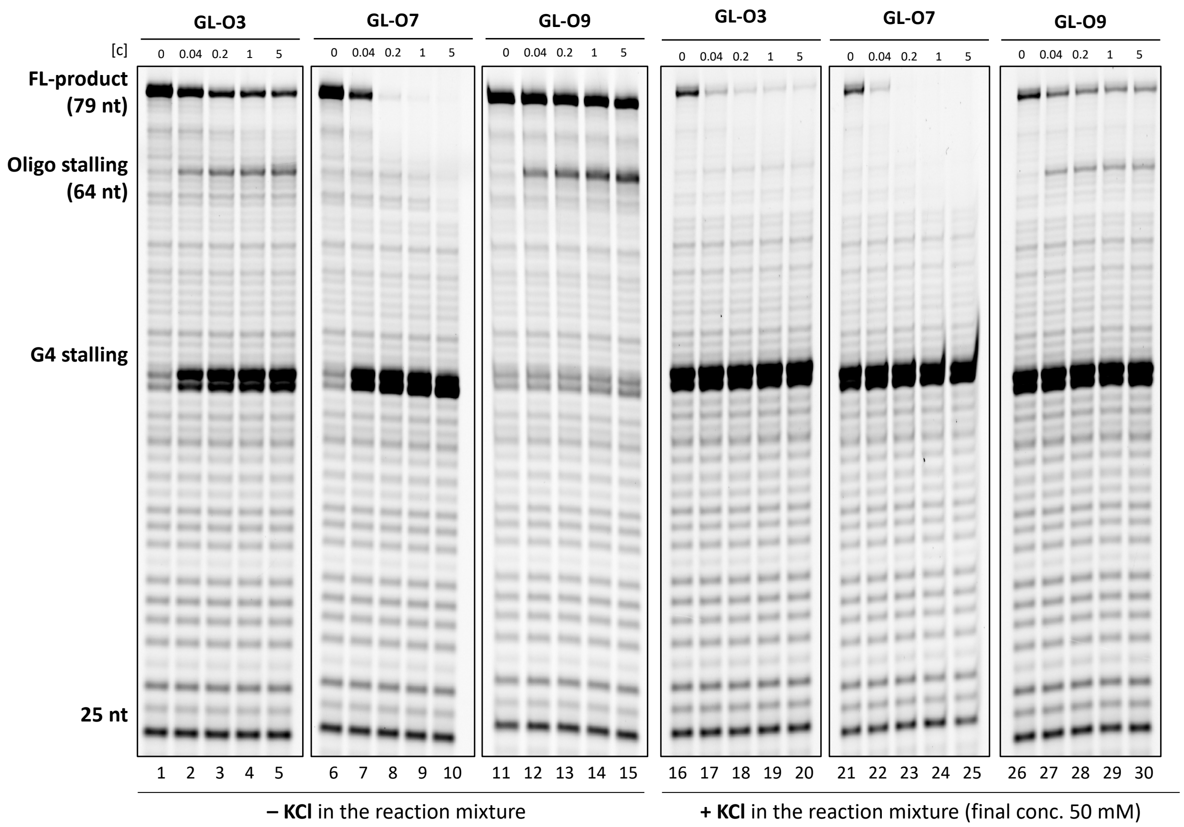

Figure S5. Polymerase stabilization of the G4 DNA in presence of the GL-Os **3**, **7** and **9** with and without addition of KCl. A representative gel from a Taq polymerase stop assay with increasing concentration of (0.04 to 5 µM) without KCl of **GL-O3** (lanes 1-5), **GL-O7** (lanes 6−10), **GL-O9** (lanes 11−15) and with KCl **GL-O3** (lanes 16−20), **GL-O7** (lanes 21−25), **GL-O9** (lanes 26−30).

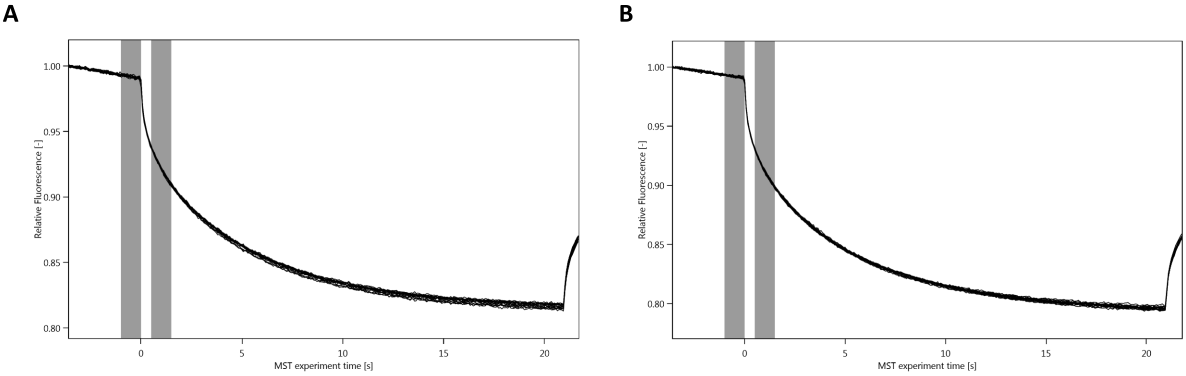

Figure S6. MST traces of binding to Pu24T G4 DNA 5´-labelled with a fluorescent tag. A) **GL-O9**. B) Unconjugated guide oligonucleotide.

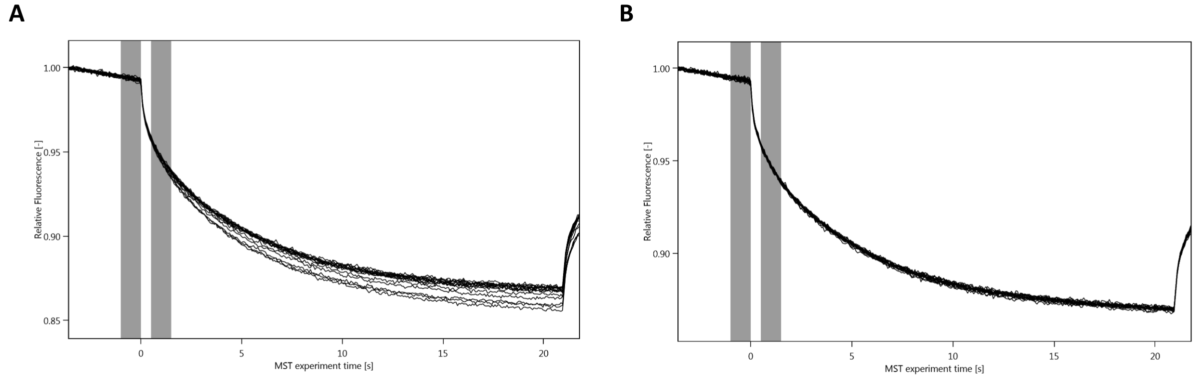

Figure S7. MST traces of binding to *c-MYC Pu27* with flanking sequence G4 DNA 5´-labelled with a fluorescent tag. A) **GL-O9**. B) Unconjugated guide oligonucleotide.

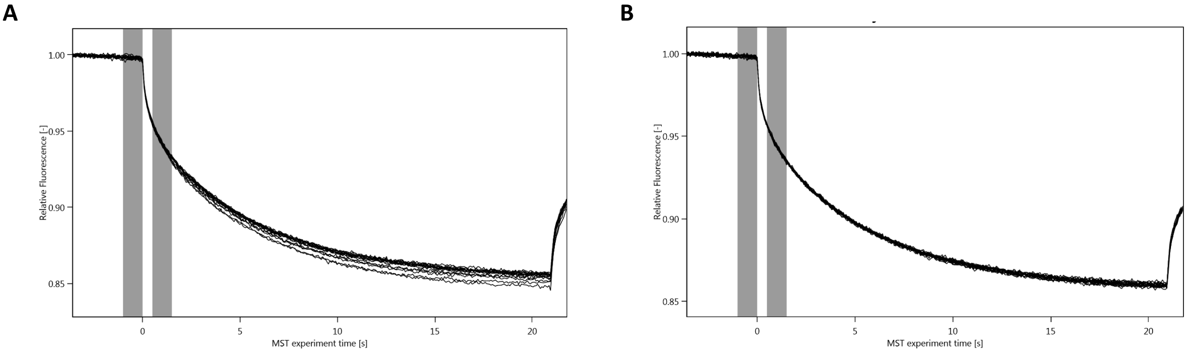

Figure S8. MST traces of binding to *Helicase B* G4-1 G4 DNA 5´-labelled with a fluorescent tag. A) **GL-O9**. B) Unconjugated guide oligonucleotide.

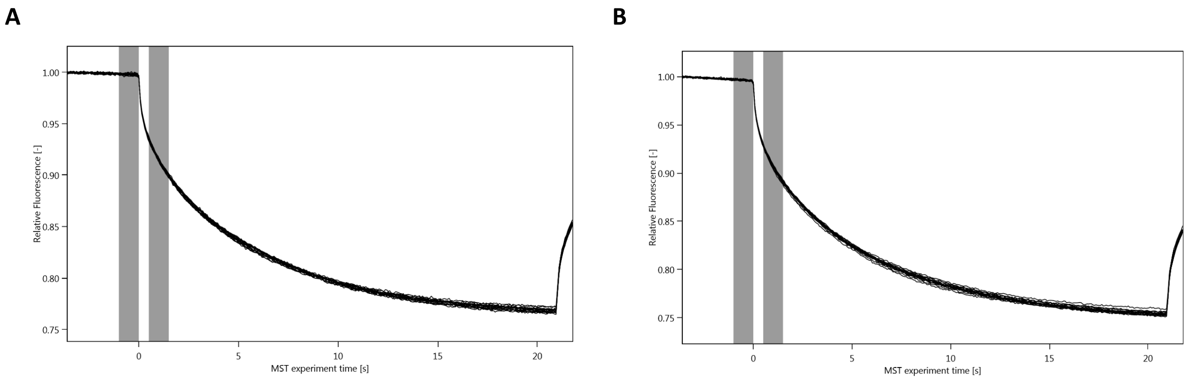

Figure S9. MST traces of binding to *Helicase B* G4-1 with flanking sequence G4 DNA 5´-labelled with a fluorescent tag. A) **GL-O9**. B) Unconjugated guide oligonucleotide.

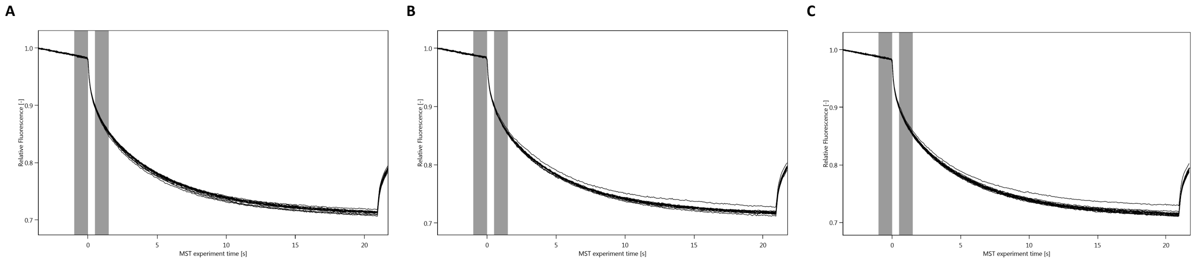

Figure S10. MST traces of binding to *c-Kit 2* G4 DNA 5´-labelled with a fluorescent tag. A. **GL-O9**. B) Unconjugated guide oligonucleotide. C) **GL-O7**.

Table S3. Binding affinity constants for four different G4 targets.

GL-O	Binding affinity constant K_D (μM)	Standard deviation (μM)
**Target: *c-MYC* Pu24T**		
**GL-O1**	N.B	N.B
**GL-O2**	N.B	N.B
**GL-O3**	N.B	N.B
**GL-O4**	N.D	N.D
**GL-O5**	6 000	1 700
**GL-O6**	3 600	500
**GL-O7**	700	200
**GL-O8**	N.D	N.D
**Target: *c-MYC* Pu27 with non-complementary flanking sequence**		
**GL-O1**	5 100	2 000
**GL-O2**	7 700	500
**GL-O3**	15 000	8 100
**GL-O4**	3 700	800
**GL-O5**	3 600	100
**GL-O6**	2 000	700
**GL-O7**	900	5
**GL-O8**	2 000	1 000
**Target: *Helicase B***		
**GL-O1**	5 200	1 700
**GL-O2**	4 000	200
**GL-O3**	N.B	N.B
**GL-O4**	N.D	N.D
**GL-O5**	3 800	500
**GL-O6**	2 300	300
**GL-O7**	1 000	80
**GL-O8**	N.B	N.B
**Target: *Helicase B* with non-complementary flanking sequence**		
**GL-O1**	N.D	N.D
**GL-O2**	N.D	N.D
**GL-O3**	N.D	N.D
**GL-O4**	4 400	700
**GL-O5**	1 900	1 400
**GL-O6**	1 600	1 000
**GL-O7**	2 200	500
**GL-O8**	1 000	30

N.B means no binding. N.D means not determine (due to a lack of saturated plateau).

General Experimental

Organic synthesis. All reagents and solvents were purchased from commercial suppliers unless stated otherwise. TLC was performed on aluminum backed silica gel plates (median pore size 60 Å, fluorescent indicator 254 nm) and detected with UV light. Flash column chromatography was performed using silica gel with an average particle diameter of 50 µm (range 40–65 µm, pore diameter 53 Å), or aluminum oxide 150 basic (63-200 µm), eluents are given in brackets. ^{1}H and ^{13}C NMR spectra for characterization were recorded on a Bruker 400 MHz spectrometer at 298 K or on a Bruker 600 MHz spectrometer at 298 K and calibrated by using the residual peak of the solvents as the internal standard ($CDCl_3$: δ H = 7.26 ppm; δ C = 77.16 ppm. DMSO-d_6: δ H = 2.50 ppm; δ C = 39.50 ppm). LC-MS was conducted on an Agilent 6150 Series Quadrupole LC/MS system. HRMS was performed by using an Agilent 1290 binary LC System connected to an Agilent 6230 Accurate-Mass TOF LC/MS (ESI$^+$); calibrated with Agilent G1969-85001 ESTOF Reference Mix containing ammonium trifluoroacetate, purine and hexakis (1H, 1H, 3H tetrafluoropropoxy) phosphazine in 90:10 ACN/H_2O.

Oligonucleotide conjugation. All reagents and solvents including N,N-diisopropylethylamine (DIPEA), ethylenediamine tetraacetate (EDTA), triethylamine (TEA), acetic acid, acetonitrile (ACN, HPLC grade), methanol and dimethyl sulfoxide (DMSO), were purchased from *Merck Sigma Aldrich*, and 1,1,1,3,3,3-hexafluoro-2-propanol (HFIP) from *Fluorochem*. The oligonucleotides were purchased from IDT (Integrated DNA Technologies, Sweden) and Eurofines Genomics (Germany). Water used for reactions and buffers were in Milli-Q purity. Reverse phase (RP) HPLC was carried out on a Hitachi HPLC system using Clarity 5 µm Oligo RP LC, fully porous Organo-silica C18 (250 x 10 mm) semi-preparative column (Phenomenex) with 2 mL/min flow rate with the method: 0-1 min 95% A, 1-15 min 95-40% A, 15-16 min 40-5% A, 16-19 min 5% A, 19-20 min 5-95% A, 20-24 min 95% A, using detection at 260 nm at room temperature. The buffers used for RP-HPLC were as follows: (A) 50 mM triethylammonium acetate (TEAA), pH 7; (B) 100% acetonitrile. TEAA (1 M) buffer was prepared by dropwise addition of glacial acetic acid (57 mL) to a cooled and stirred mixture of triethylamine (139 mL) in water (800 mL). The pH was adjusted with diluted acetic acid to ~7 and the volume was adjusted to 1 L with water. Characterization was done using a HRMS electrospray time-of-flight (ES-TOF) Agilent instrument. Oligonucleotides were eluted using an aqueous mixture of hexafluoroisopropanol and triethylamine with increasing gradient of methanol.

Synthesis

Piperazine-azide side chain

The synthesis has been described in Berner. A, et al. *J Am Chem Sci*. **2024**.[1]

GL1

Preparation of 2,2,4,7,8-pentamethyl-1,2-dihydroquinoline (1)

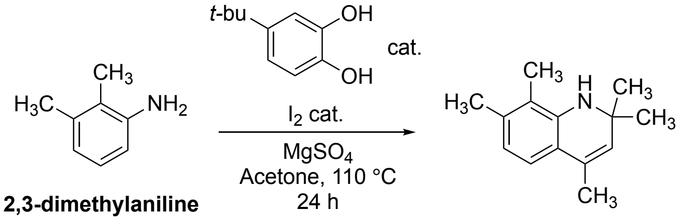

1

A microwave vial was charged with anhydrous $MgSO_4$ (3.03 g, 25.2 mmol), 4-tert-butylcatechol (24.9 mg, 0.15 mmol), and I_2 (63.45 mg, 0.25 mmol) subsequently. Then, 2,3-dimethylaniline (0.62 mL, 5.00 mmol) and acetone (13 mL) were added, and the mixture was heated to 110 °C in a sealed tube and let stir for 24 h. After cooling down, the mixture was filtered over a plug of celite with EtOAc and the

resulting organic solution was concentrated under reduced pressure. Purification by flash SiO_2 column chromatography (eluent: 2.5% EtOAc in *n*-heptane) afforded compound **1** as a red-brown oil (0.87 g, 86%). ^{1}H NMR (400 MHz, Chloroform-*d*) δ 6.89 (d, *J* = 7.7 Hz, 1H), 6.50 (d, *J* = 7.8 Hz, 1H), 5.28 (d, *J* = 1.5 Hz, 1H), 3.63 (s, 1H), 2.25 (s, 3H), 2.02 (s, 3H), 1.99 (d, *J* = 1.4 Hz, 3H), 1.29 (s, 6H). ^{13}C NMR (100 MHz, Chloroform-*d*) δ 141.18, 136.39, 129.05, 127.08, 121.03, 119.27, 118.53, 118.04, 51.93, 31.47, 20.86, 18.92, 12.37.

Preparation of 1-(4,7,8-trimethylquinazolin-2-yl)guanidine (2)

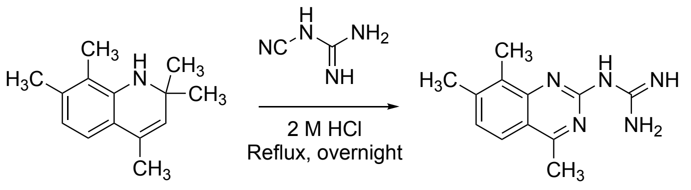

1 2

Quinoline **1** (1.81 g, 8.99 mmol) was mixed with 2-cyanoguanidine (1.52 g, 18.1 mmol) in a RBF (25 mL). Then, 2 M HCl (5 mL) was added, and the mixture was stirred at 110 °C overnight. The mixture was then allowed to cool to ambient temperature and basified with NaOH (15%, 2.9 mL) and further diluted with water. The mixture was then sonicated, and the formed precipitate was then collected with suction filtration and washed with more water and Et_2O to afford compound **2** as a white solid (1.67 g, 82%). ^{1}H NMR (400 MHz, DMSO-d_6) δ 8.39 (s, 4H), 7.91 (d, *J* = 8.5 Hz, 1H), 7.36 (d, *J* = 8.4 Hz, 1H), 2.46 (s, 6H). ^{13}C NMR (100 MHz, DMSO-d_6) δ 170.71, 156.71, 147.80, 142.70, 130.98, 127.67, 122.83, 118.70, 21.52, 20.58, 12.94.

Preparation of 6-methyl-2-((4,7,8-trimethylquinazolin-2-yl)amino)pyrimidin-4(3H)-one (3)

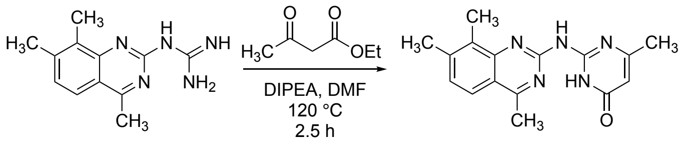

2 3

A RBF (50 mL) was charged with compound **2** (1.53 g, 6.66 mmol), followed by DMF (12.2 mL), DIPEA (2.32 mL, 13.3 mmol) and ethyl acetoacetate (8.50 mL, 67.2 mmol) sequentially. The solution was stirred at 120 °C for 2.5 h. The mixture was then diluted with Et_2O and sonicated. The precipitate was collected with suction filtration and washed with more Et_2O to afford desired compound **3** as a pale beige solid (1.13 g, 57%). ^{1}H NMR (400 MHz, DMSO-d_6) δ 13.64 (s, 1H), 11.28 (s, 1H), 7.95 (d, *J* = 8.3 Hz, 1H), 7.39 (d, *J* = 8.4 Hz, 1H), 5.82 (s, 1H), 2.84 (s, 3H), 2.53 (s, 3H), 2.47 (s, 3H), 2.17 (s, 3H).

Preparation of *N*-(4-chloro-6-methylpyrimidin-2-yl)-4,7,8-trimethylquinazolin-2-amine (4)

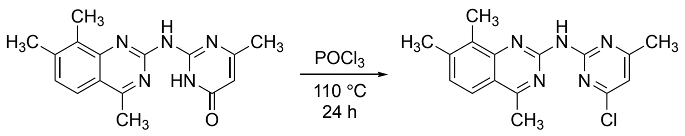

3 4

$POCl_3$ (2.5 mL) was added to quinazoline **3** (0.50 g, 1.69 mmol) and refluxed in a sealed microwave vial at 100 °C for 24 h. Progress of the reaction was monitored using TLC. On completion, the reaction mixture was allowed to cool and the excess of $POCl_3$ was evaporated under reduced pressure. The crude was treated with ice cold water and neutralized with 1N $NaHCO_3$ solution. After neutralization

the reaction mixture was subjected to extraction with ethyl acetate (3x50 mL). The combined organic extracts were dried over anhydrous sodium sulphate and concentrated under reduced pressure to give a yellow solid powder which on washing with Et_2O yielded compound **4** (0.23 g, 44%). ^{1}H NMR (400 MHz, Chloroform-*d*) δ 8.15 (s, 1H), 7.74 (d, *J* = 8.4 Hz, 1H), 7.25 (d, *J* = 8.2 Hz, 1H), 6.83 (s, 1H), 2.85 (s, 3H), 2.72 (s, 3H), 2.51 (s, 3H), 2.48 (s, 3H).

Preparation of *N*-(4-(4-(2-azidoethyl)piperazin-1-yl)-6-methylpyrimidin-2-yl)-4,7,8-trimethylquinazolin-2-amine (GL1)

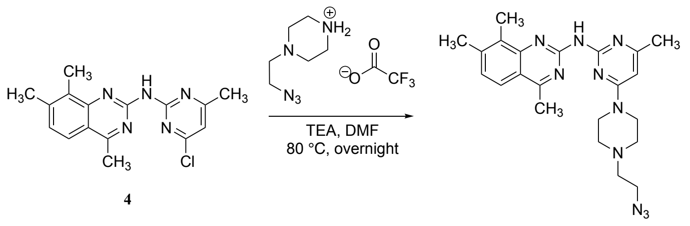

4

GL1

Quinazoline **4** (100 mg, 0.32 mmol) was charged with piperazine-azide (133 mg, 0.49 mmol) in dry DMF (2.3 mL) followed by TEA (0.11 mL, 0.81 mmol). The reaction was heated at 80 °C overnight and monitored through LC-MS. On completion, reaction mixture was allowed to cool, subjected to evaporation of DMF and purified through flash column chromatography in basic alumina (eluent: 0.1% to 0.3% MeOH in DCM). Light yellow product **GL1** was obtained (68 mg, 50% yield). ^{1}H NMR (400 MHz, Chloroform-*d*) δ 7.96 (s, 1H), 7.70 (dd, *J* = 8.4, 2.5 Hz, 1H), 7.18 (dd, *J* = 8.5 Hz, 1H), 6.04 (s, 1H), 3.76 (t, 4H), 3.39 (t, *J* = 5.9 Hz, 2H), 2.81 (s, 3H), 2.67 (s, 3H), 2.64 (t, *J* = 6.0 Hz, 2H), 2.58 (t, *J* = 5.2 Hz, 4H), 2.45 (s, 3H), 2.35 (s, 3H). ^{13}C NMR (151 MHz, Chloroform-*d*) δ 169.41, 163.22, 158.23, 154.30, 150.19, 141.88, 132.98, 126.84, 122.00, 119.27, 95.34, 57.35, 53.00, 48.29, 43.97, 24.49, 21.86, 21.05, 13.05. HRMS: $[M+H]^+$ calc. 433.2571 ($C_{22}H_{28}N_{10}$); found 433.2543 (*m/z*).

GL2

The synthesis of precursor **5** has been described in Andreasson. M, et al. *J Med Chem*. **2024.**[2]

Preparation of *N*-(4-chloro-6-methylpyrimidin-2-yl)-4,6,8-trimethylquinazolin-2-amine (6)

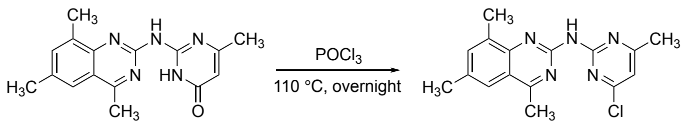

5

6

$POCl_3$ (0.5 mL) was added to quinazoline **5** (100 mg, 0.33 mmol) and refluxed in a sealed microwave vial at 100 °C overnight. Progress of the reaction was monitored using TLC. On completion, the reaction mixture was allowed to cool and the excess of $POCl_3$ was evaporated under reduced pressure. The crude was treated with ice cold water and neutralized with 1N $NaHCO_3$ solution. After neutralization the reaction mixture was subjected to extraction with ethyl acetate (3x50 mL). The combined organic extracts were dried over anhydrous sodium sulphate and concentrated under reduced pressure to give a yellow solid powder which on washing with Et_2O yielded compound **6** in (107 mg, quantitative). ^{1}H NMR (400 MHz, Chloroform-*d*) δ 8.33 (s, 1H), 7.58 (s, 1H), 7.48 (s, 1H), 6.81 (s, 1H), 2.85 (s, 3H), 2.72 (s, 3H), 2.49 (s, 3H), 2.46 (s, 3H).

Preparation of *N*-(4-(4-(2-azidoethyl)piperazin-1-yl)-6-methylpyrimidin-2-yl)-4,6,8-trimethylquinazolin-2-amine (GL2)

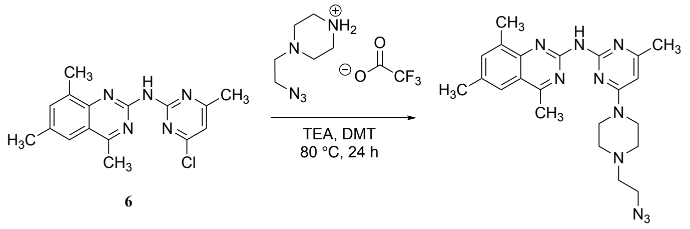

6

GL2

Quinazoline **6** (100 mg, 0.32 mmol) was charged with piperazine-azide (146 mg, 0.54 mmol) in dry DMF (2 mL) followed by TEA (0.11 mL, 0.81 mmol). The reaction was heated at 80 °C for 24 h and monitored through LC-MS. On completion, reaction mixture was allowed to cool, subjected to evaporation of DMF and purified through flash column chromatography in basic alumina (eluent: 0.1% to 0.3% MeOH in DCM). Light yellow product **GL2** was obtained (56 mg, 41% yield). ^{1}H NMR (400 MHz, Chloroform-*d*) δ 7.86 (s, 1H), 7.57 (s, 1H), 7.44 (s, 1H), 6.03 (s, 1H), 3.79 (t, 4H), 3.40 (t, *J* = 6.0 Hz, 2H), 2.82 (s, 3H), 2.69 (s, 3H), 2.65 (t, 2H), 2.59 (t, 4H), 2.46 (s, 3H), 2.34 (s, 3H). ^{13}C NMR (151 MHz, Chloroform-*d*) δ 168.99, 163.21, 158.18, 153.80, 148.80, 135.93, 135.22, 133.43, 121.87, 120.82, 95.22, 57.35, 53.00, 48.31, 43.97, 29.82, 24.43, 22.03, 21.68, 17.59. HRMS: $[M+H]^+$ calc. 433.2571 ($C_{22}H_{28}N_{10}$); found 433.2564 (*m/z*).

GL3

The synthesis of the precursor **7** has been described in Andreasson. M, et al. *J Med Chem*. **2024.**[2]

Preparation of *N*-(4-(4-(2-azidoethyl)piperazin-1-yl)-6-methylpyrimidin-2-yl)quinoxalin-2-amine (GL3)

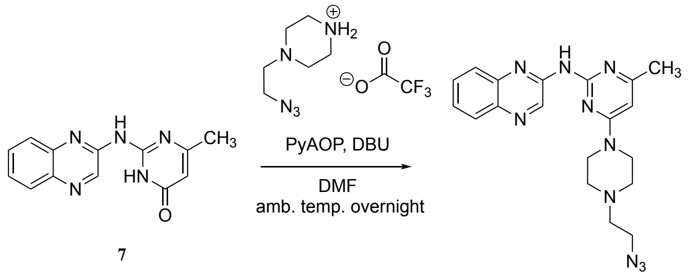

7

GL3

An oven-dried microwave vial (2-5 mL) was charged with quinoxaline **7** (50 mg, 0.20 mmol) and PyAOP (134 mg, 0.26 mmol). Then, anhydrous DMF (1.6 mL) and DBU (44.2 µL, 0.30 mmol) were added and the resulting solution was stirred for 15 min. Piperazine-azide (79.7 mg, 0.30 mmol) was then added and the solution was stirred at ambient temperature overnight. Upon completion, DMF was evaporated, and purification was done using flash SiO_2 column chromatography (eluent: 0.1% to 2% MeOH in DCM) afforded **GL3** as a yellow solid (8 mg, 10%). ^{1}H NMR (400 MHz, Chloroform-*d*) δ 10.04 (s, 1H), 8.01 (dd, *J* = 8.4, 1.5 Hz, 1H), 7.80 (dd, *J* = 8.5, 1.1 Hz, 1H), 7.66 (ddd, *J* = 8.4, 6.9, 1.5 Hz, 1H), 7.56 (ddd, *J* = 8.3, 6.9, 1.4 Hz, 1H), 7.31 (s, 1H), 6.06 (s, 1H), 3.69 (t, *J* = 5.1 Hz, 4H), 3.39 (t, 2H), 2.66 (t, 2H), 2.60 (t, 4H), 2.33 (s, 3H). ^{13}C NMR (151 MHz, DMSO-d_6) δ 162.53, 148.90, 140.84, 140.69, 138.01, 130.10, 128.52, 126.76, 126.49, 95.72, 56.56, 52.11, 47.04, 43.61, 40.43, 40.06, 23.76. HRMS: $[M+H]^+$ calc. 391.2102 ($C_{19}H_{22}N_{10}$); found 391.2068 (*m/z*).

GL4

Preparation of dimethyl 4-hydroxypyridine-2,6-dicarboxylate (8)

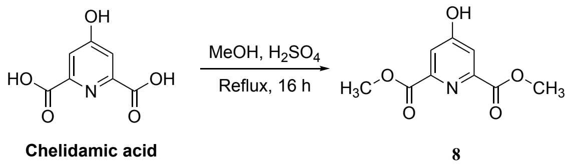

To a suspension of chelidamic acid (1.5 g, 7.5 mmol) in 20 mL MeOH, 3 drops of concentrated H_2SO_4 was added and the reaction mixture was refluxed for 16 h. The reaction mixture was allowed to cool down and ammonia was added to make the solution neutral. The solvent was removed and the appeared solid was washed with water to obtain white solid compound **8** (1.1 g, 70 %). ^{1}H NMR (400 MHz, DMSO-d_6) δ 11.60 (s, 1H), 7.58 (s, 2H), 3.88 (s, 6H). ^{13}C NMR (100 MHz, DMSO-d_6) δ 165.88, 164.80, 149.30, 115.24, 52.59.

Preparation of dimethyl 4-(4-bromobutoxy)pyridine-2,6-dicarboxylate (9)

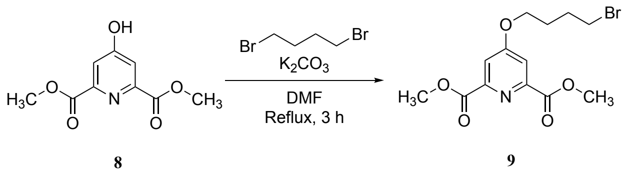

To a solution of compound **8** (218 mg, 1 mmol) and K_2CO_3 (214 mg, 1.5 mmol) in DMF, 1,4 dibromo butane (890 mg, 4 mmol) was added and refluxed for 3 h. After the reaction is completed it is allowed to cool down and solvent was evaporated. The reaction mixture was extracted with EtOAc and the organic layer was dried over Na_2SO_4 and concentrated. The light yellow oil compound **9** (270 mg, 76 %) was used for the next reaction without further purification. ^{1}H NMR (400 MHz, Chloroform-*d*) δ 7.80 (s, 2H), 4.19 (t, *J* = 5.7 Hz, 2H), 4.01 (s, 6H), 3.49 (t, *J* = 6.1 Hz, 2H), 2.06 (ddt, 4H). ^{13}C NMR (151 MHz, Chloroform-*d*) δ 167.03, 165.29, 149.96, 114.68, 68.24, 53.47, 33.07, 29.25, 27.54.

Preparation of 4-(4-azidobutoxy)pyridine-2,6-dicarboxylic acid (10)

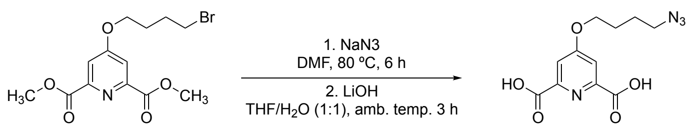

To a solution of compound **9** (225 mg, 0.65 mmol) in 5 mL DMF, NaN_3 (55 mg, 1.3 mmol) was added and warmed at 80 °C for 6 h. The progress of the reaction was monitored through LCMS, after the reaction was completed, the solvent was removed and cold water added and the precipitate was filtered and used for next reaction. The azide was then dissolved in 5 mL of THF/H_2O (1:1). 2 M LiOH (0.5 mL) was added to the reaction mixture and stirred for 3 h at room temperature. Upon completion, solvent was removed and cold water added. The mixture was acidified using 1 M HCl, the obtained product was filtered and washed with diethyl ether to obtain compound **10** as a white powder (135 mg, 74 %). ^{1}H NMR (400 MHz, DMSO-d_6) δ 7.71 (s, 2H), 4.25 (t, *J* = 6.3 Hz, 2H), 3.42 (t, *J* = 13.5 Hz, 1H), 1.82 (dt, *J* = 8.3, 6.3 Hz, 2H), 1.74 – 1.65 (m, 2H). ^{13}C NMR (151 MHz, DMSO-d_6) δ 166.67, 165.33, 149.74, 113.61, 68.20, 50.25, 25.47, 24.85.

Preparation of 2,2'-(4-(4-azidobutoxy)pyridine-2,6-diyl)bis(4H-benzo[d][1,3]oxazin-4-one) (11)

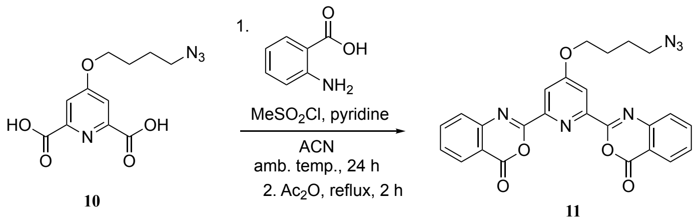

To a RBF (25 mL) containing compound **10** (0.28 g, 0.6 mmol) in anhydrous acetonitrile (10 mL) at 0 ℃ was added methane sulphonyl chloride (92 µL, 1.2 mmol) followed by pyridine (96 µL, 1.2 mmol) and the reaction mixture was stirred for 15 mins. Then anthranilic acid 1 (0.16 g, 1.2 mmol) was added to the reaction mixture and the stirring was continued for another 15 mins at 0 ℃. After that again methane sulphonyl chloride (92 µL, 1.2 mmol) followed by pyridine (96 µL, 1.2 mmol) were added again and the stirring was continued for 24 hours at ambient temperature. After the completion of the reaction, the mixture was poured into cold water and stirred for 15 mins. The white precipitate was filtered and refluxed in acetic anhydride for 2 hr. After completion of the reaction Ac_2O was removed and the residue was recrystallized from ethanol to give compound **11** as a white powder (200 mg, 70 %). ^{1}H NMR (400 MHz, DMSO-d_6) δ 8.23 (dd, J = 7.9, 1.5 Hz, 2H), 8.03 (s, 3H), 8.01 (td, 2H), 7.84 (dd, J = 8.1, 1.1 Hz, 2H), 7.72 (td, J = 7.6, 1.2 Hz, 2H), 4.36 (t, J = 6.2 Hz, 2H), 3.47 (t, J = 6.7 Hz, 2H), 1.89 (dd, J = 7.2, 4.1 Hz, 2H), 1.82 – 1.73 (m, 2H). ^{13}C NMR (151 MHz, DMSO-d_6) δ 166.15, 158.88, 154.77, 150.11, 145.79, 137.00, 129.52, 128.20, 127.36, 117.55, 112.75, 50.30, 25.61, 24.89.

Preparation of 2,2'-(4-(4-azidobutoxy)pyridine-2,6-diyl)bis(quinazolin-4(3H)-one) (12)

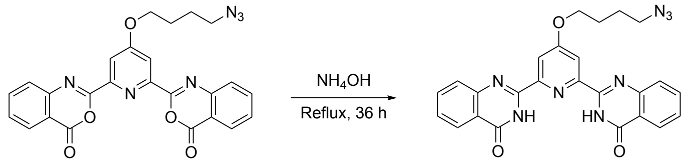

To a MW tube (2-5 mL) containing compound **11** (200 mg, 0.4 mmol) was added 5 mL ammonium hydroxide and the reaction mixture was refluxed for 36 hours. The progression of the reaction was monitored through LCMS. After the completion of the reaction, ammonia was removed and 5 mL water was added to the reaction mixture and the mixture was acidified to pH 5-6 by adding 1 N HCl. The white precipitate was filtered and recrystallized from methanol to give compound **12** as a white powder (108 mg, 54%). ^{1}H NMR (400 MHz, DMSO-d_6) δ 13.29 (s, 2H), 8.25 (dd, J = 7.9, 1.5 Hz, 2H), 8.21 (s, 2H), 7.91 (ddd, J = 8.4, 6.9, 1.6 Hz, 2H), 7.88 – 7.83 (m, 2H), 7.62 (ddt, 2H), 4.39 (t, J = 6.2 Hz, 2H), 3.48 (t, J = 6.8 Hz, 2H), 1.90 (dd, 2H), 1.79 (dd, 2H). ^{13}C NMR (151 MHz, DMSO-d_6) δ 166.74, 161.79, 150.62, 149.45, 148.22, 134.78, 127.82, 127.51, 126.13, 122.17, 110.73, 68.22, 50.31, 25.64, 24.90.

Preparation of di-tert-butyl 4,4'-((4-(4-azidobutoxy)pyridine-2,6-diyl)bis(quinazoline-2,4-diyl))bis(piperazine-1-carboxylate) (13)

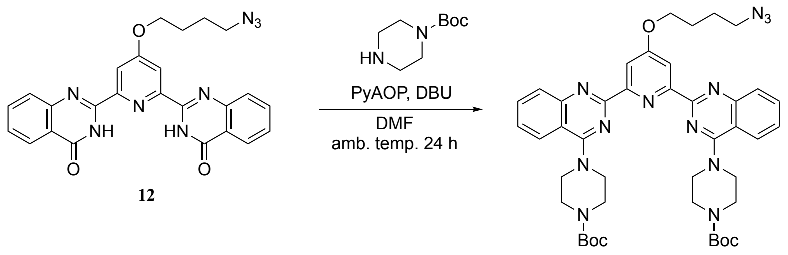

To a RBF (10 mL) containing compound **12** (50 mg, 0.10 mmol) and PyAOP (163 mg, 0.3 mmol) 5 mL of anhydrous DMF was added and stirred. To the stirring reaction mixture DBU (62 µL, 0.4 mmol) and 1-Boc-piperazine (40 mg, 0.2 mmol) were added and the reaction mixture was stirred for 24 h. The reaction was monitored through LCMS. After the completion of the reaction, the solvent was removed through vacuum and water was added. The precipitate was filtered was crystalized from ethanol to give compound **13** (52 mg, 61 %). ^{1}H NMR (400 MHz, DMSO-d_6) δ 8.20 (t, *J* = 4.3 Hz, 4H), 8.10 (d, *J* = 8.4 Hz, 2H), 8.00 (t, *J* = 7.7 Hz, 2H), 7.69 (t, *J* = 7.7 Hz, 2H), 4.44 (t, *J* = 6.2 Hz, 2H), 4.08 (d, 8H), 3.49 (t, *J* = 6.7 Hz, 3H), 1.93 (dd, *J* = 8.9, 5.6 Hz, 2H), 1.80 (dd, *J* = 8.6, 6.0 Hz, 2H), 1.46 (s, 18H). ^{13}C NMR (151 MHz, DMSO-d_6) δ 163.37, 153.93, 134.48, 126.88, 126.31, 125.79, 114.22, 111.77, 79.30, 68.52, 50.38, 48.84, 45.87, 28.07, 25.62, 25.04.

Preparation of 4,4'-((4-(4-azidobutoxy)pyridine-2,6-diyl)bis(quinazoline-2,4-diyl))bis(piperazin-1-ium) (GL4)

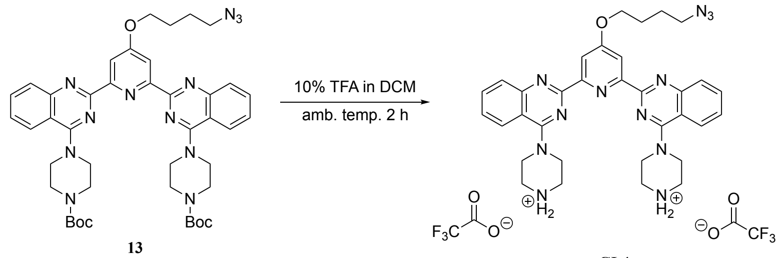

To a RBF (10 mL) containing compound **13** (30 mg, 0.04 mmol), 5 mL of 10 % of TFA in DCM was added and the reaction mixture was stirred at ambient temperature for 2 h. The reaction was monitored through LCMS. After the completion of the reaction, the solvent was removed through vacuum and diethyl ether was added, sonicated and then filtered to afford **GL4** (26 mg, 90%) as white powder. ^{1}H NMR (400 MHz, DMSO) δ 9.26 (s, 4H), 8.31 (d, *J* = 1.9 Hz, 2H), 8.27 (d, *J* = 8.3 Hz, 2H), 8.19 (d, *J* = 8.4 Hz, 2H), 8.07 (t, *J* = 7.7 Hz, 2H), 7.76 (t, *J* = 7.7 Hz, 2H), 4.50 (t, *J* = 6.2 Hz, 2H), 4.26 (s, 8H), 3.50 (t, *J* = 6.7 Hz, 2H), 1.94 (dd, *J* = 11.2, 6.2 Hz, 2H), 1.84 – 1.77 (m, 2H).^{13}C NMR (151 MHz, DMSO) δ 169.04, 164.28, 158.69, 154.06, 135.33, 128.03, 126.79, 117.93, 116.25, 114.62, 112.61, 69.42, 50.80, 46.73, 43.06, 26.06, 25.48. HRMS: m/z: $[M+H]^+$ calc for $C_{33}H_{36}N_{12}O$ 617.3208; found 617.3209.

GL5

Preparation of 2,2'-(4-(4-azidobutoxy)pyridine-2,6-diyl)bis(N-(3-(piperidin-1-yl)propyl)-3,4-dihydroquinazolin-4-amine) (GL5)

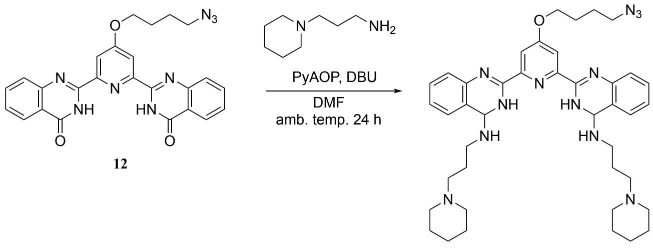

12

GL5

To a RBF (10 mL) containing compound **12** (50 mg, 0.10 mmol) and PyAOP (163 mg, 0.3 mmol) 5 mL of anhydrous DMF was added and stirred. To the stirring reaction mixture DBU (62 µL, 0.4 mmol) and 1-(3-Aminopropyl)piperidine (33 µL, 0.2 mmol) were added and the reaction mixture was stirred for 24 h. The reaction was monitored through LCMS. After the completion of the reaction, the solvent was removed through vacuum and water was added. The precipitate was filtered was crystalized from ethanol to give the pure product **GL5** (38 mg, 50%). ^{1}H NMR (400 MHz, DMSO-d_6) δ 8.52 (s, 2H), 8.28 (d, *J* = 8.2 Hz, 2H), 8.03 (s, 2H), 7.87 – 7.81 (m, 4H), 7.58 (t, *J* = 7.5 Hz, 2H), 4.31 (t, *J* = 6.2 Hz, 2H), 3.72 (q, *J* = 6.3 Hz, 4H), 3.47 (t, *J* = 6.7 Hz, 4H), 3.01 (q, *J* = 6.7, 3.4 Hz, 4H), 2.02 – 1.88 (m, 8H), 1.77 – 1.71 (m, 6H), 1.52 – 1.45 (m, 8H), 1.34 (d, *J* = 7.4 Hz, 4H). ^{13}C NMR (151 MHz, DMSO-d_6) δ 165.57, 159.96, 159.49, 157.99, 149.70, 132.87, 128.11, 125.98, 122.67, 114.10, 110.52, 67.32, 50.39, 45.86, 45.84, 25.93, 25.88, 25.80, 25.10. HRMS: m/z: $[M+H]^+$ calcd for $C_{41}H_{52}N_{12}O$; 729.4460; found 729.4406.

GL6

The synthesis of **GL6** has been described in Bagineni. P, et al. *Chem. Sci.* **2022.**[3]

GL7

Preparation of 2-aminoquinolin-4-ol (14)

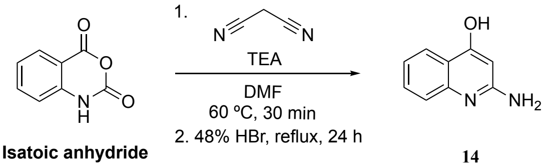

Isatoic anhydride

14

A solution of Isatoic anhydride (2 g, 12.3 mmol) in dry DMF (10 mL) was added dropwise for 30 minutes, to a warm solution (50-60 °C) of malononitrile (891 mg, 13.5 mmol) and TEA (1.9 mL, 13.5 mmol) in dry DMF (10 mL). Release of CO_2 was observed during addition of anhydride and after addition heating was continued for another 30 minutes. The clear dark solution was poured in ice-cold 0.2 N HCL solution to get a precipitate. The product was filtered and washed with water and dried. Then the precipitate was suspended in 48 % HBr and refluxed for 24 h. The progression of the reaction was monitored through LCMS. After the completion of the reaction, the reaction mixture was cooled with ice water, the precipitate was filtered and washed with ice cold water. The precipitate was dissolved in warmed water and the solution was made alkaline with NH_4OH. The resulting precipitate was filtered and washed with water and diethyl ether to obtain compound **14** (1.4 g, 71 %). ^{1}H NMR (400 MHz, DMSO-d_6) δ 10.67 (d, *J* = 16.2 Hz, 1H), 7.90 (dt, *J* = 8.0, 2.1 Hz, 1H), 7.47 – 7.40 (m, 1H), 7.26

(d, J = 8.1 Hz, 1H), 7.11 (t, J = 7.5 Hz, 1H), 6.16 (d, J = 10.4 Hz, 2H), 5.25 (d, J = 9.7 Hz, 1H). ^{13}C NMR (151 MHz, DMSO-d_6) δ 174.93, 154.34, 138.68, 130.26, 124.49, 123.35, 121.37, 116.40, 89.49.

Preparation of tert-butyl (2-((2-aminoquinolin-4-yl)oxy)ethyl)carbamate (15)

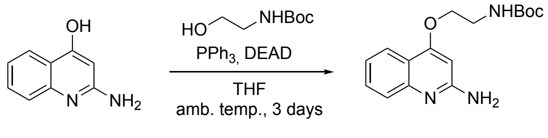

14 15

Quinolinone **14** (425 mg, 2.7 mmol), N-Boc-ethanolamine (642 mg, 4 mmol) and triphenylphosphine (1.5 g, 5.6 mmol) were dissolved in 10 mL freshly distilled THF and cooled to 0 °C. Diethyl azodicarboxylate (DEAD, 1.7 g, 4 mmol) was added dropwise under Ar atmosphere. The mixture was allowed to reach ambient temperature and was stirred for 3 days. The solvent was removed in vacuo and the product purified by SiO_2 column chromatography (eluent: 10% MeOH in EtOAc) to obtain the title compound **15** as a white powder (360 mg, 45%). ^{1}H NMR (400 MHz, DMSO-d_6) δ 7.91 (dd, J = 8.1, 1.5 Hz, 1H), 7.43 (dd, J = 8.4, 6.7, 1.5 Hz, 1H), 7.37 (dd, J = 8.5, 1.3 Hz, 1H), 7.12 (t, 1H), 7.07 (dd, 1H), 6.24 (d, J = 4.4 Hz, 2H), 6.15 (s, 1H), 4.04 (t, J = 5.3 Hz, 2H), 3.44 (q, J = 5.5 Hz, 2H), 1.39 (s, 9H). ^{13}C NMR (100 MHz, DMSO-d_6) δ 160.91, 159.46, 155.81, 148.84, 129.41, 124.80, 121.71, 120.18, 116.75, 90.48, 77.81, 66.93, 28.23.

Preparation of di-tert-butyl (((((4-(4-azidobutoxy)pyridine-2,6-dicarbonyl)bis(azanediyl))bis(quinoline-2,4-diyl))bis(oxy))bis(ethane-2,1-diyl))dicarbamate (16)

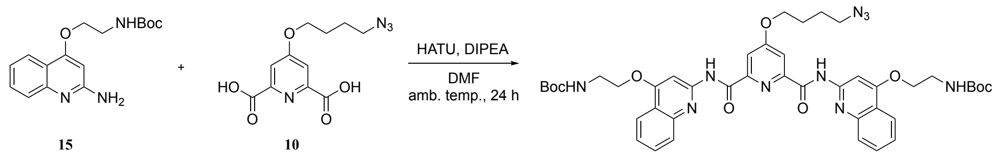

15 10 16

To a solution of compound **10** (32 mg, 0.1 mmol) and **15** (70 mg, 0.2 mmol) in 6 mL dry DMF, DIPEA (100 µL, 0.5 mmol) followed by HATU (130 mg, 0.3 mmol) were added. The reaction mixture was stirred under N_2 atmosphere for 24 h. The progress of the reaction mixture was monitored by LCMS. After the completion of the reaction (slight amount **15** was unreacted) the solvent was evaporated, and water was added. After sonication for few minutes, the solid was filtered and purified by SiO_2 column chromatography (eluent: 3% MeOH in DCM), and compound **16** was obtained (52 mg, 54 %). ^{1}H NMR (400 MHz, DMSO-d_6) δ 12.04 (s, 2H), 8.24 (d, J = 8.4 Hz, 2H), 8.05 (d, J = 2.3 Hz, 2H), 7.95 (s, 1H), 7.93 (s, 3H), 7.77 (ddd, J = 8.6, 7.1, 1.7 Hz, 2H), 7.51 (t, J = 7.5 Hz, 2H), 7.21 (t, J = 5.9 Hz, 2H), 4.35 (t, J = 6.3 Hz, 2H), 4.27 (t, J = 5.3 Hz, 4H), 3.54 (d, J = 5.4 Hz, 2H), 3.47 (d, J = 6.8 Hz, 4H), 1.92 – 1.85 (m, 2H), 1.78 – 1.71 (m, 2H), 1.40 (s, 18H). ^{13}C NMR (151 MHz, DMSO-d_6) δ 167.67, 163.73, 162.79, 156.32, 152.83, 151.48, 147.27, 131.18, 127.12, 125.00, 122.71, 119.67, 112.58, 95.39, 78.34, 68.77, 68.31, 50.76, 28.71, 25.96, 25.37.

Preparation of 2,2'-((((4-(4-azidobutoxy)pyridine-2,6-dicarbonyl)bis(azanediyl))bis(quinoline-2,4-diyl))bis(oxy))bis(ethan-1-aminium) (GL7)

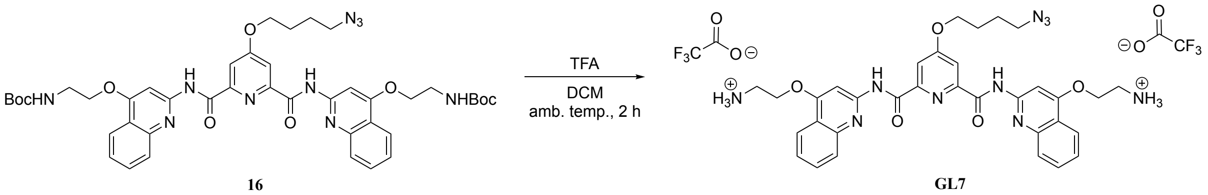

16 GL7

To solution of compound **16** (23 mg, 0.027 mmol) in 3 mL DCM, 0.5 mL TFA was added, and the reaction mixture was stirred at room temperature for 2 h. LCMS was checked to confirm the completion of reaction. The solvent was evaporated, and diethyl ether was added to the reaction mixture and sonicated. The solid was filtered and washed with diethyl ether to obtain **GL7** as a light pink powder (21 mg, 89 %). ^{1}H NMR (400 MHz, DMSO-*d*$_6$) δ 12.09 (s, 1H), 8.44 (d, *J* = 8.3 Hz, 1H), 8.11 (s, 2H), 7.99 – 7.88 (m, 2H), 7.80 (t, 1H), 7.56 (t, *J* = 7.6 Hz, 1H), 4.51 (t, *J* = 5.0 Hz, 2H), 4.35 (t, *J* = 6.3 Hz, 1H), 3.47 (t, *J* = 6.6 Hz, 3H), 1.95 – 1.83 (m, 1H), 1.76 (q, *J* = 7.0 Hz, 1H). ^{13}C NMR (151 MHz, DMSO-*d*$_6$) δ 167.25, 163.37, 161.71, 157.89, 152.34, 151.03, 147.04, 130.86, 126.77, 124.57, 122.72, 119.04, 112.15, 95.05, 68.34, 65.17, 50.32, 38.26, 25.53, 24.93. HRMS: [M+H]$^+$ calc. 651.2786 ($C_{33}H_{34}N_{10}O_5$); found 651.2732 (*m/z*).

GL8

Synthesis of precursor **17** is described in Spagnul et al. 2013. *J. Inorg. Biochem*., 2023.[4]

Preparation of 2-azidoethyl 4-(5,15,20-tri(pyridin-4-yl)porphyrin-10-yl)benzoate (18)

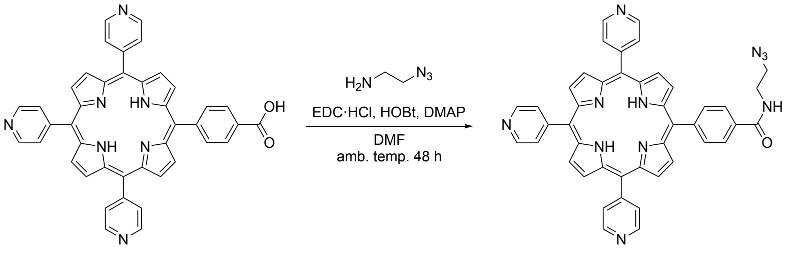

17 18

To a RBF was added compound **17** (100 mg, 0.151 mmol), EDC·HCl (57.9 mg, 0.302 mmol) and HOBt (30.6 mg, 0.227 mmol), along with DMF (4 mL). The mixture was allowed to stir at ambient temperature for 45 min. To the mixture was added a solution of 1-azido-2-bromoethane (26 mg, 0.302 mmol) and DMAP (20,3 mg, 0.166 mmol) in DMF (3 mL). The RBF was shielded from light and was allowed to stir at room temperature for 48h. The progress of the reaction was monitored via LC-MS. The solvent was removed under reduced pressure and the crude was washed with brine and purified by flash chromatography in gradient eluent system (eluent: 3-7% MeOH in DCM). The compound **18** was afforded as a purple solid (61.8 mg, 56%). ^{1}H NMR (400 MHz, Chloroform-*d*) δ 9.06 (d, 6H), 8.91 – 8.81 (m, 8H), 8.31 (d, *J* = 8.2 Hz, 2H), 8.20 (d, *J* = 8.2 Hz, 2H), 8.16 (dt, *J* = 4.3, 1.4 Hz, 6H), 3.85 (dd, *J* = 6.1, 4.5 Hz, 2H), 3.75 (dd, *J* = 6.2, 4.5 Hz, 2H), -2.89 (s, 2H).

Preparation of 4,4'-(10-(4-((2-azidoethoxy)carbonyl)phenyl)-15-(1-metheyliumyl-1λ4-pyridin-4-yl)porphyrin-5,20-diyl)bis(1-methylpyridin-1-ium) (GL8)

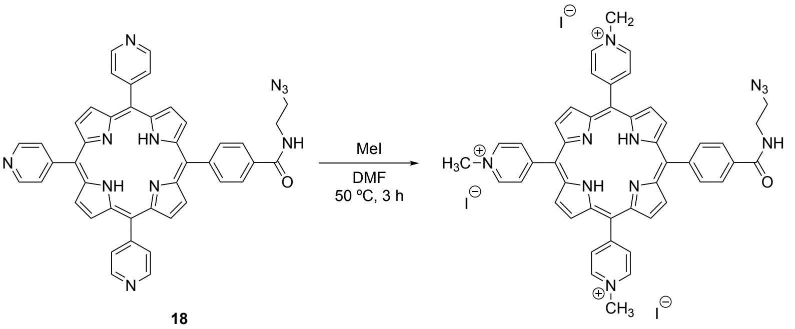

18

GL8

Compound **18** (61.8 mg, 0.085 mmol) was dissolved in DMF (4 mL). To the mixture was added MeI (257 µL, 4.13 mmol). The mixture was shielded from light and heated to 50°C. The progress of the reaction

was monitored via TLC. The reaction mixture was allowed to cool to room temperature and subjected to solvent removal under reduced pressure. The crude was suspended in ice-cold Et_2O and sonicated. The Et_2O was carefully decanted and **GL8** was afforded as a black solid (84 mg, 88%). ^1H NMR (600 MHz, DMSO-d_6) δ 9.56 – 9.48 (m, 6H), 9.34 (t, *J* = 5.4 Hz, 1H), 9.15 (s, 4H), 9.00 (d, *J* = 6.3 Hz, 8H), 8.49 (s, 4H), 8.38 (d, *J* = 7.7 Hz, 2H), 8.34 (d, *J* = 7.7 Hz, 2H), 4.73 (d, *J* = 6.0 Hz, 9H), 3.67 (t, *J* = 5.3 Hz, 2H), 3.64 (t, *J* = 5.3 Hz, 2H), -3.03 (s, 2H). ^{13}C NMR (151 MHz, DMSO-d_6) δ 166.36, 164.90, 156.50, 156.44, 144.24, 143.14, 134.20, 134.15, 132.11, 126.13, 121.87, 115.41, 114.75, 49.97, 47.84. HRMS: $[M+H]^+$ calc. 775.3490 ($C_{47}H_{40}N_{11}O_3^+$); found 774.4245 (*m/z*).

NMR spectra

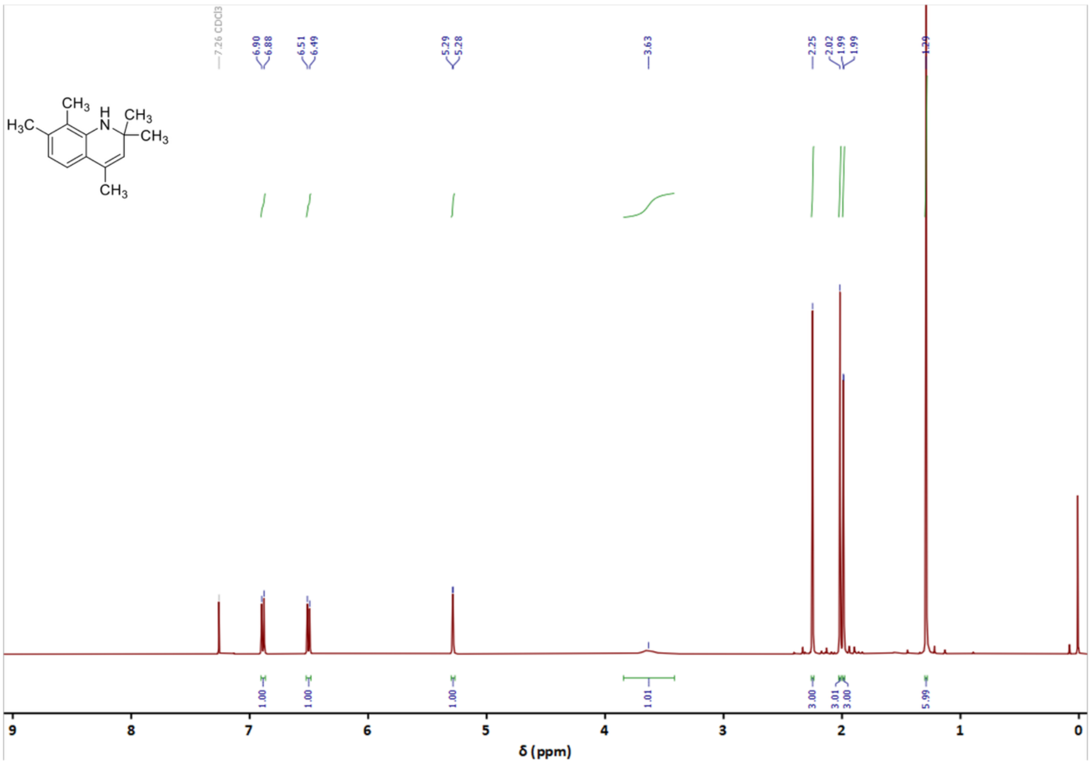

^{1}H NMR spectrum of **1** in chloroform-*d* measured at 400 MHz.

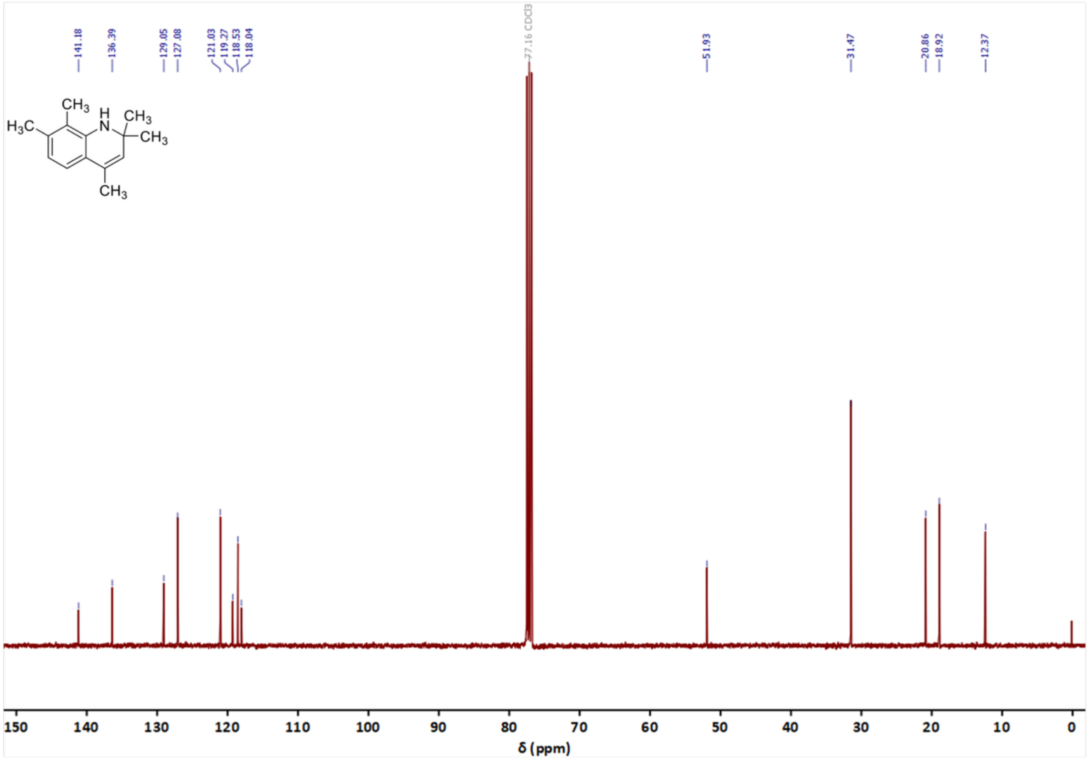

^{13}C NMR spectrum of **1** in chloroform-*d* measured at 100 MHz.

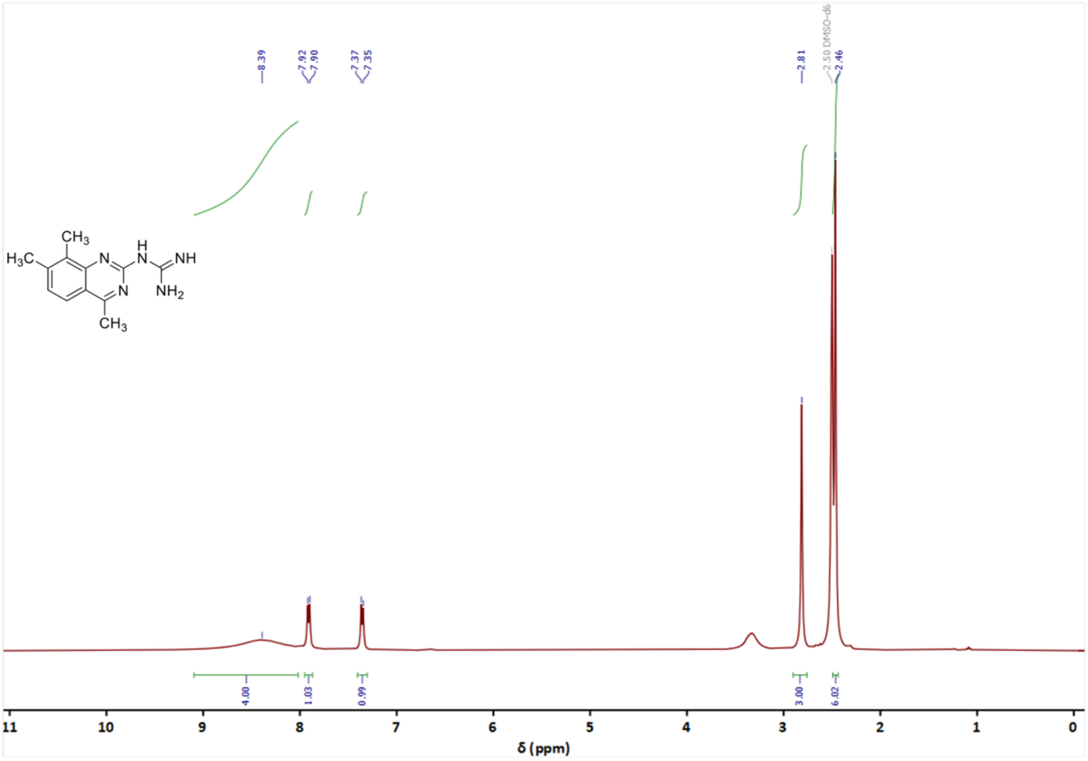

^{1}H NMR spectrum of **2** in DMSO-d_6 measured at 400 MHz.

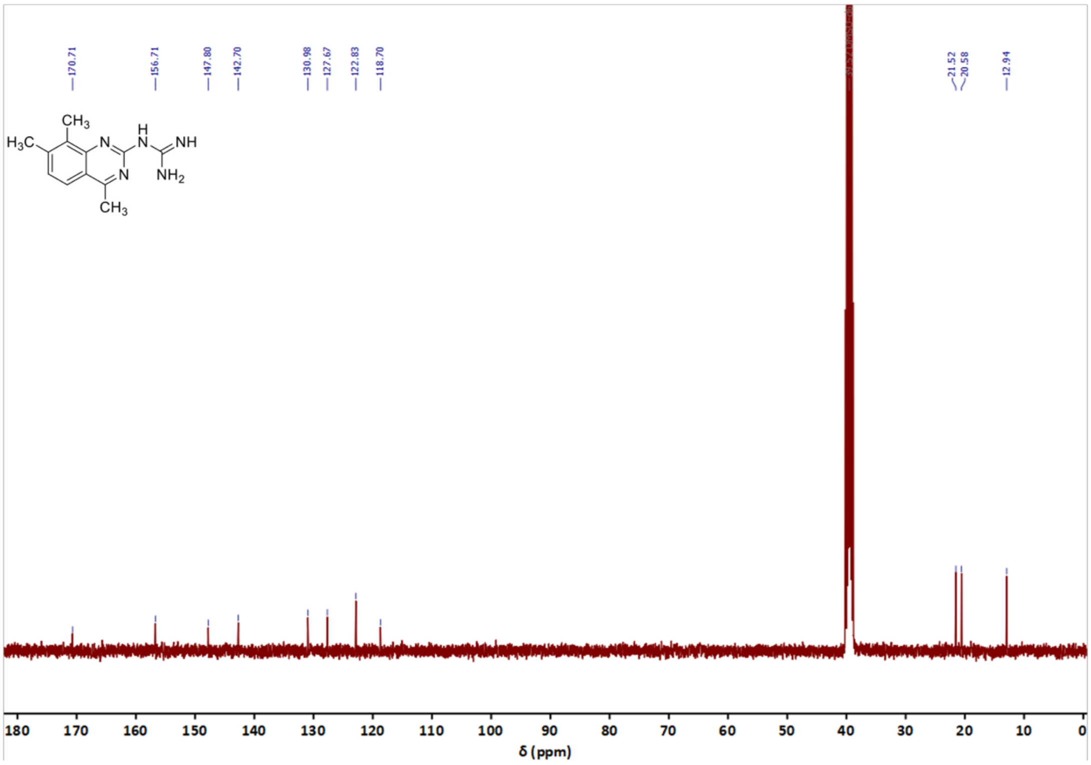

^{13}C NMR spectrum of **2** in DMSO-d_6 measured at 100 MHz.

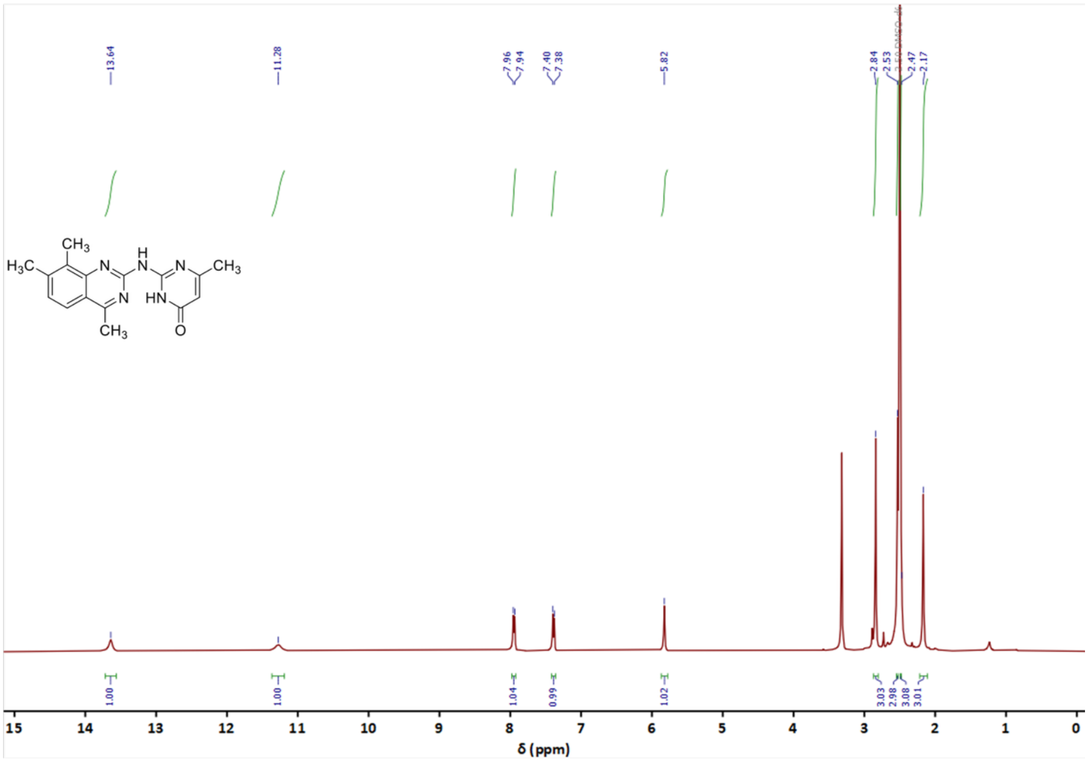

^{1}H NMR spectrum of **3** in chloroform-*d* measured at 400 MHz.

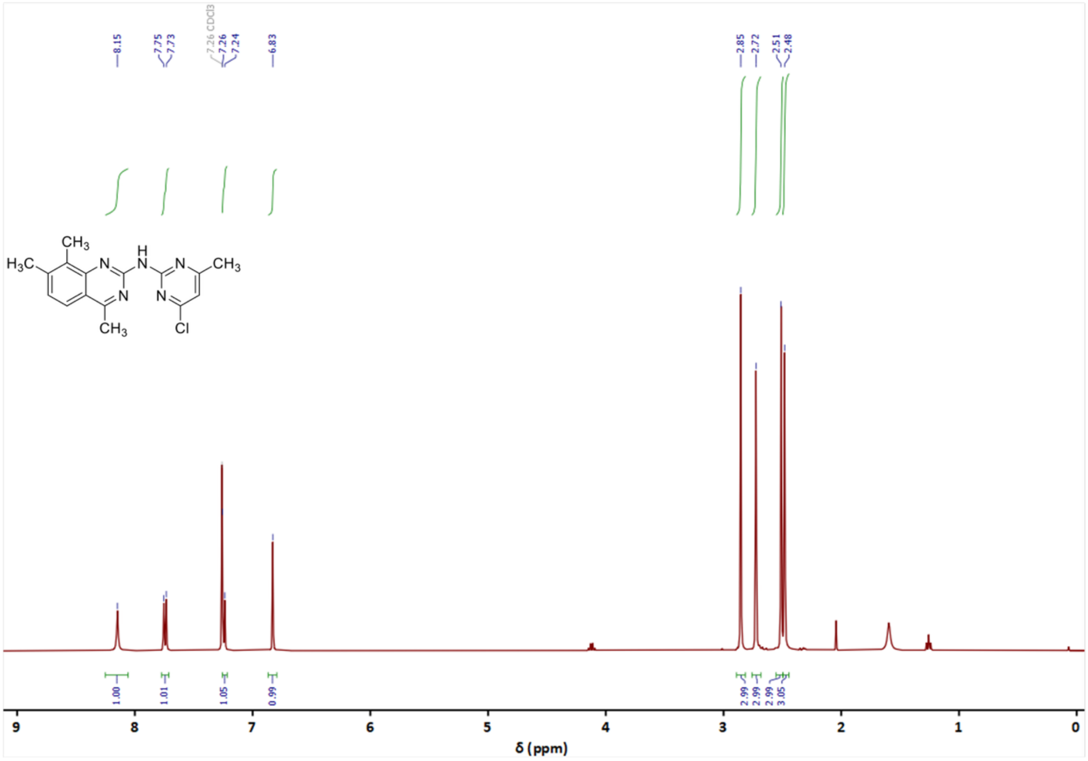

^{1}H NMR spectrum of **4** in chloroform-*d* measured at 400 MHz.

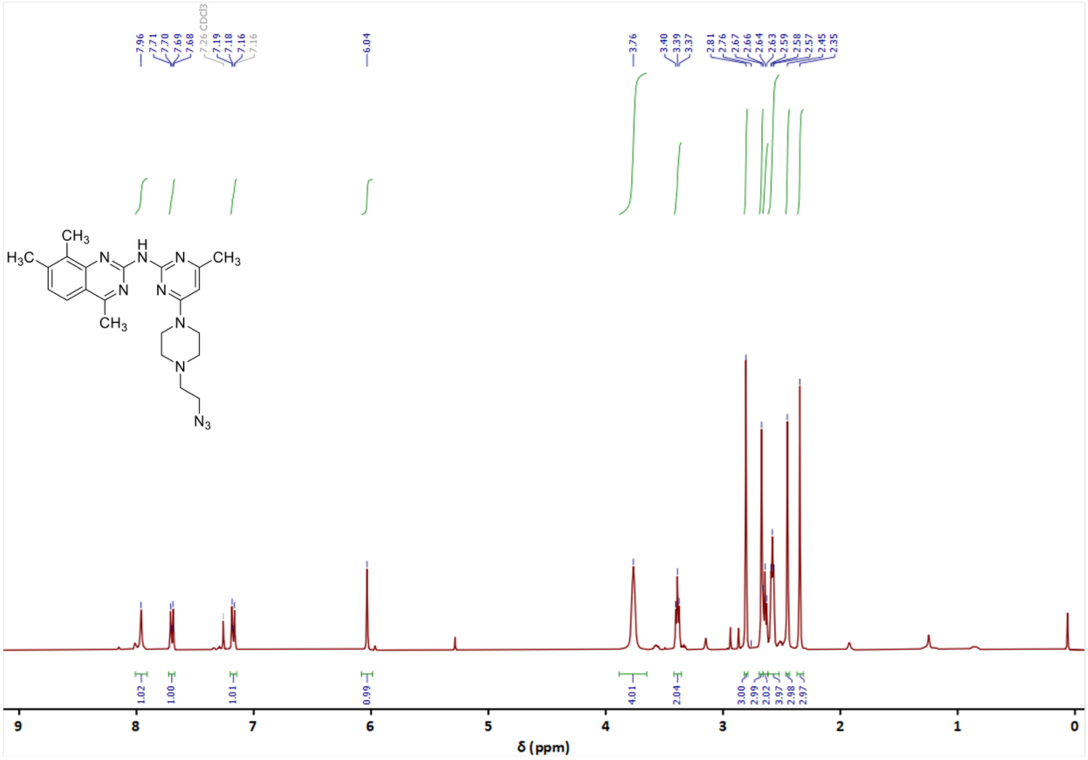

^{1}H NMR spectrum of **GL1** in chloroform-*d* measured at 400 MHz.

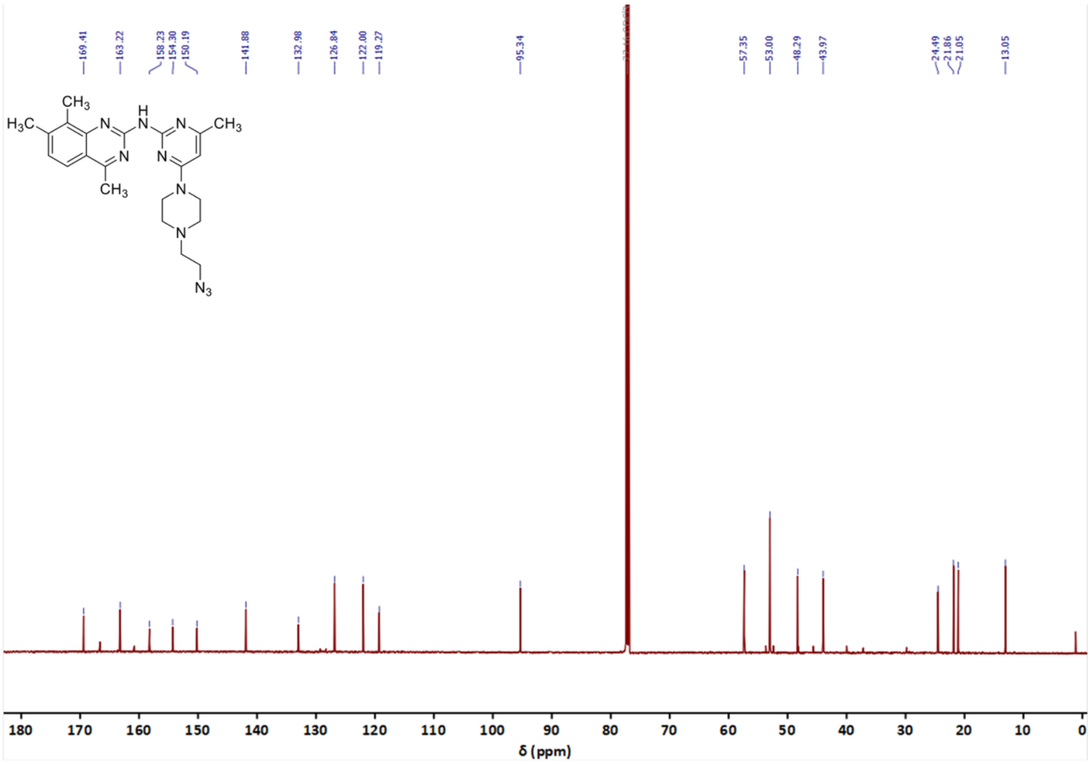

^{13}C NMR spectrum of **GL1** in chloroform-*d* measured at 151 MHz.

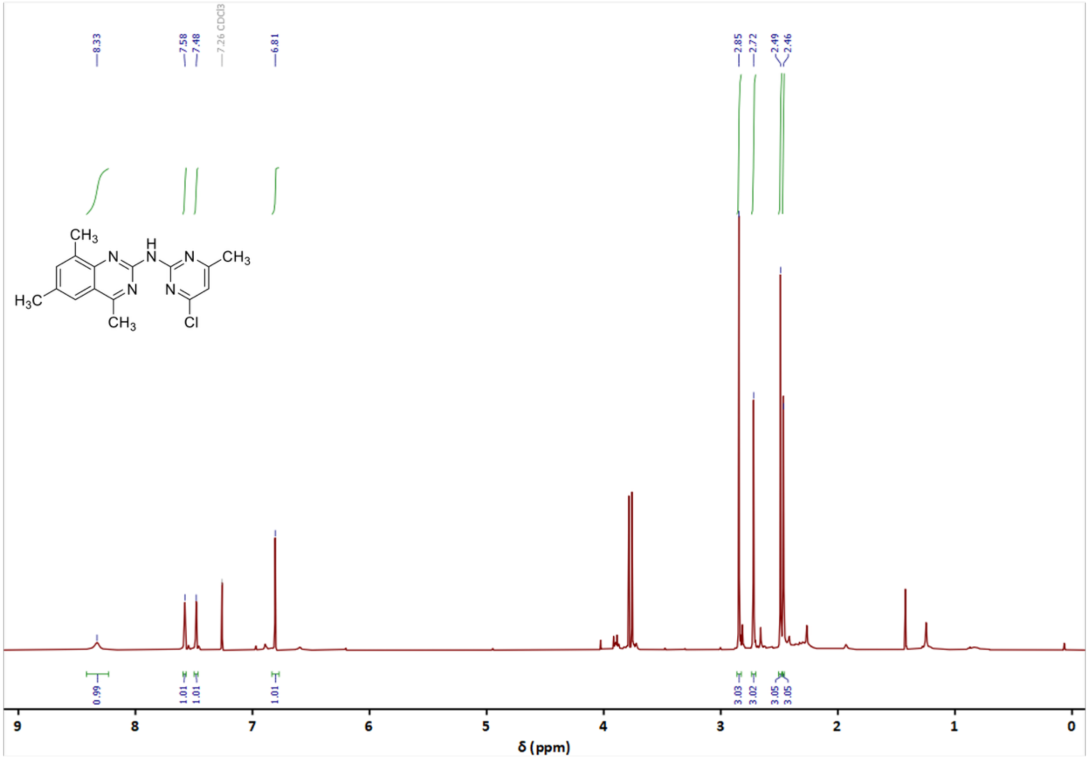

^{1}H NMR spectrum of **6** in chloroform-*d* measured at 400 MHz.

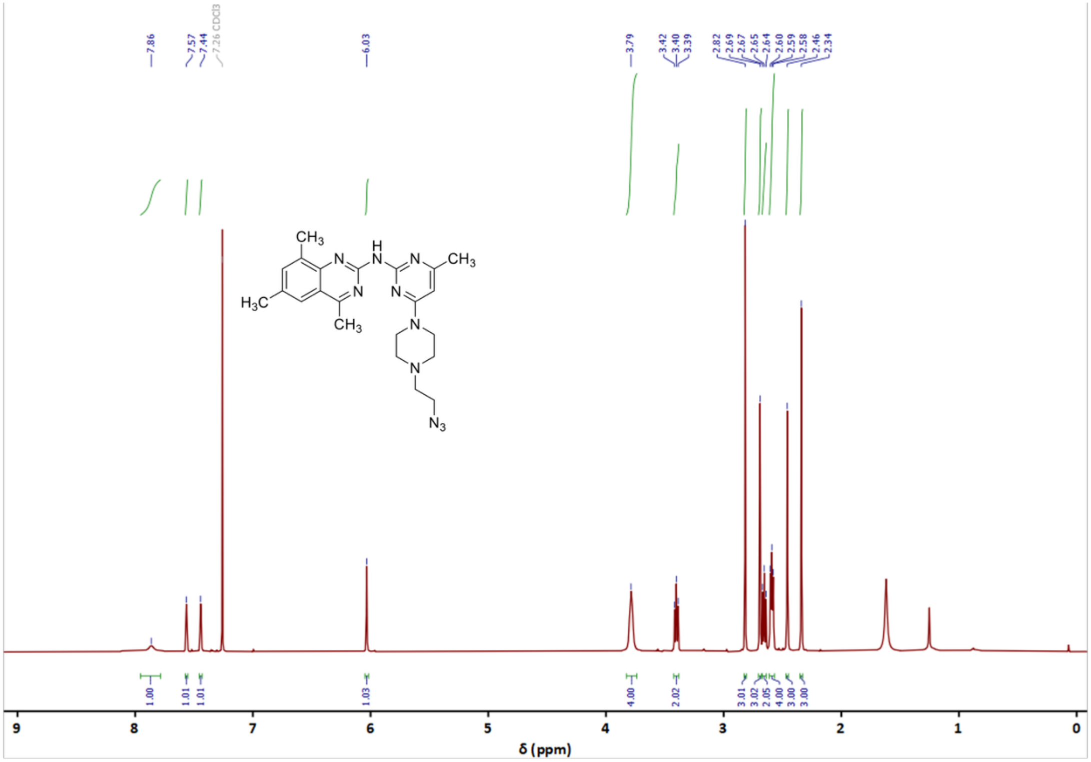

^{1}H NMR spectrum of **GL2** in chloroform-*d* measured at 400 MHz.

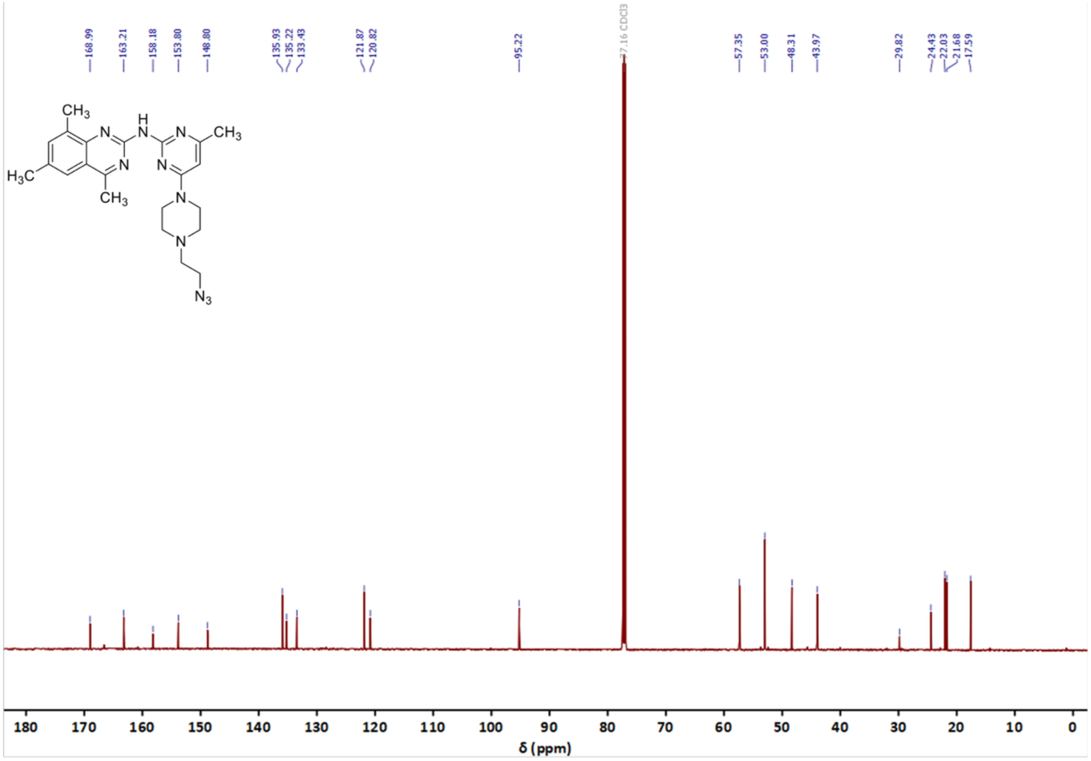

^{13}C NMR spectrum of **GL2** in chloroform-*d* measured at 151 MHz.

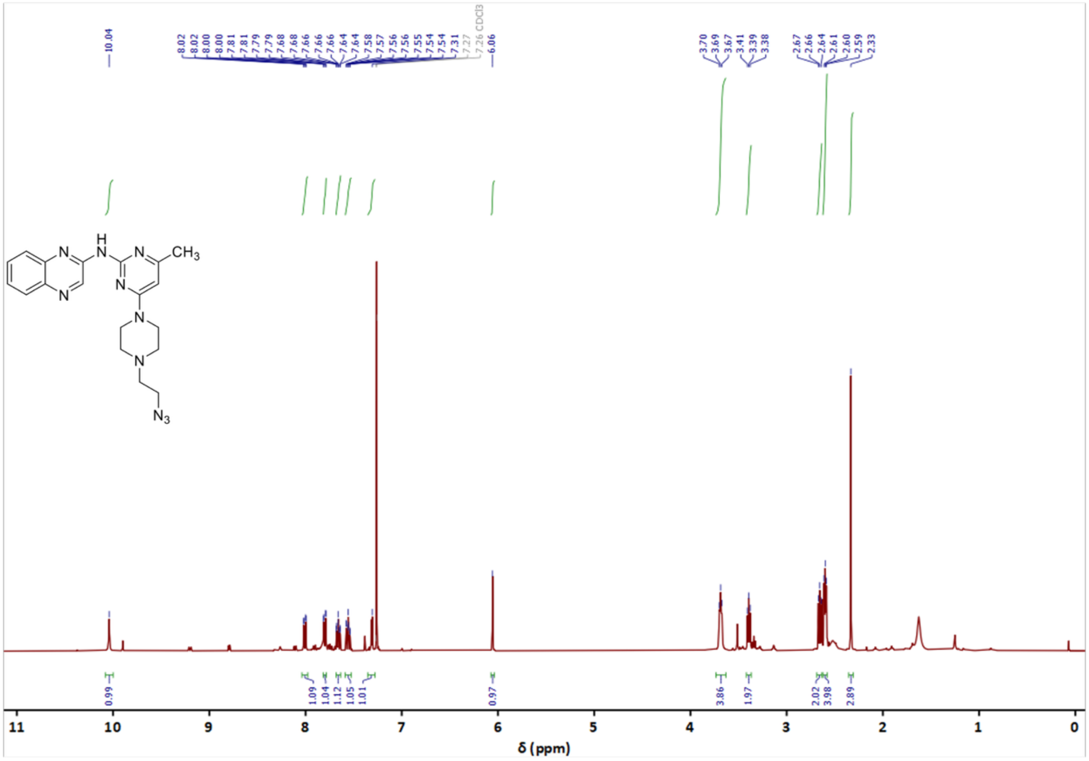

^{1}H NMR spectrum of **GL3** in chloroform-*d* measured at 400 MHz.

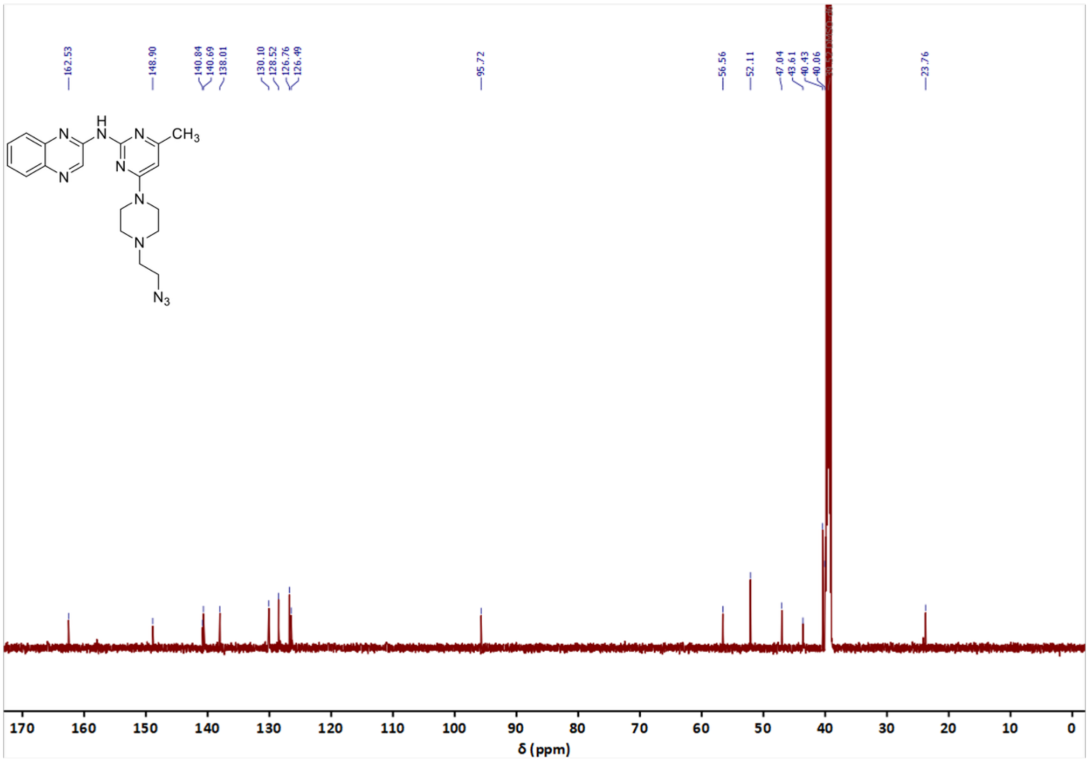

^{13}C NMR spectrum of **GL3** in DMSO-d_6 measured at 151 MHz.

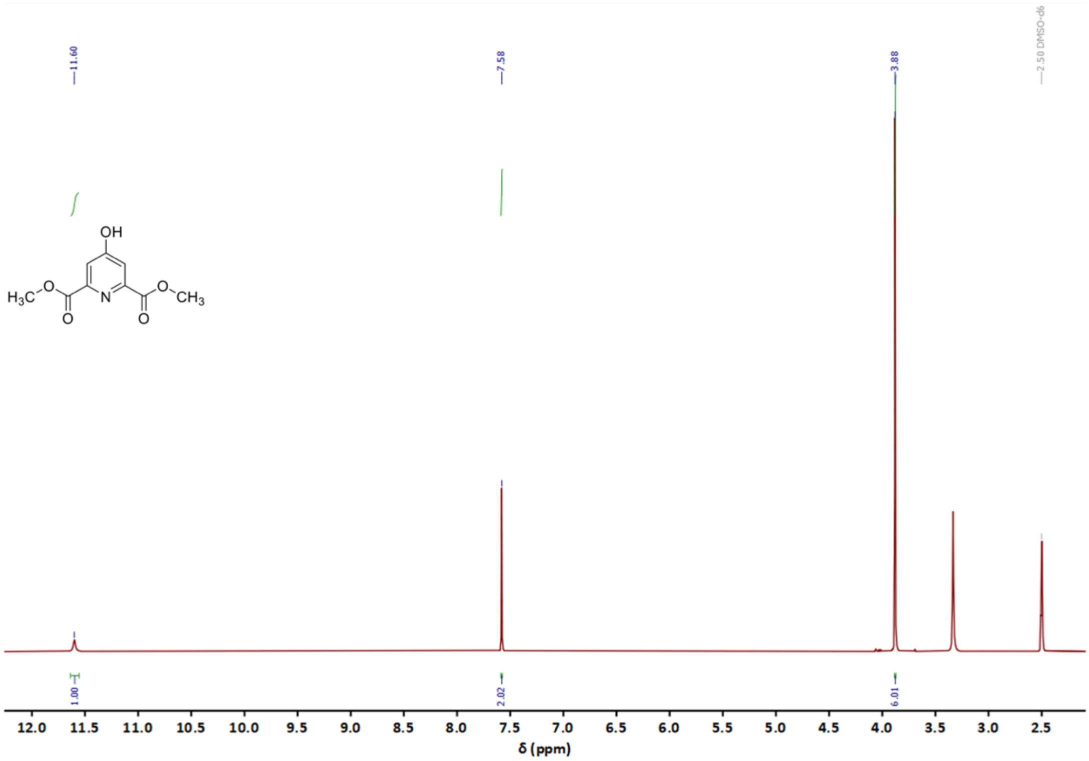

^{1}H NMR spectrum of **8** in DMSO-d_6 measured at 400 MHz.

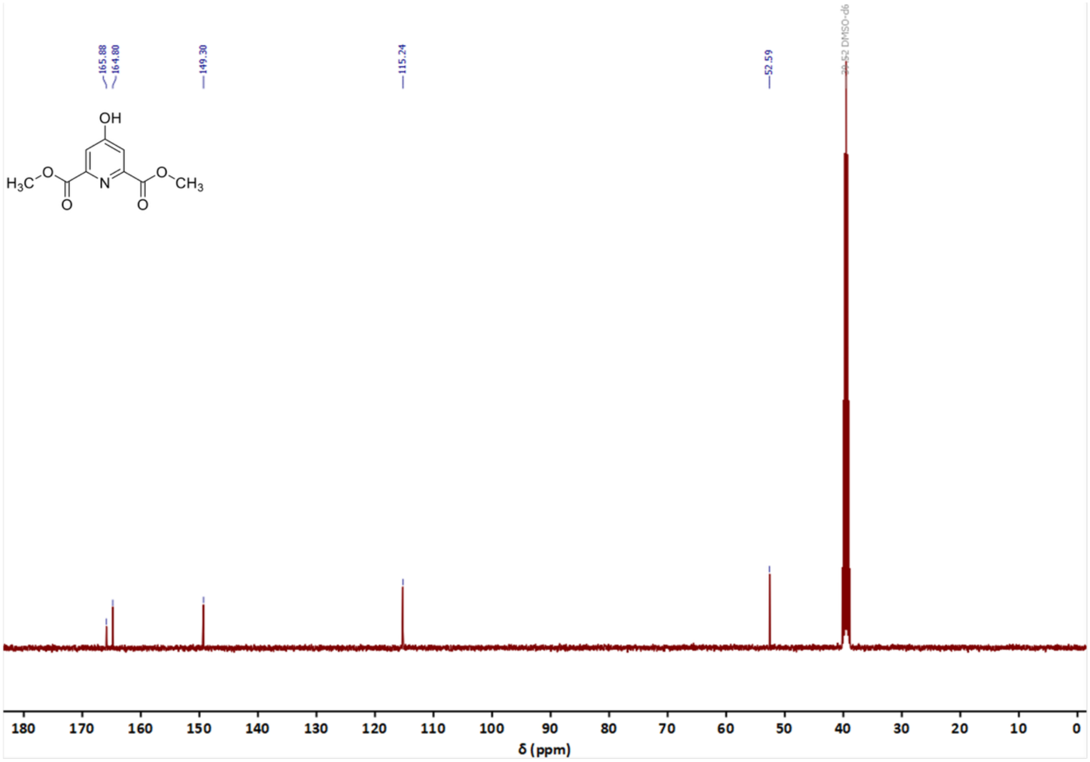

^{13}C NMR spectrum of **8** in DMSO-d_6 measured at 100 MHz.

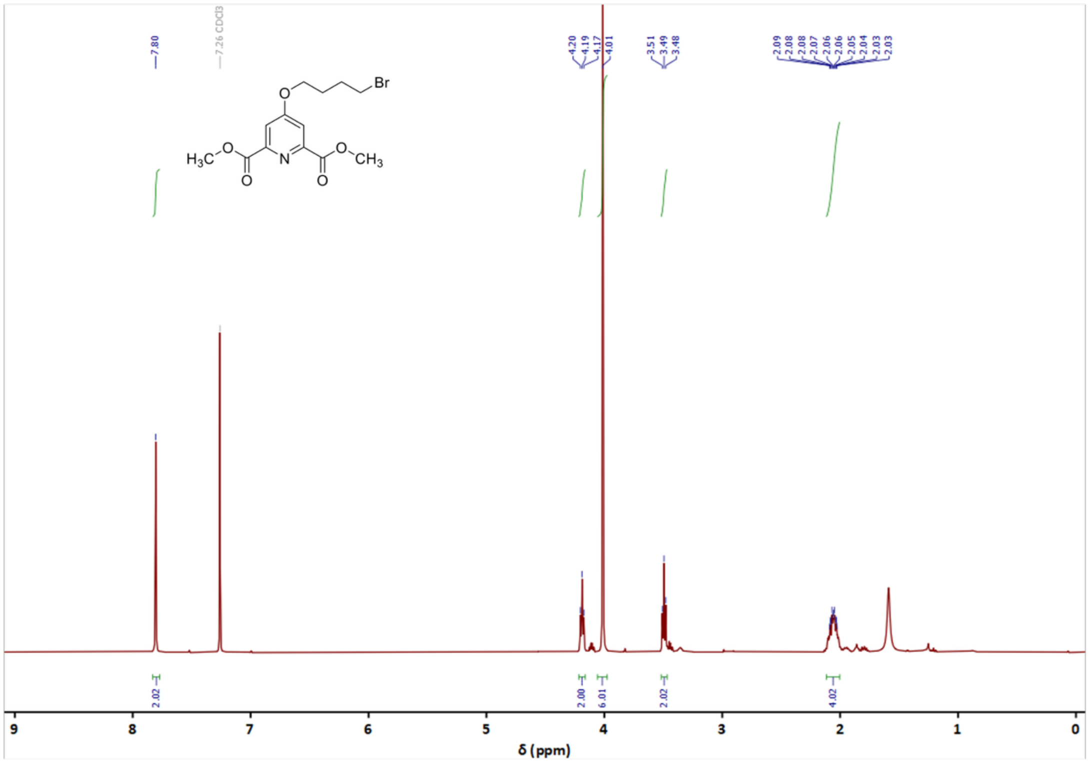

^{1}H NMR spectrum of **9** in chloroform-d measured at 400 MHz.

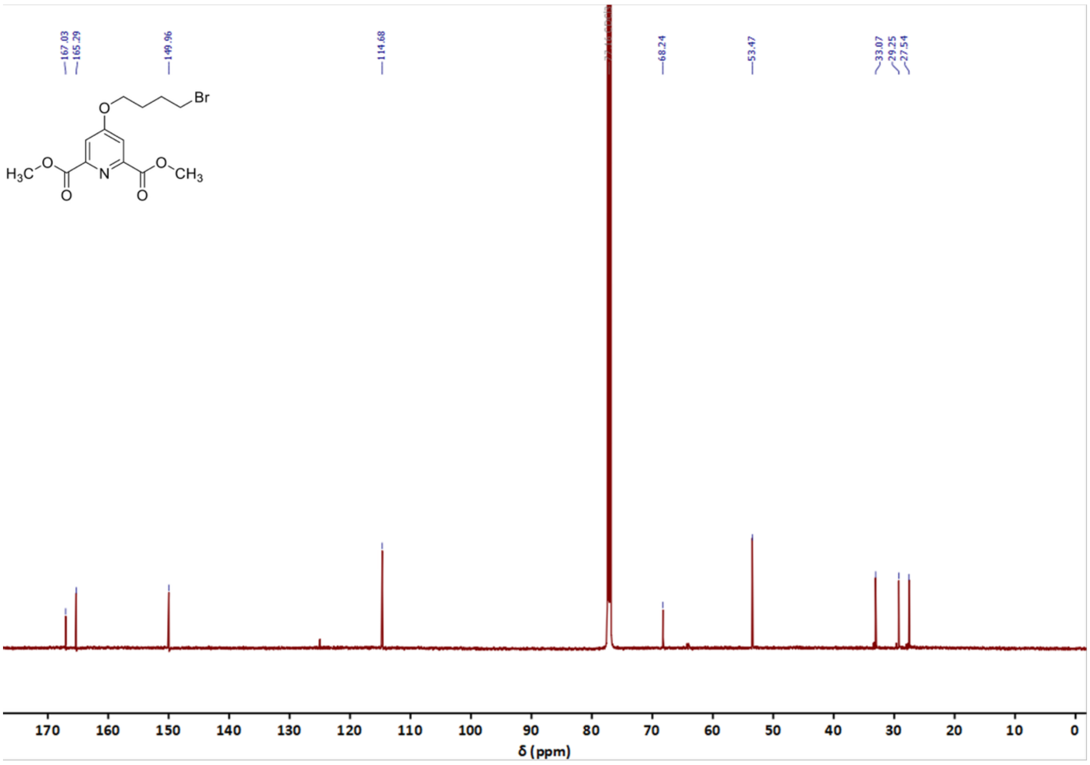

^{13}C NMR spectrum of **9** in chloroform-*d* measured at 151 MHz.

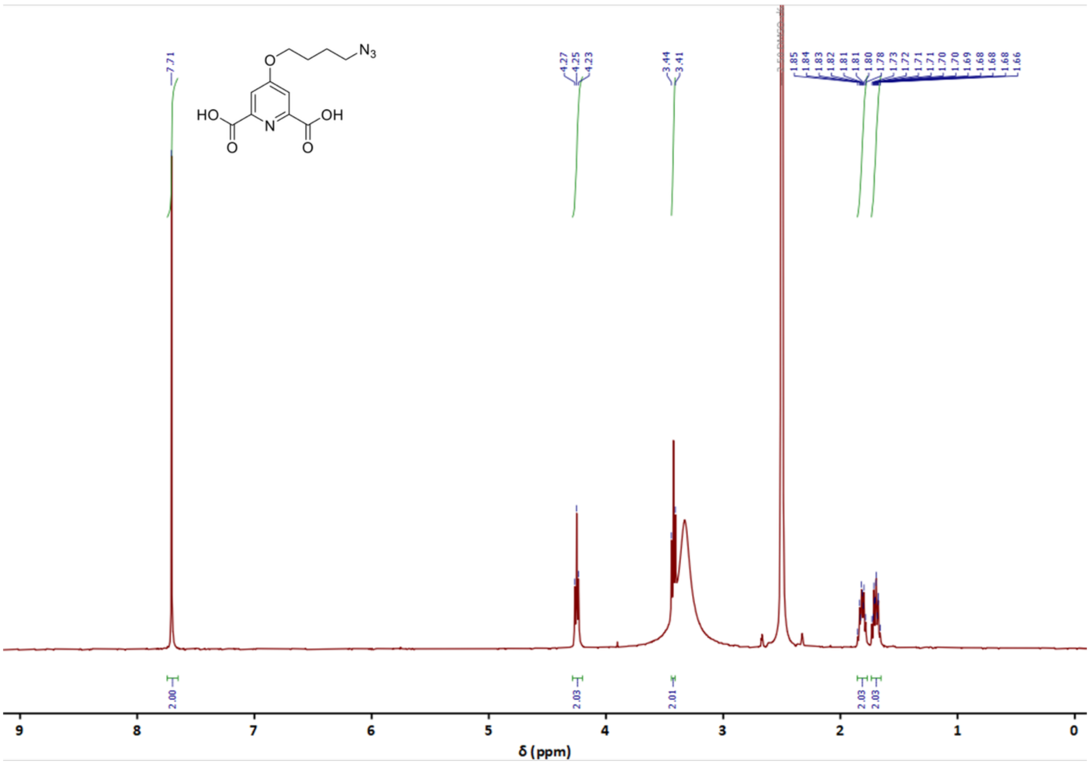

^{1}H NMR spectrum of **10** in DMSO-d_6 measured at 400 MHz.

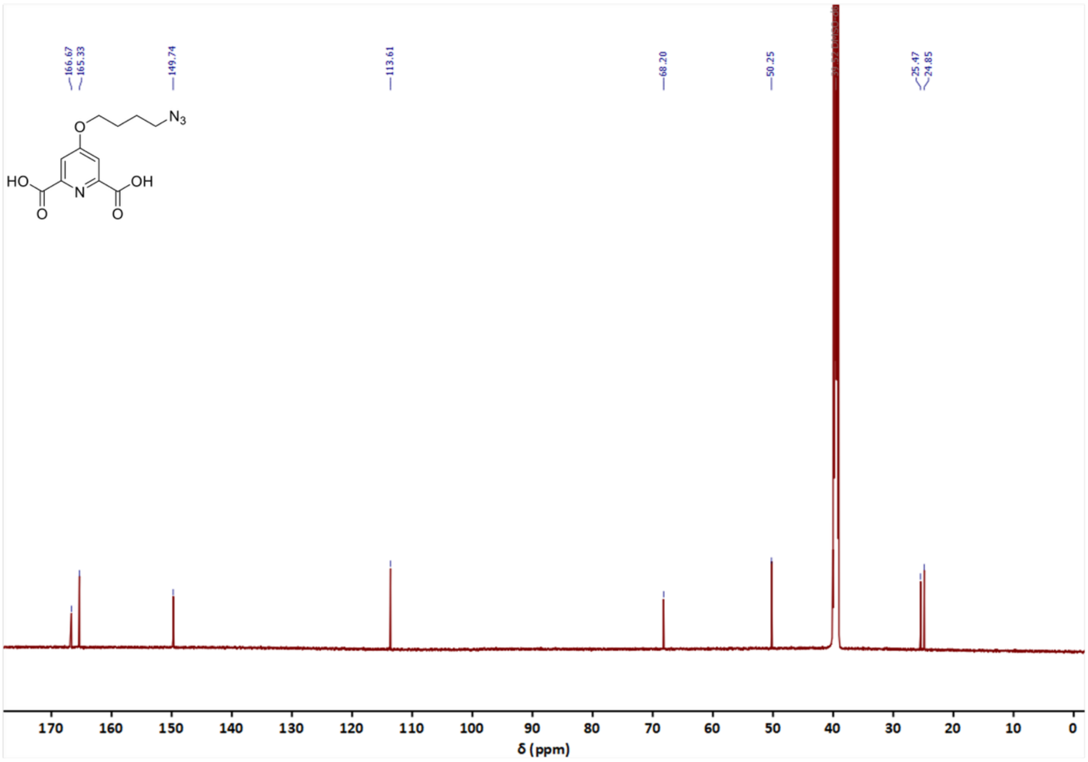

^{13}C NMR spectrum of **10** in DMSO-d_6 measured at 151 MHz.

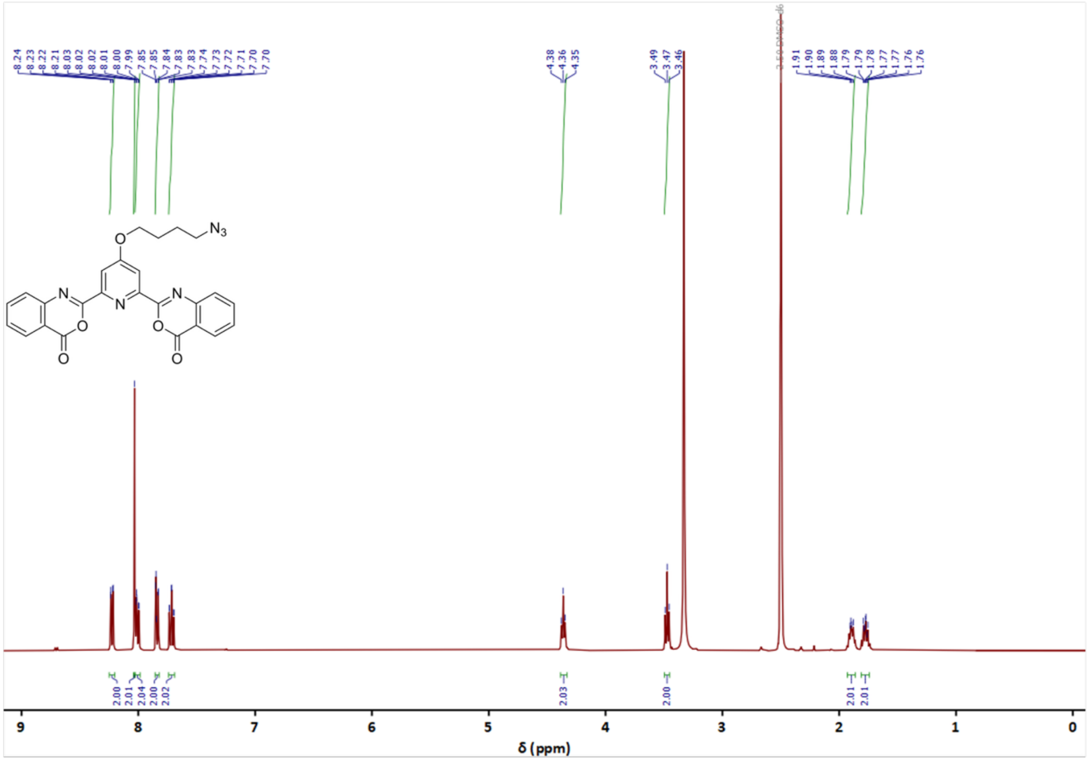

^{1}H NMR spectrum of **11** in DMSO-d_6 measured at 400 MHz.

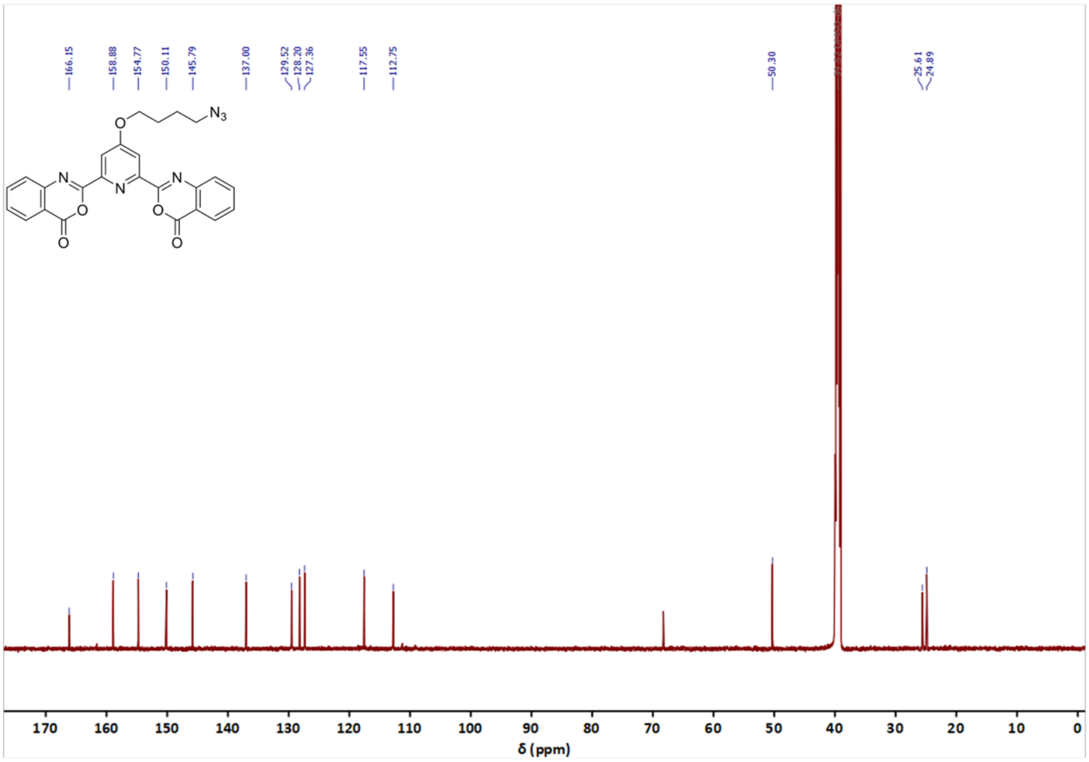

^{13}C NMR spectrum of **11** in DMSO-d_6 measured at 151 MHz.

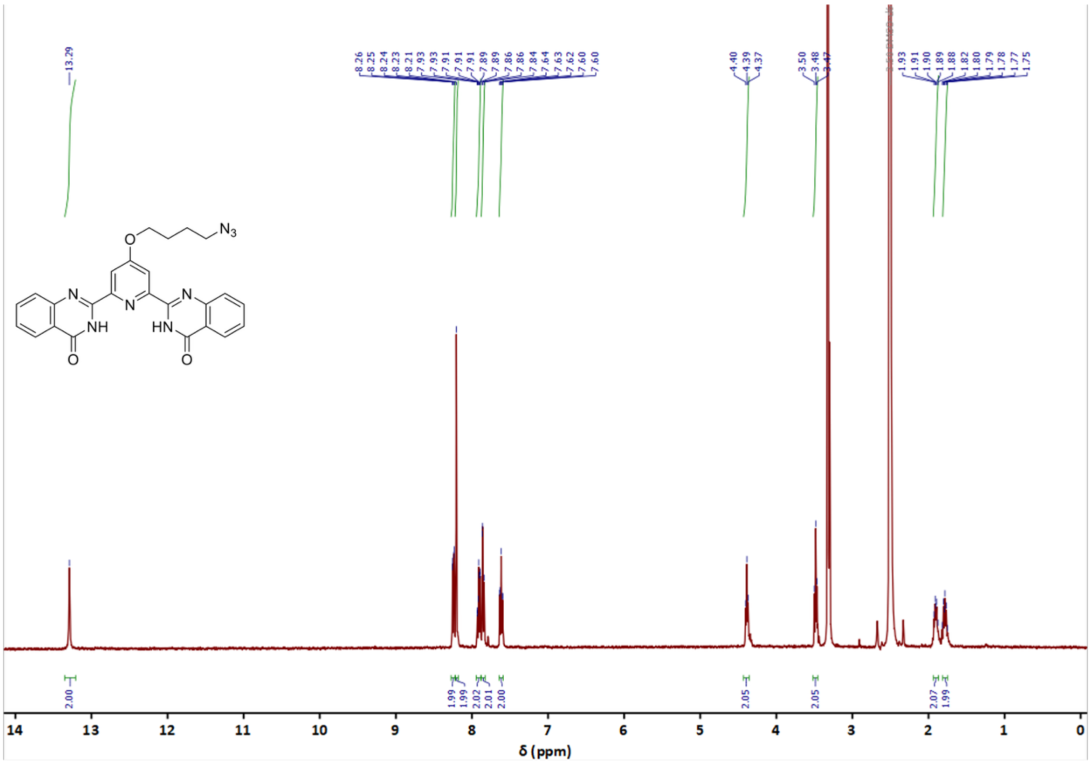

^{1}H NMR spectrum of **12** in DMSO-d_6 measured at 400 MHz.

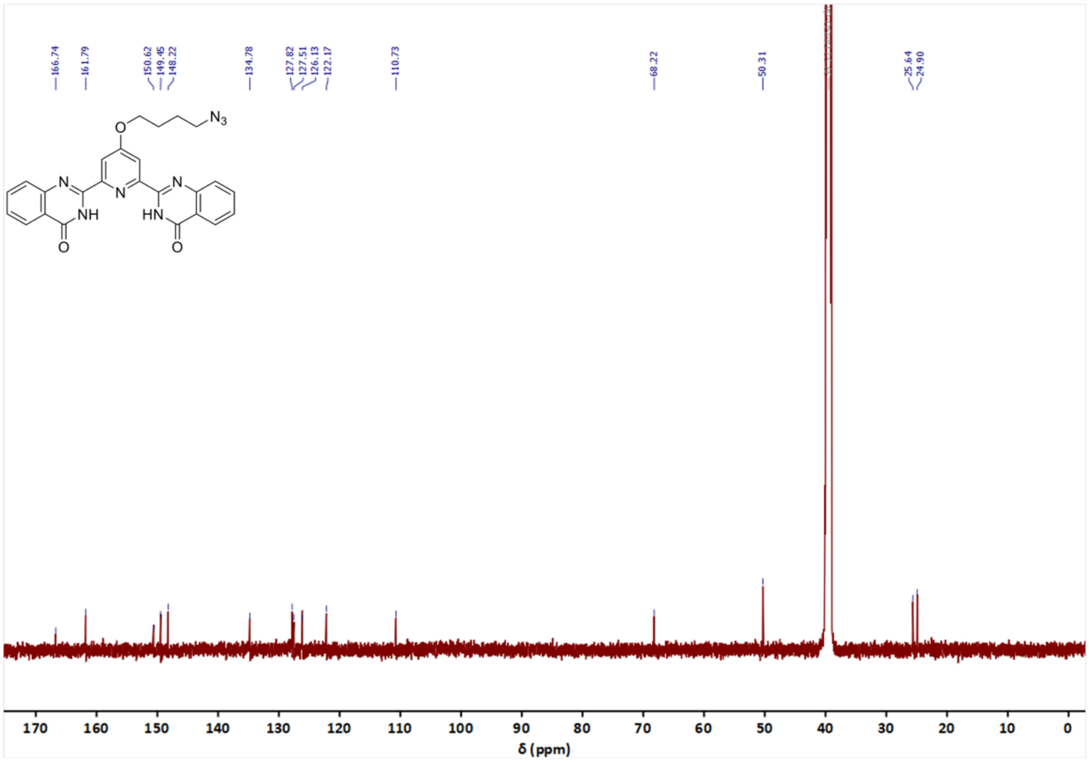

^{13}C NMR spectrum of **12** in DMSO-d_6 measured at 151 MHz.

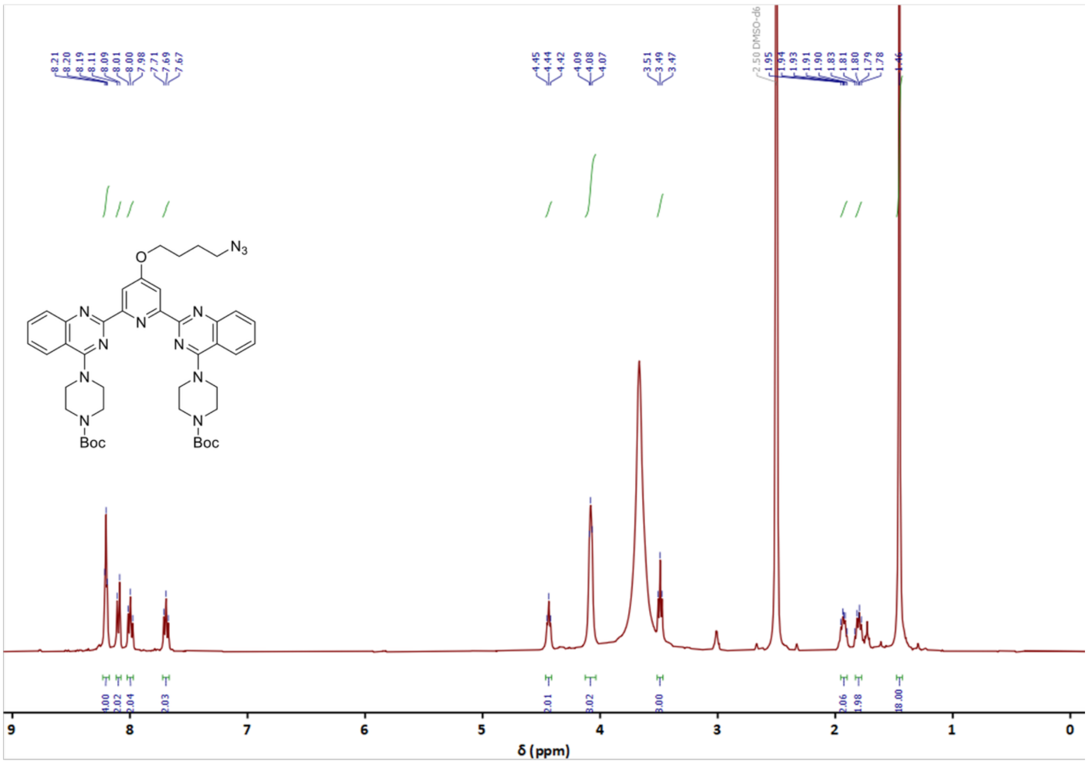

^{1}H NMR spectrum of **13** in DMSO-d_6 measured at 400 MHz.

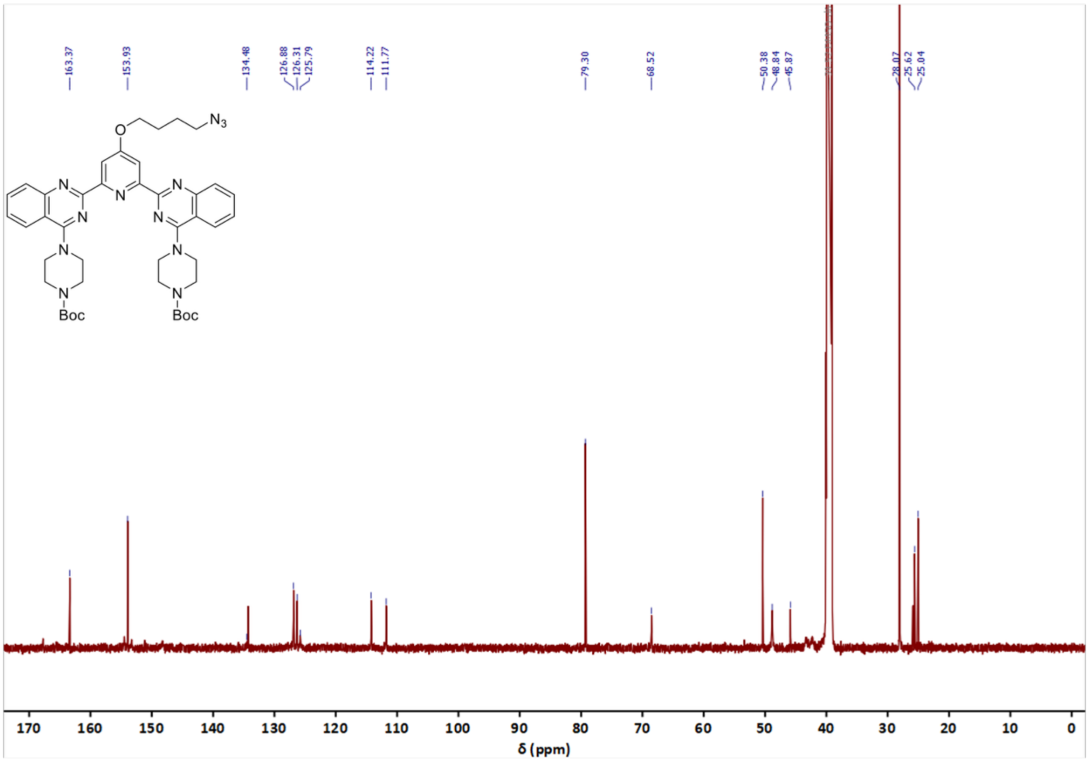

^{13}C NMR spectrum of **13** in DMSO-d_6 measured at 151 MHz.

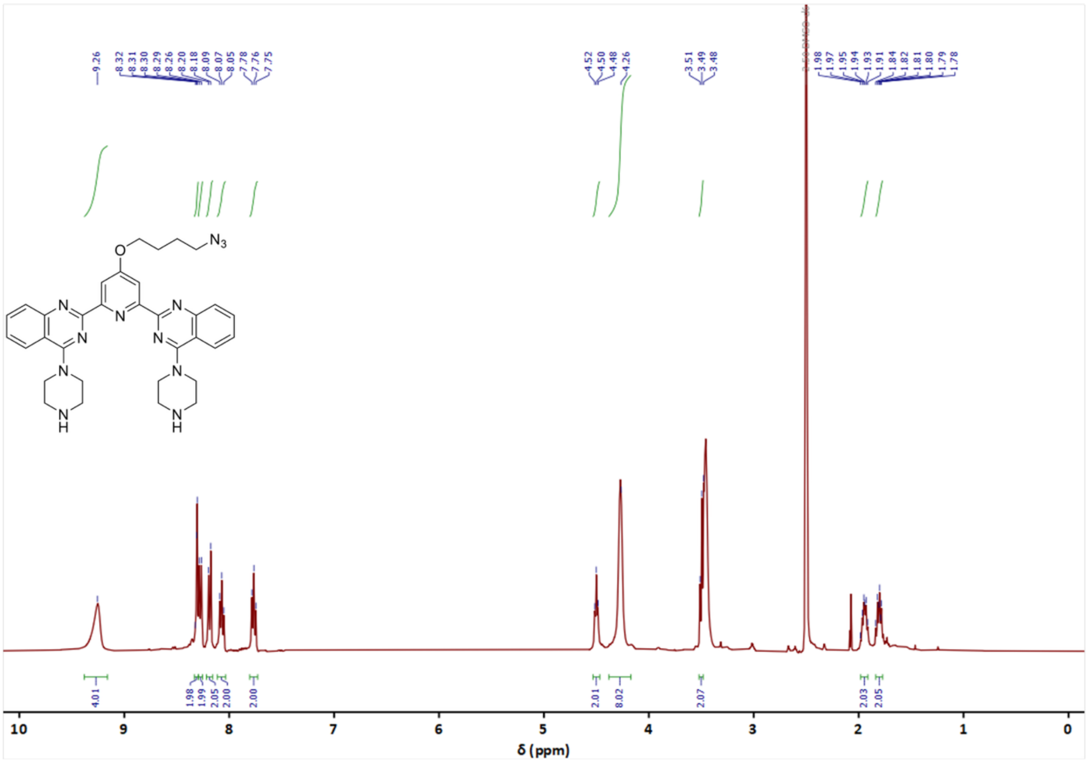

^{1}H NMR spectrum of **GL4** in DMSO-d_6 measured at 400 MHz.

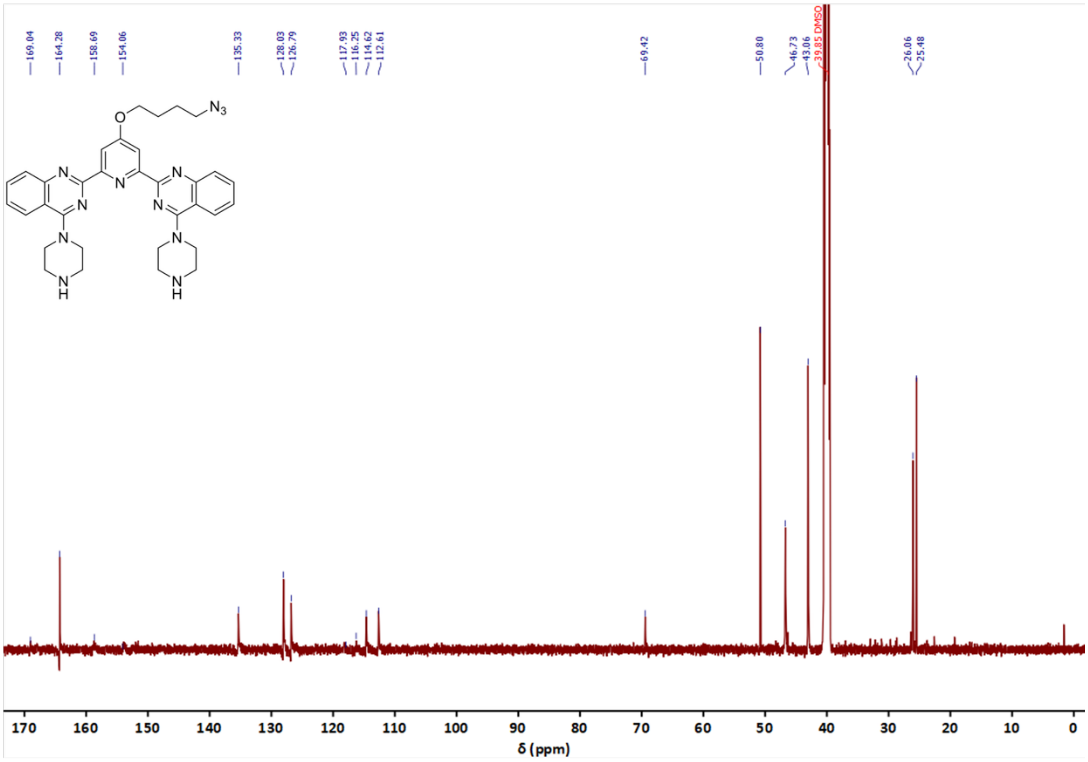

^{13}C NMR spectrum of **GL4** in DMSO-d_6 measured at 151 MHz.

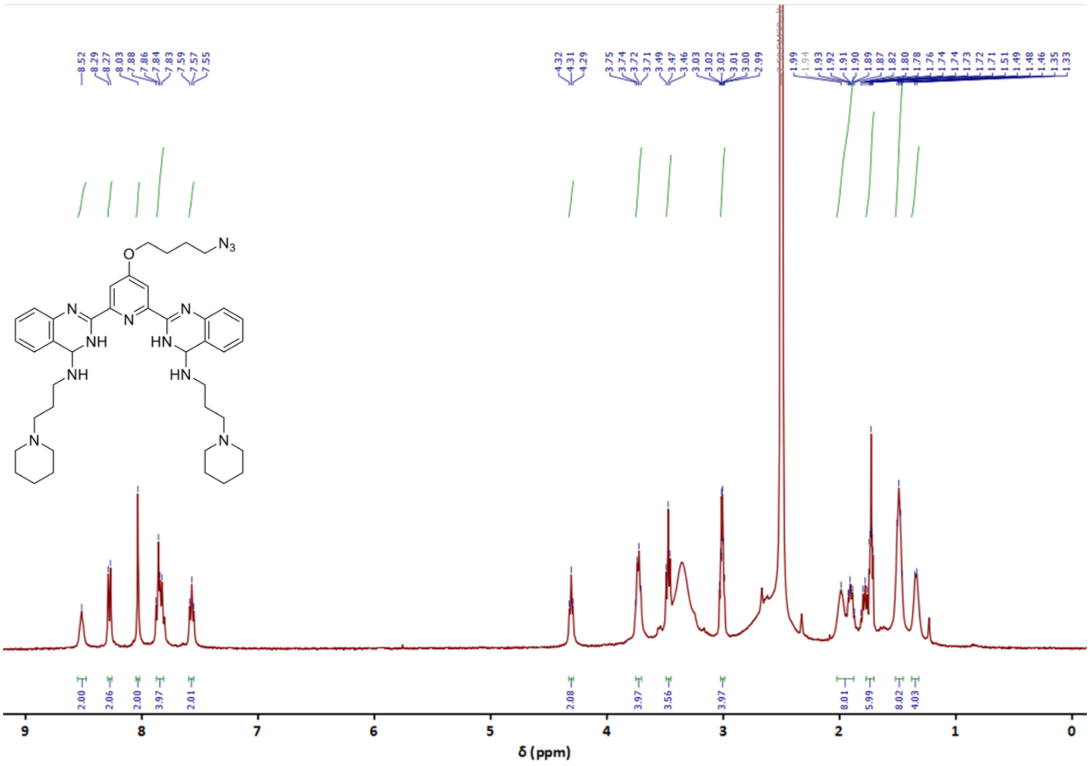

^{1}H NMR spectrum of **GL5** in DMSO-d_6 measured at 400 MHz.

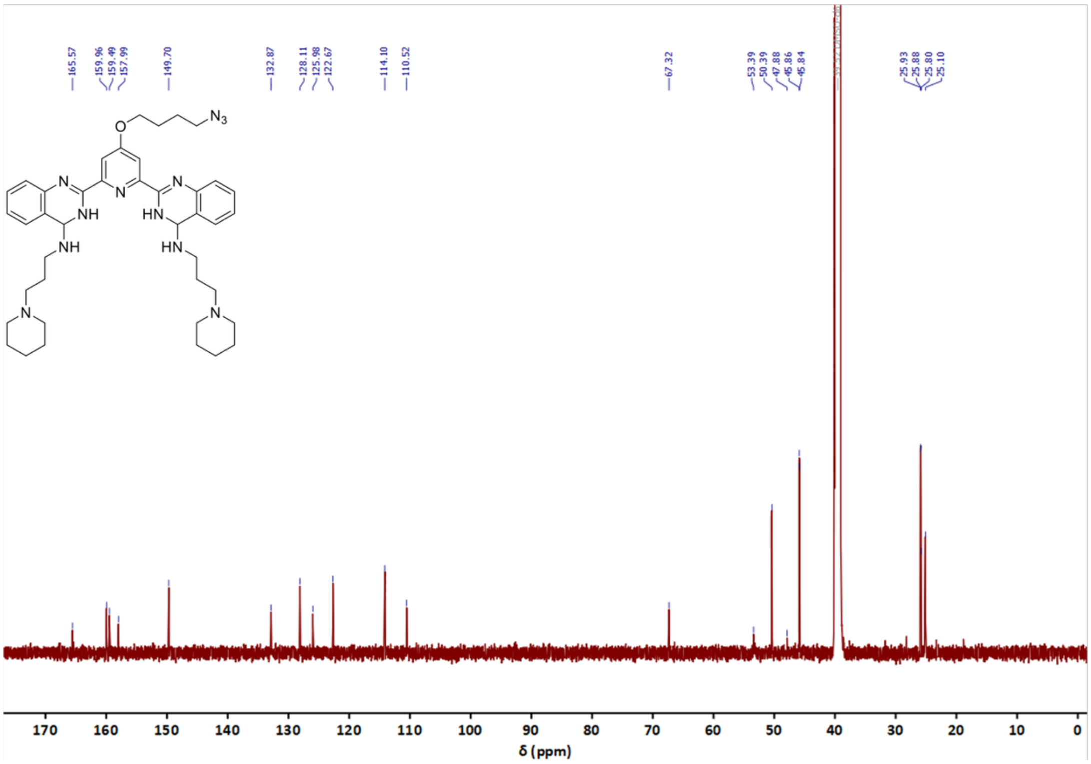

^{13}C NMR spectrum of **GL5** in DMSO-d_6 measured at 151 MHz.

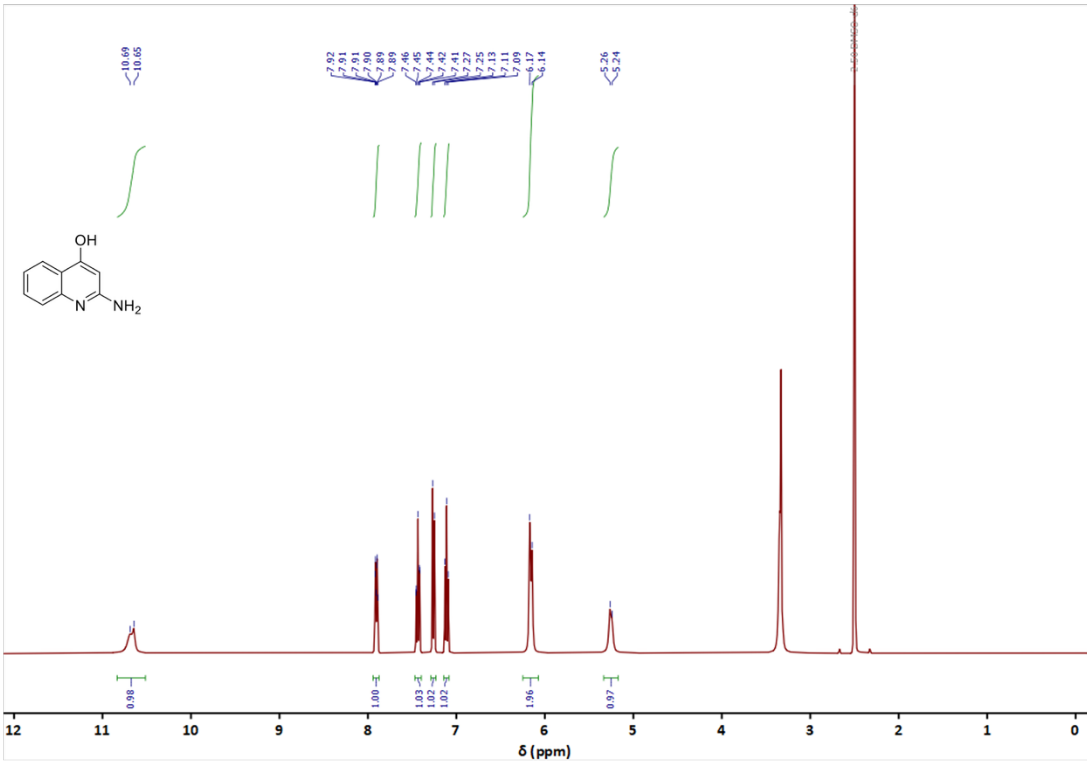

^{1}H NMR spectrum of **14** in DMSO-d_6 measured at 400 MHz.

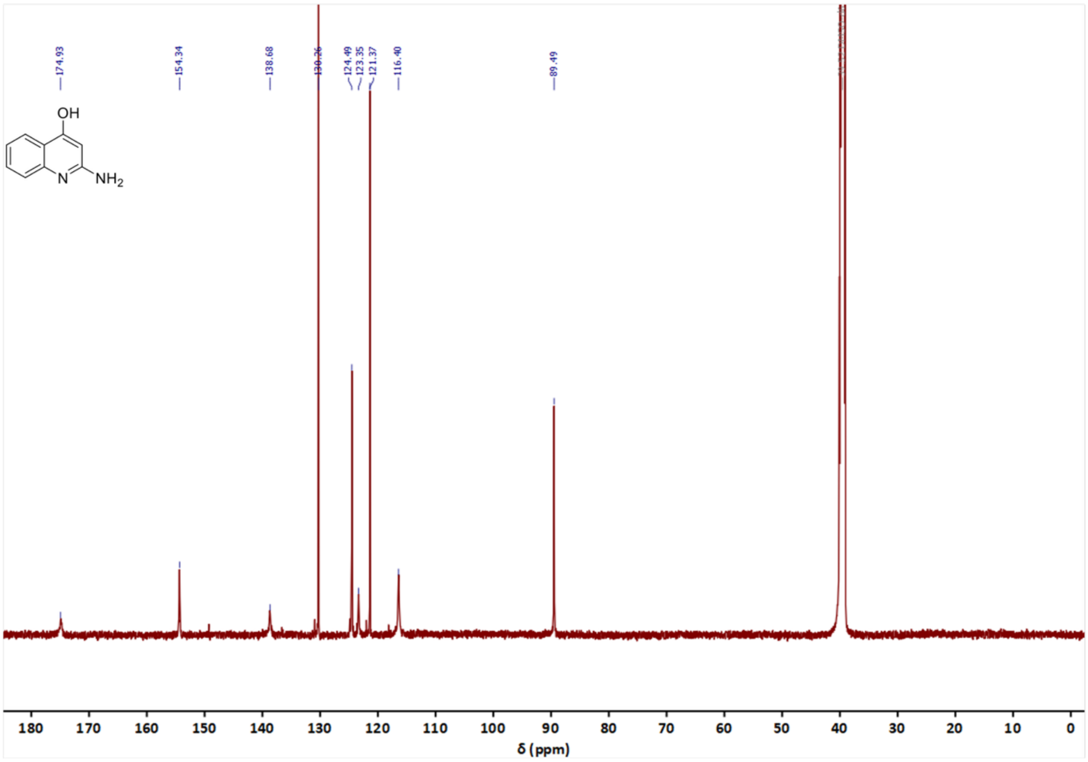

^{13}C NMR spectrum of **14** in DMSO-d_6 measured at 151 MHz.

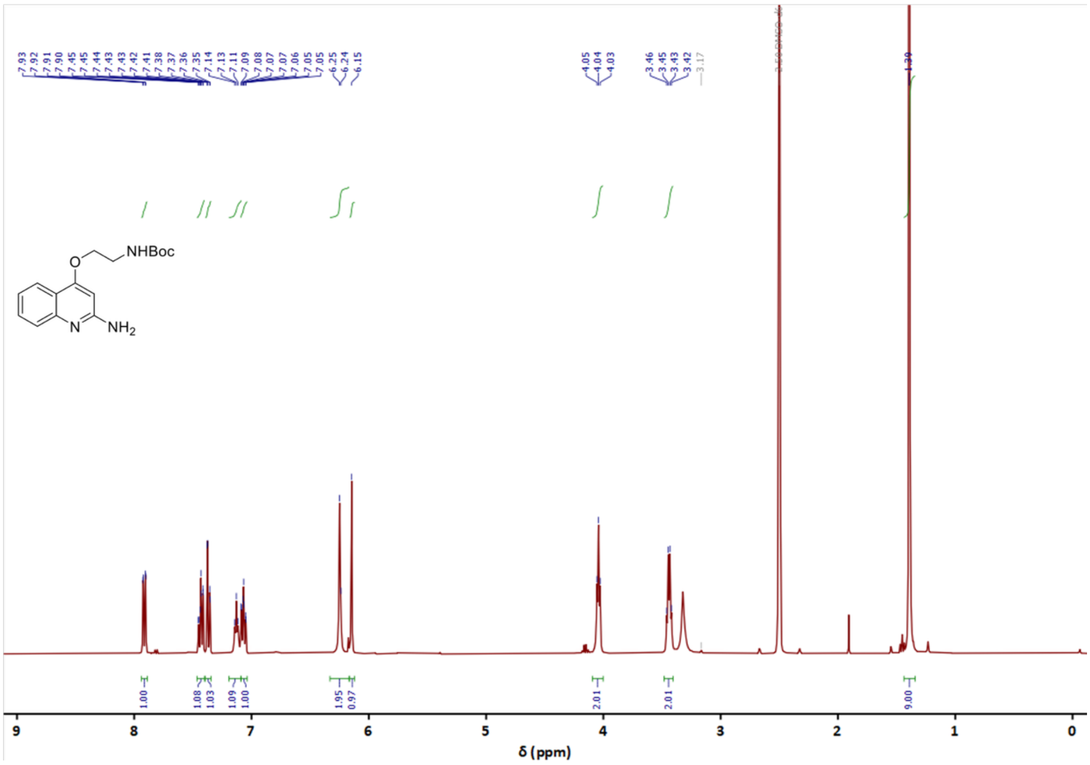

^1H NMR spectrum of **15** in DMSO-d_6 measured at 400 MHz.

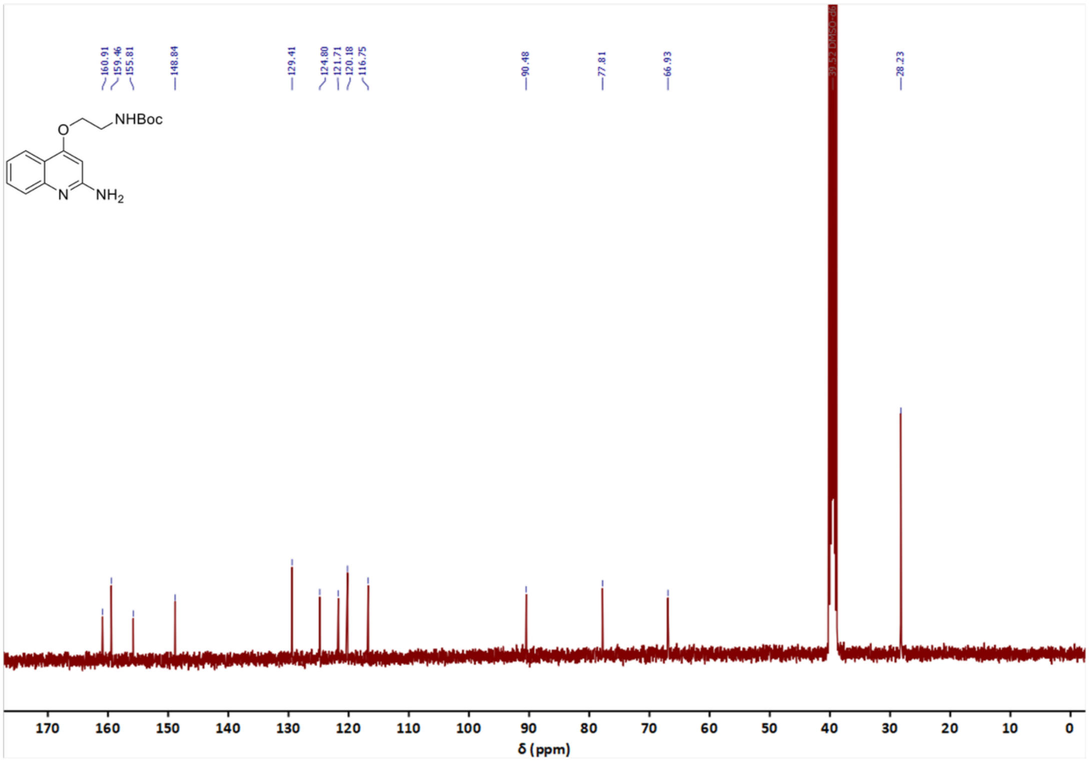

^{13}C NMR spectrum of **15** in DMSO-d_6 measured at 100 MHz.

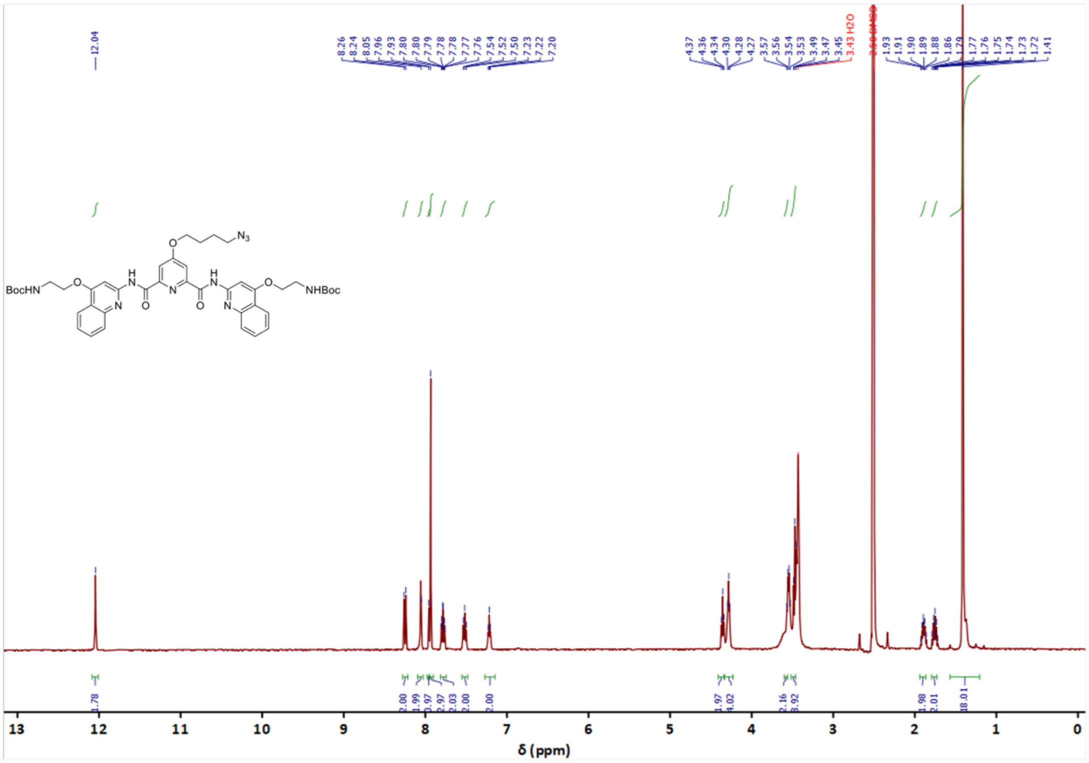

^{1}H NMR spectrum of **16** in DMSO-d_6 measured at 400 MHz.

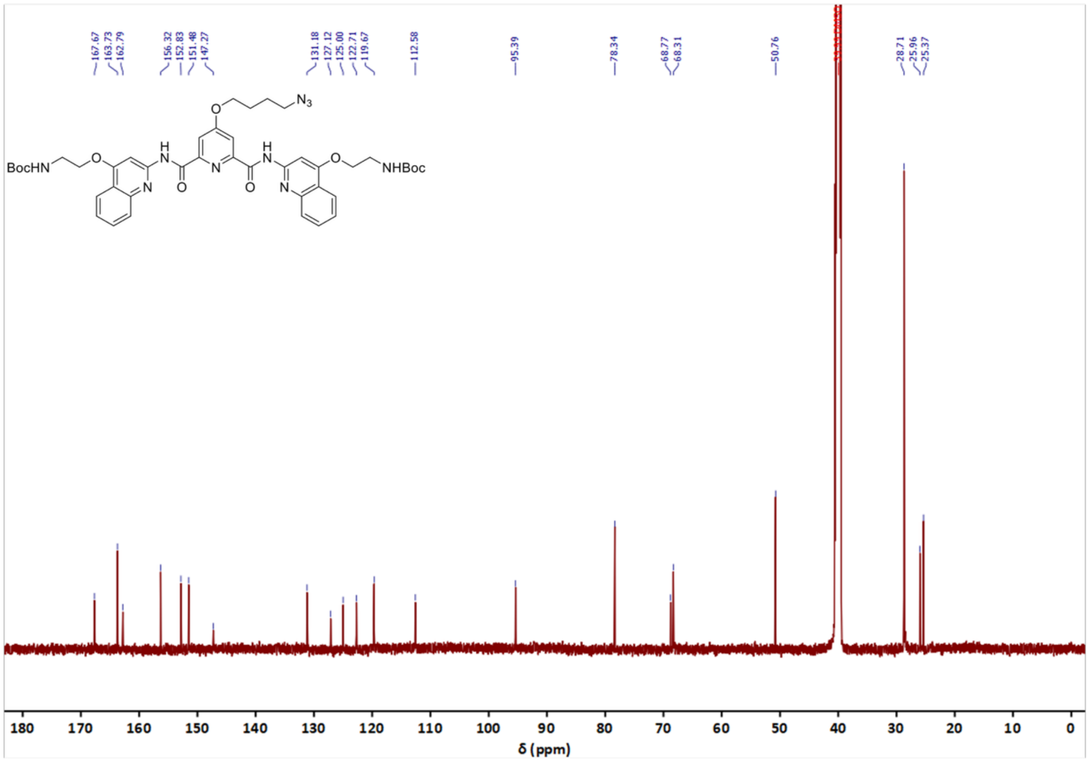

^{13}C NMR spectrum of **16** in DMSO-d_6 measured at 151 MHz.

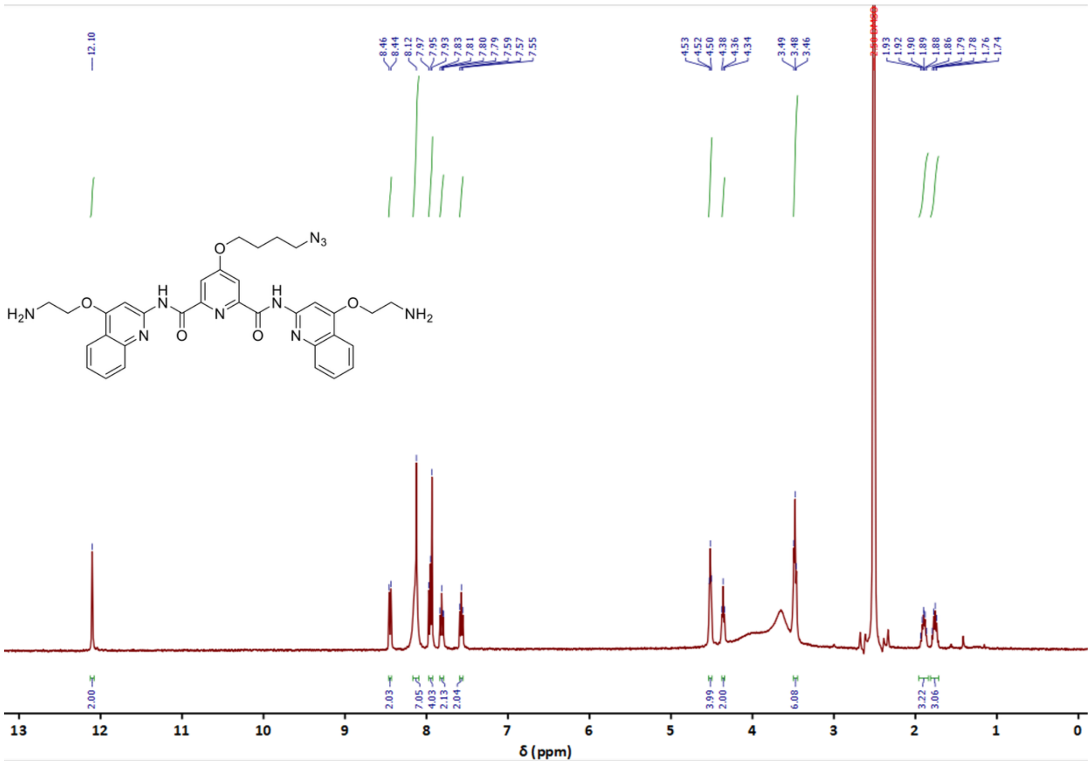

^{1}H NMR spectrum of **GL7** in DMSO-d_6 measured at 400 MHz.

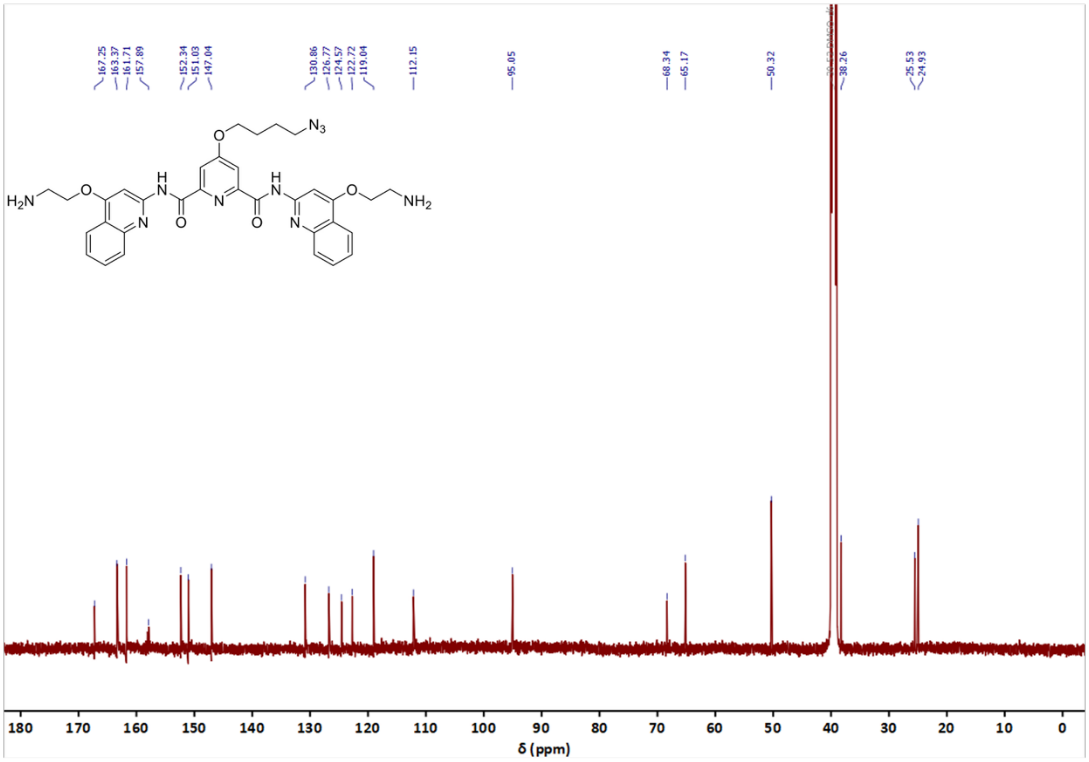

^{13}C NMR spectrum of **GL7** in DMSO-d_6 measured at 151 MHz.

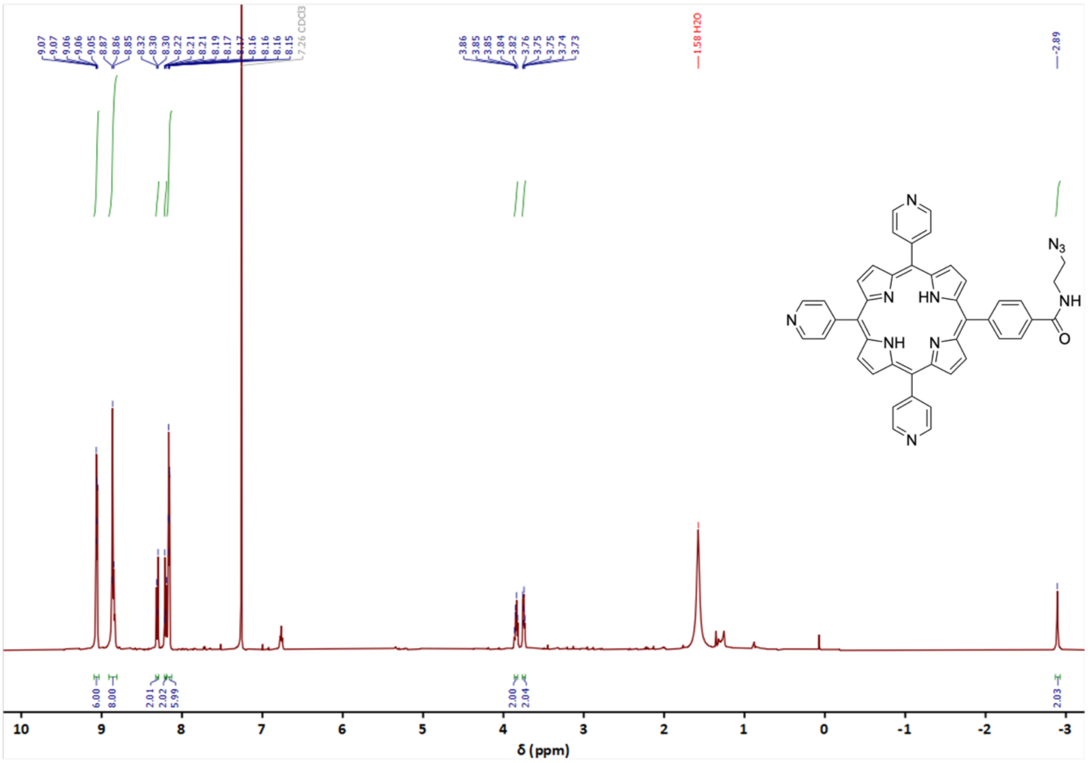

^{1}H NMR spectrum of **18** in chloroform-d measured at 400 MHz.

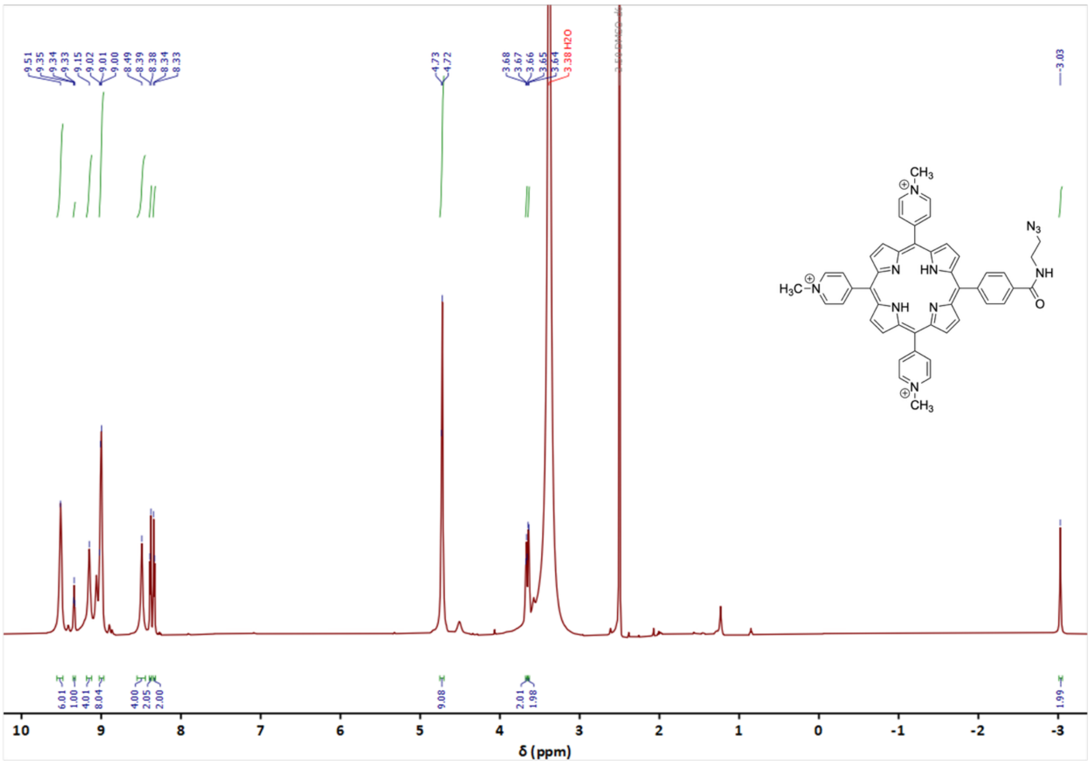

^{1}H NMR spectrum of **GL8** in chloroform-*d* measured at 600 MHz.

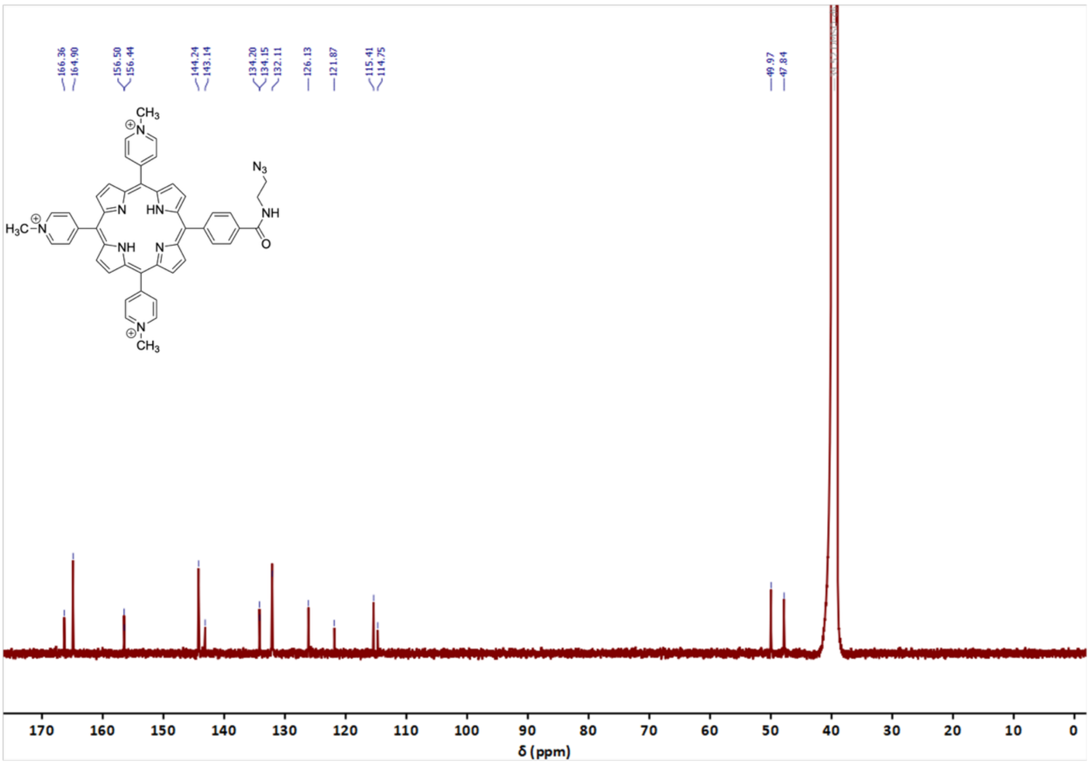

^{13}C NMR spectrum of **GL8** in DMSO-d_6 measured at 151 MHz.

Conjugation

5´-hexylamine oligonucleotide (Na^+ salt, 50 nmol) was dissolved in 0.1 M $NaHCO_3$ (aq) (50 µL, 1 mM). A solution of (1R,8S,9s)-bicyclo[6.1.0]non-4-yn-9-ylmethyl N-succinimidyl carbonate (BCN-NHS ester) (20 equiv., 5 µL, 0.2 M) was freshly prepared and added to the oligo solution. The conjugation reaction was vortexed and shaken for 24 h. Followed by precipitation of oligo using 10% v/v of 3 M NaOAc and 4 times the volume EtOH and kept in freezer for 30 min. Then the solution was centrifuged for 15 min followed by removing the supernatant. The precipitate pellet was resuspended in the same volume of NaOAc and EtOH and repeated the precipitation. After this, the pellet was washed with only EtOH followed by drying the pellet by gently adding N_2 gas and then resuspended in 0.1 M $NaHCO_3$ (aq) (50 µL, 1 mM). The G4-ligand (2.5 equiv., 25 µL, 10 mM in DMSO) was added to the dissolved oligo and vortexed and shaken for 24 h. The reaction mixture was dissolved in more water and DMSO and filtered before purified by RP-HPLC. Purification was done using a C18 semi-preparative column and 2 mL/min flowrate and a linear gradient of 5 to 60% solvent B (100% ACN). Solvent A was a 50 mM triethylammonium acetate buffer. The conjugation products were confirmed with HRMS (ESI^-).

Chromatograms of the GL-O conjugates

GL-O1

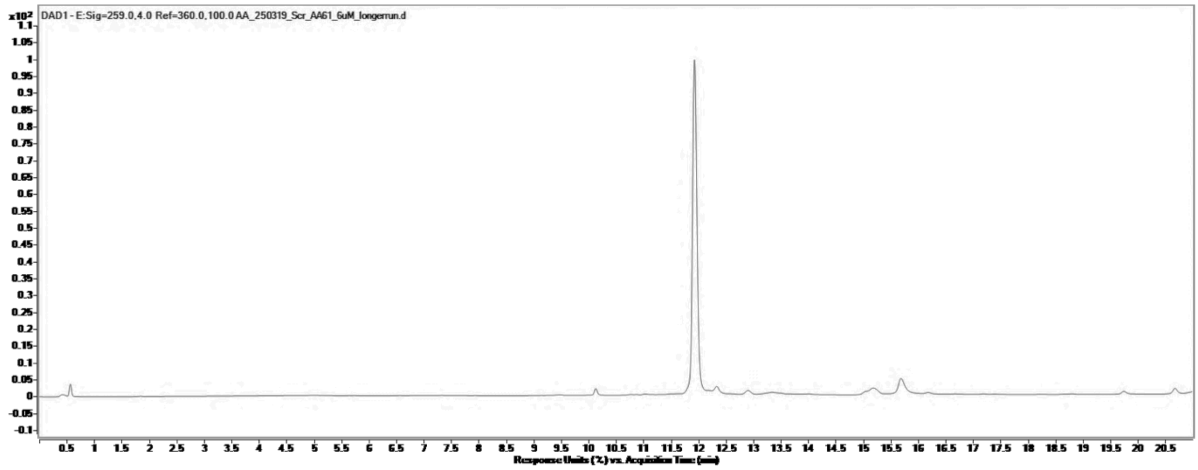

GL-O2

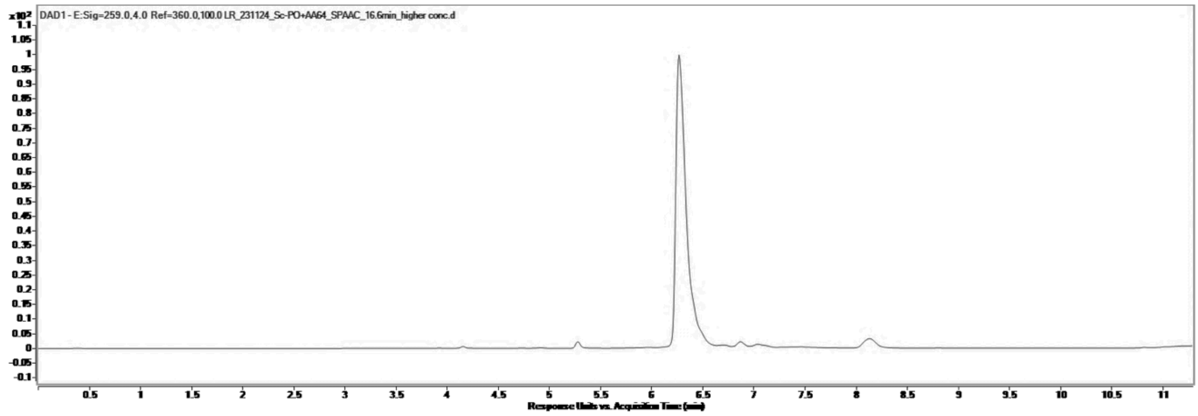

GL-O3

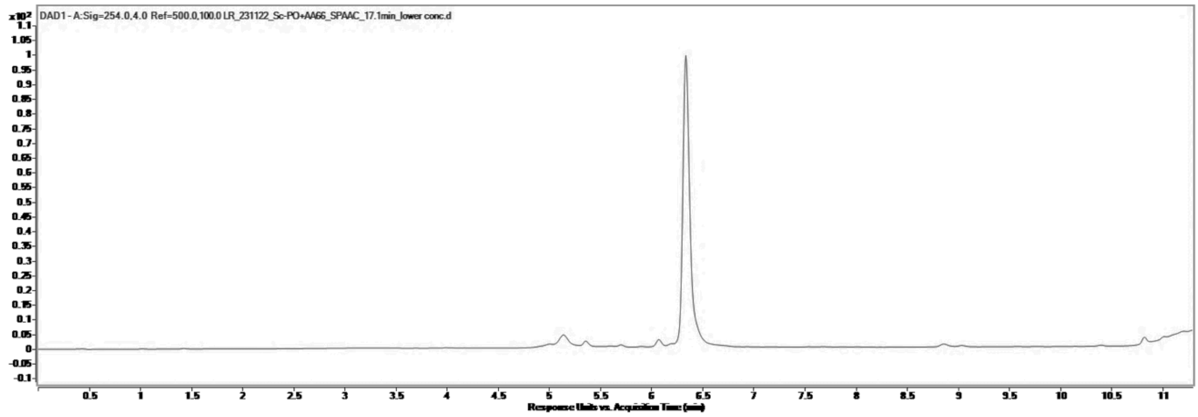

GL-O4

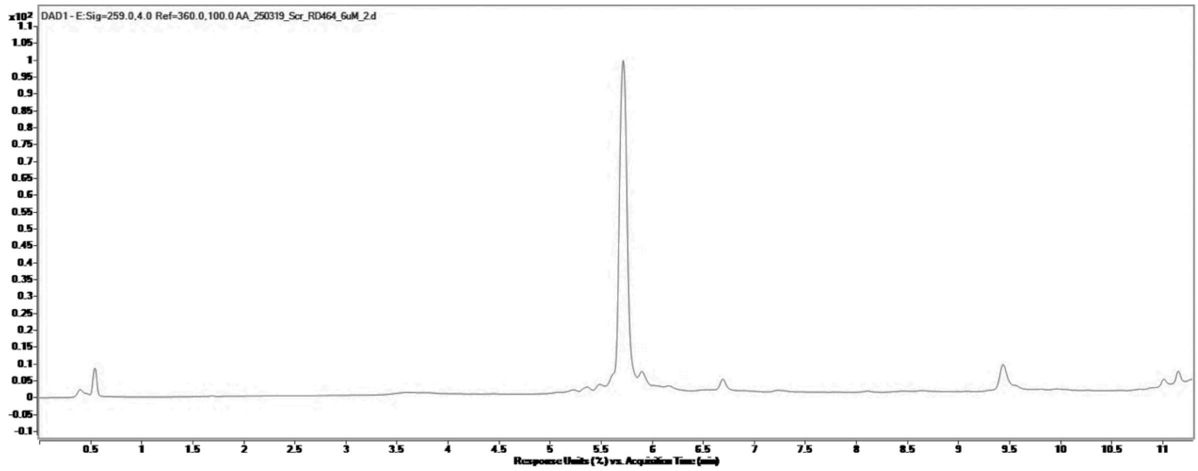

GL-O5

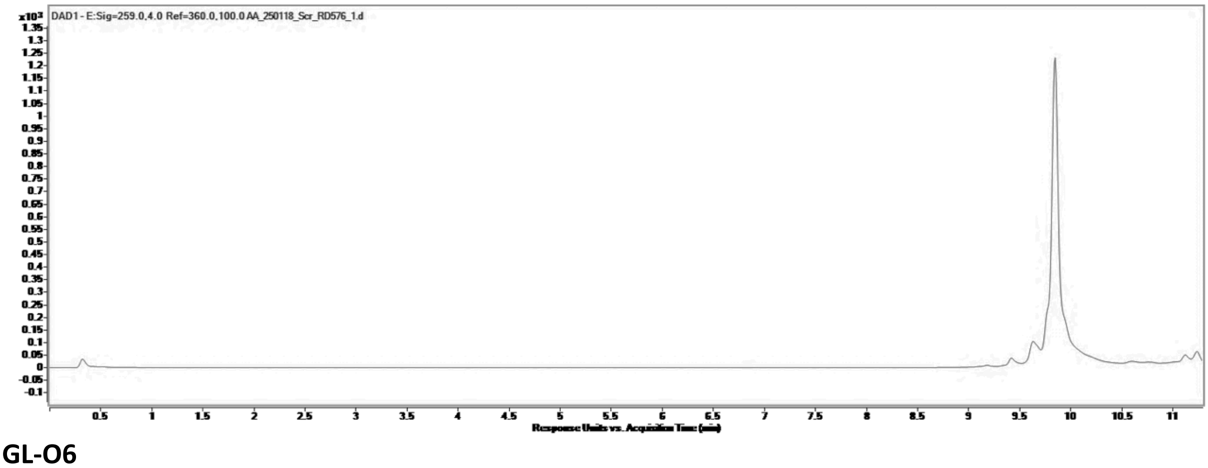

GL-O6

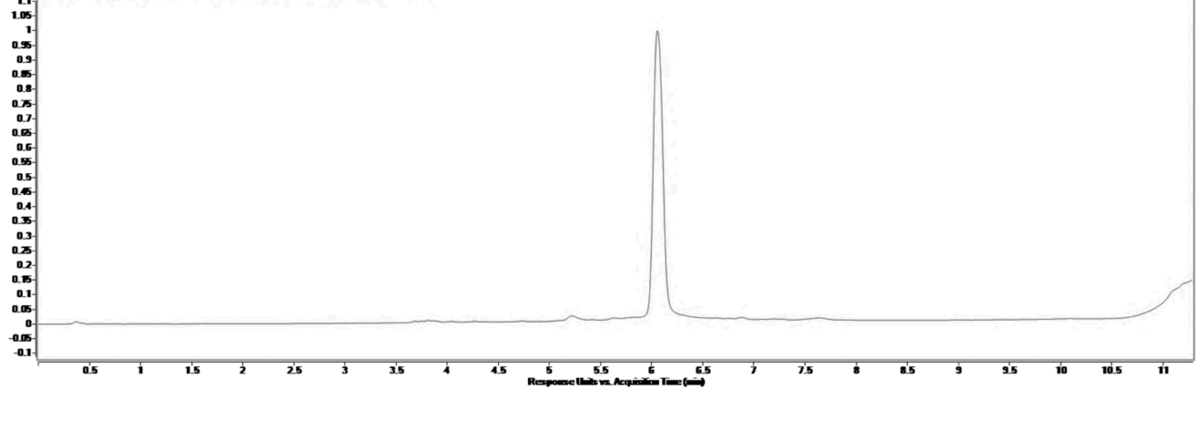

GL-O7

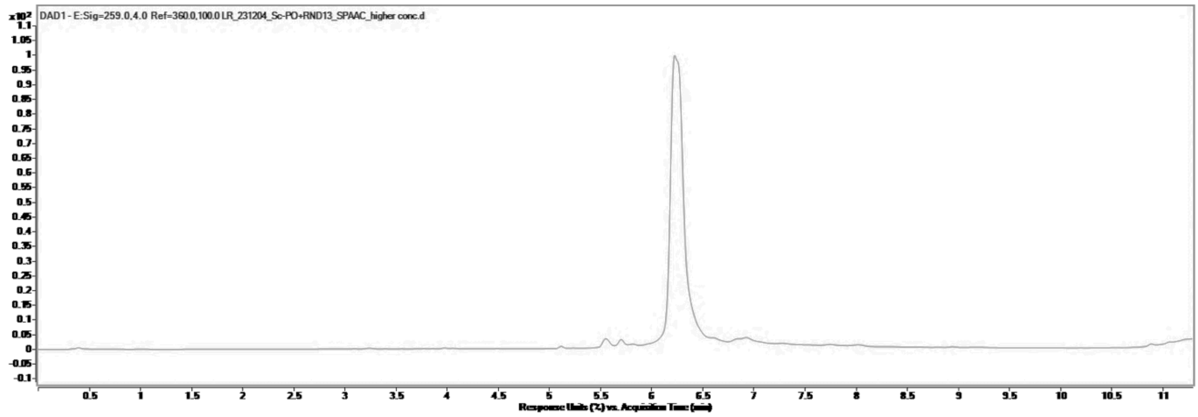

GL-O8

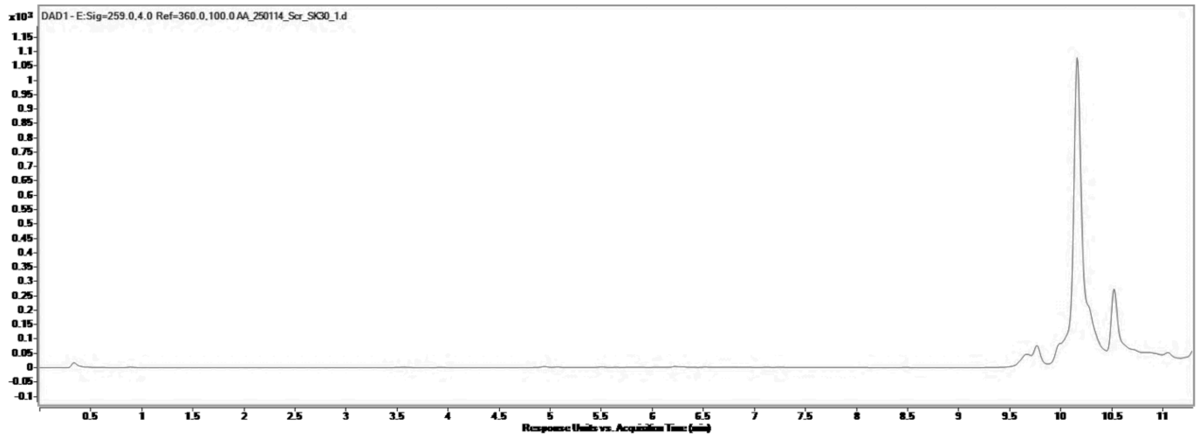

GL-O9

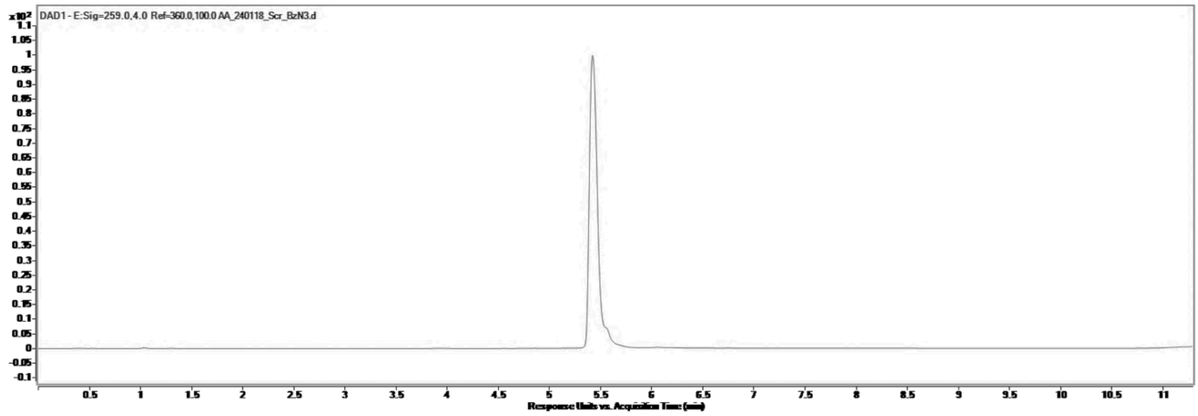

Table S4. Yields and MS of the GL-O conjugates.

Conjugate name	Yield	Calc. MS (g/mol)	Found MS (m/z)
**GL1**	42	5299.90	5299.16
**GL2**	68	5299.90	5299.14
**GL3**	55	5257.82	5257.01
**GL4**	32	5484.10	5483.33
**GL5**	60	5596.31	5596.35
**GL6**	70	5513.18	5513.14
**GL7**	44	5518.06	5517.18
**GL8**	63	5639.29	5640.41
**GL9**	91	5000.52	4999.93

In vitro assays

Microscale thermophoresis (MST). The G4 DNA template contains a 5´-labelled Cy5 tag and consist of the G4 with or without flanking sequence (Table S1). The G4 DNA was annealed in MST buffer (10 mM potassium phosphate, 100 mM KCl, 0.05% Tween 20, pH 7.4) by heating at 96 °C for 5 min followed by cooling down to room temperature before storing in fridge overnight. All MTS experiments were done on a Monolith NT.115 (Nanotemper, Germany) instrument and performed in MST buffer. The G4 DNA concentration was kept constant at 20 nM to which ligands/conjugates were serial diluted (1:1) with highest concentration of 40/20 µM.

MST traces and binding constants (K_D) were obtained using the Monolith analysis software. K_D values were calculated using a custom on time of 1.5 s, with the target concentration fixed at 20 nM. The binding curves were plotted and visualized in GraphPad Prism 10 and fit using a 1:1 binding model, except for **GL6** in Figure S1B.

Taq polymerase STOP assay. Taq Polymerase stop assay was adapted from Jamroškovič et al.[1] DNA templates were annealed to fluorescent labelled primers in 100 mM KCl by heating to 95 °C for 5 minutes followed by slow cooling to room temperature. The indicated compound concentrations were added to 40 nM annealed template in 1x Taq Buffer (10 mM Tris-HCl pH 8.8, with and without 50 mM KCl, Thermo Fisher Scientific), 1.5 mM $MgCl_2$, and 0.05 U/µL Taq Polymerase (Thermo Fisher Scientific). Samples were preincubated on ice (10 minutes) and reactions initiated with the addition of dNTPS (100 µM) and transferring the samples to 37 °C. After 15 min at 37 °C, reactions were stopped by addition of equal volume of 2x stop solution (0.5% SDS, 25 mM EDTA, XC-Dye in Formamide) and separated on a 12% polyacrylamide Tris-Borate-EDTA (TBE) gel containing 25% formamide and 8 M urea. Fluorescent signal was detected with a Typhoon Scanner (Amersham Biosciences). The intensity of the full-length band was quantified using Image Quant TL 10.2 software (GE Healthcare Life Sciences) and compared to sample without compound.

NMR titrations and thermal stability assay. G4 DNA solution was annealed in 10 mM potassium phosphate buffer (3 mM KCl, 110 µM G4 DNA, pH 7.4) by heating at 96 °C for 5 min followed by cooling down slowly to room temperature and stored in fridge overnight. To the G4 DNA solution (180 µL), D_2O (20 µL) was added to yield a 100 µM solution of G4 DNA which was transferred to a 3 mm NMR tube. GL-O (1 mM in H_2O) was added (20 µL) to the NMR tube, yielding a 1:1 molar ratio with the G4 DNA. After waiting 10 min, a ^1H NMR spectrum was recorded. The same was done with G4-ligands (10 mM in DMSO) (2 µL). The instrument setting used was an 850 MHz Avance III HD spectrometer equipped with a 5 mm TCI cryoprobe at 298 K. Transmitter frequency offset (O1P) was set at 4.7 ppm and spectral width (SW) was fixed as 22 ppm. Excitation sculpting was used in the 1D ^1H experiments, and 512 scans were used to record the spectra.

For the thermal stability assay, the samples were equilibrated at temperatures ranging from 313-338 K for 5 min followed by recording of the spectra every 5 K interval. The data was processed with MestreNova 10.0.2.

References

1. A. Berner, R. N. Das, N. Bhuma, J. Golebiewska, A. Abrahamsson, M. Andréasson, N. Chaudhari, M. Doimo, P. P. Bose, K. Chand, R. Strömberg, S. Wanrooij and E. Chorell, *J. Am. Chem. Soc.*, 2024, **146**, 6926-6935.
2. M. Andreasson, M. Donzel, A. Abrahamsson, A. Berner, M. Doimo, A. Quiroga, A. Eriksson, Y.-K. Chao, J. Overman, N. Pemberton, S. Wanrooij and E. Chorell, *J. Med. Chem.*, 2024, **67**, 2202-2219.

3. B. Prasad, M. Doimo, M. Andréasson, V. L’Hôte, E. Chorell and S. Wanrooij, *Chem.Sci.*, 2022, **13**, 2347-2354.
4. C. Spagnul, R. Alberto, G. Gasser, S. Ferrari, V. Pierroz, A. Bergamo, T. Gianferrara and E. Alessio, *J. Inorg. Biochem.*, 2013, **122**, 57-65.
